# The Community Pediatrics Training Initiative Project Planning Tool: A Practical Approach to Community-Based Advocacy

**DOI:** 10.15766/mep_2374-8265.10630

**Published:** 2017-09-18

**Authors:** Benjamin D. Hoffman, Jerri Rose, Debra Best, Julie Linton, Steven Chapman, Michele Lossius, Andrew Aligne, Cappy Collins, Lisa Ayoub-Rodriguez

**Affiliations:** 1 Professor of Pediatrics, Oregon Health & Science University School of Medicine; 2 Associate Professor of Pediatric Emergency Medicine, Rainbow Babies and Children's Hospital; 3 Associate Professor of Pediatrics, Duke University School of Medicine; 4 Assistant Professor of Pediatrics, Wake Forest School of Medicine of Wake Forest Baptist Medical Center; 5 Assistant Professor of Pediatrics, Geisel School of Medicine at Dartmouth; 6 Associate Professor of Pediatrics, University of Florida College of Medicine; 7 Assistant Professor of Clinical Pediatrics, University of Rochester School of Medicine and Dentistry; 8 Assistant Professor of Population Health, Mount Sinai Health System; 9 Assistant Professor of Pediatrics, Texas Tech University Health Sciences Center Paul L. Foster School of Medicine

**Keywords:** Advocacy, Pediatrics, Community Health, Milestones

## Abstract

**Introduction:**

To impact social determinants of health, physicians require knowledge, skills, and attitudes to work with communities beyond the clinical milieu. The American Academy of Pediatrics (AAP) Community Pediatrics Training Initiative (CPTI) project planning tool can guide health care professionals and trainees to identify and define issues, build coalitions, assess interventions, and ensure sustainability of successful programs. The Accreditation Council for Graduate Medical Education guidelines for pediatric training require experiences in community health. To date, there have been no widely available tools to ensure both robust learning and validated assessment for pediatric residents in community pediatrics and advocacy training.

**Methods:**

The AAP CPTI project planning tool engages learners with a step-by-step process involving investigation, guided reflection, and structured assessment. Learners practice the skills necessary to plan, implement, and evaluate a community pediatrics/child health advocacy proposal focused upon a learner-defined area of interest. An assessment rubric maps to milestones.

**Results:**

This project planning tool has been used in a number of programs with learners at multiple levels, including undergraduate education, graduate education, and practicing health care providers. It can be employed to design and implement a community advocacy intervention or as a thought exercise and can be incorporated in a single block rotation or as a longitudinal experience. It can be used with individual learners or as a group exercise.

**Discussion:**

The project planning tool can be used by residency programs to demonstrate resident competence in community health and advocacy, either as a learning exercise or to guide actual implemented projects.

## Educational Objectives

After completing this activity, learners will be able to:

1. Identify a specific, focused area of interest related to child health and well-being.
2. Obtain population-level data and complete a literature review to build expertise about the identified area of interest related to child health and well-being.
3. Identify key stakeholders within the institution and community in regard to the identified child health issue, list these individuals, and note shared values and goals.
4. Define focused and measurable SMART objectives for a community-based intervention as they relate to the defined child health advocacy topic.
5. Develop a draft plan for a community-based intervention designed to lead to a desired change that meets the defined, measurable objectives.
6. Employ quality-improvement methodology as it relates to evaluation of the theoretical community-based advocacy intervention.
7. Reflect on plans for actual involvement in child health advocacy as part of their professional role as a pediatrician.

## Introduction

Pediatricians at all levels of training fundamentally engage in advocacy efforts on behalf of patients who cannot advocate for themselves. Thus, the field of pediatrics is a natural and essential setting to develop and implement tools to train health care professionals regarding child health advocacy. Since 2001, the Accreditation Council for Graduate Medical Education (ACGME) Pediatric Residency Review Committee has required that pediatric residency programs provide educational experiences that prepare residents for the role of advocate for the health of children within the community. Current ACGME program requirements for pediatric residency education require two educational units that incorporate elements of community pediatrics and child advocacy in the curriculum.^1^ Despite this requirement, there is little consensus about how community pediatrics and child advocacy should be taught, learned, and evaluated during pediatric residency training. Although guidelines for the development of community pediatrics and child health advocacy curricula have been developed,^2^ there is a relative scarcity of educational tools available to help train learners in basic principles related to community health and advocacy. While several reports have described advocacy training within the context of pediatric residency programs, no standard curriculum exists.^3^

An examination of strategic approaches and successful models for education in community health and child advocacy cited a number of effective strategies for engaging pediatric residents in community pediatrics training and outlined key curricular components needed to achieve resident competency in these areas.^4^ These key curricular components include structured, coordinated experiences with clear goals and objectives, exposure to community health outside of the traditional hospital or private practice setting early in training, resident projects in longitudinal or block form, and integration of community pediatrics and advocacy within other educational rotations and pediatric divisions.

The learning activity described here aligns with the evidence-based recommendation that resident projects in longitudinal or block form be incorporated as a strategy for engaging residents in community pediatrics and advocacy training. While programs have developed a variety of approaches for this strategy, there is a paucity of peer-reviewed literature describing them. One study evaluated the breadth of advocacy topics chosen by residents over the course of residency training at three separate institutions supported by a longitudinal child advocacy curriculum.^3^ The wide range of topics and settings in which residents developed projects indicated that pediatric residents have “extensive interests and ingenuity in applying needed advocacy solutions to complex child health issues.”^3^ Another program described a longitudinal community-based advocacy curriculum for pediatric residents that involved implementation of community-based projects by residents in their second and third years of training (during 2-week blocks scheduled during each of these years).^5^ These authors concluded that the community-based project experience “had a noticeable positive influence on resident attitudes toward community-centered advocacy.”^5^ A consensus-based working group ultimately developed best-practice training objectives in community health and advocacy and linked those objectives to ACGME assessment milestones.^6,7^ Identification of curricular activities that facilitate resident training to achieve the objectives can thus benefit not only the learner but also the training program as a means of meeting ACGME assessment requirements. The Community Pediatrics Training Initiative (CPTI) Child Health and Advocacy Milestones Profile (CHAMP) can be found in this resource as Appendix A, with the associated CHAMP mapping tool as Appendix B.

While completing a community-based project has been shown to improve resident attitudes toward community-centered advocacy,^8^ many programs do not have the curricular time or faculty capacity to mentor trainees in the completion of an implemented community project. The learning resource described here was designed to lead pediatric residents through the process of planning, designing, and evaluating a community-based advocacy project either as a hypothetical thought exercise or for an actual implemented project using a step-by-step approach. This allows program leadership and teaching faculty to design learning experiences that offer active learning of practical knowledge and implementable skills in community health and advocacy regardless of the requirement for completion of an actual advocacy project. This resource was originally developed for use within the context of a block rotation in community pediatrics/child health advocacy. However, it could certainly be adapted for use within a more longitudinal curriculum, such as over the course of a 3-year residency program or even a single year of training. While originally designed for pediatric residency training, this educational tool and learning activity have great potential for use in training residents in other specialties, for other learners in the health professions (including medicine, nursing, public health, and social work), or even for practicing health professionals.

## Methods

This project planning tool (Appendix C) engages residents in a guided, step-by-step learning activity involving independent investigation, facilitated reflection, and reflective writing exercises that provide the opportunity to learn and practice the skills necessary to plan, implement, and evaluate a community pediatrics/child health advocacy proposal focused upon a learner-defined area of interest. The project proposal could remain a thought exercise or be used to actually design and implement a community advocacy intervention. The tool could be used equally effectively in the context of a single block rotation or as a longitudinal experience and equally easily as an individual or a group exercise.

This activity is typically introduced to learners through an initial 45- to 60-minute orientation session. The session covers the role pediatricians and other health professionals must play as advocates for the health of children in their community; introduces the process of planning, implementing, and evaluating community-based projects; and explains the objectives and expectations for the learning activity. While we have generally reviewed the tool one-on-one with learners, it is also possible to employ a more formal presentation, which may prove especially useful for larger groups. This presentation could be created through modification of the PowerPoint presentation included as a user's guide (Appendix E). Following this introductory didactic session, learners work independently, in pairs, or in small groups to complete the 10 steps described in the project planning tool over the course of their experience, checking in with and seeking guidance from supervising faculty members as needed. In programs where the project planning tool is completed in a single block rotation, there are regular (usually weekly) meetings to review progress, answer questions, and allow for reflection by the learners.

The tool itself includes 10 discreet sections, each with one or more activities that walk the learner through each component step of the process. The activities all include specific instructions, illustrations, and examples and require some product as an output. This product may take the form of a specific written task, a reflective writing exercise, or concrete output such as a bibliography. The 10 steps of the project planning tool are as follows:

1. Identify the problem.
2. Define the baseline.
3. Learn the literature.
4. Explore existing resources.
5. Develop your road map.
6. Build the coalition.
7. Ensure things are done with the community, not to the community.
8. Work diligently to accomplish goals and objectives.
9. Develop tools for effective evaluation.
10. Regularly reevaluate and reflect on plan and project-related work.

At the conclusion of the experience, a debriefing session is held, during which learners are asked to share a summary of the details of their community-based project proposal, including their reflections on lessons learned through completion of the process and the impact of this experience on their future practice. This can be done as a one-on-one session or a group discussion, depending on the number of learners and the structure of the learning experience within which the project planning tool is incorporated. During the course of the debriefing session, both resident peers and faculty have an opportunity to ask questions and provide constructive feedback and suggestions regarding each of the resident projects presented. Note that the length of the debriefing session depends on the number of learners completing the activity.

For curricula that may require actual implementation of the proposal as a project, the subsequent steps can be defined as needed.

The project workgroup also developed an assessment rubric (Appendix D) to accompany the project planning tool. The rubric features a competency spectrum the faculty supervisor can use to assess the learner based on behavioral and cognitive anchors. Using the CHAMP mapping tool (Appendix B), the workgroup utilized a modified Delphi approach to link each of the individual assessment outcomes in the rubric to ACGME milestones for pediatric residency training.^7^ This assessment rubric enables faculty not only to assess the learner's knowledge, skills, and attitudes in child health and advocacy but also to employ the learning experience toward the assessment of the resident learner in achieving competence and demonstrating achievement of milestones as part of the overall residency program assessment paradigm.

We have also included a user's guide (Appendix E), designed for faculty who may be incorporating the project planning tool in their curricula or programs. It consists of a PowerPoint presentation that walks faculty through the basic structure and design of the project planning tool and provides guidance on how the tool can be incorporated within a curriculum, either to frame a project or as an academic exercise. This presentation can also be modified to introduce the tool to learners. We encourage users to modify this presentation to suit their needs.

### Faculty Background and Training Required

Successful implementation of this curriculum requires at least one faculty mentor to guide the learners through the process. We suggest that this faculty leader have familiarity with the CPTI CHAMP and the associated mapping tools (Appendices A & B), as these can help identify both how and where in the training curriculum the learning objectives can be taught and assessed. Furthermore, it would be beneficial for that faculty member to have some facility with resources and contacts within his/her own community to support learners in their program as they progress through the tool. Finally, effective faculty have often themselves engaged in advocacy efforts and thus understand the challenges and opportunities that often emerge during project planning and implementation.

Faculty members may find that using the CPTI project planning tool themselves helps to develop their curricula and strategies for effective incorporation of the tool into the training of future child health advocates.

## Results

This curricular resource was initially developed in 2002 and has been utilized as part of the community health and advocacy curriculum at the University of New Mexico School of Medicine's pediatric residency program since. Between 2002 and 2011, University of New Mexico residents employed the tool in a number of ways to develop and implement community-based initiatives that included seven American Academy of Pediatrics (AAP) Community Access to Child Health (CATCH) grants, two AAP National Resident Advocacy Awards, and a number of successful nonfunded community partnerships that impacted child health and well-being. The tool has been informally shared with a number of residency programs across the country since 2011, allowing feedback that has enhanced the tool as it evolved and became more generalizable. Currently, it is being formally used as part of community health and advocacy training at a number of pediatric residency programs.

Comments from second-year residents at Oregon Health & Science University, who completed the project planning tool between 2014 and 2016, reveal its perceived benefits, including providing a framework for learning and for experiencing advocacy virtually:

• “What ended up being most helpful was really just getting started and trying to work on things in a step-wise fashion. I enjoyed working on the mission and values with the project planning tool; it reminded me of all the reasons I enjoy medicine outside of simply the clinical work.”
• “Completing the project planning tool improved my understanding of the skills and mindset necessary to be an advocate on a broader, community level, and to put thoughts into action. I am very likely to submit a grant in the future.”
• “What I really learned is understanding what advocacy is: fascinating. I loved the readings and trying to pull in leadership qualities with great project ideas, but putting something into action that won't just satisfy a need I have but really help others that is exceptionally hard work. You can't be an advocate for children by yourself and you really need to include other people.”
• “By participating in the development of an advocacy proposal I learned that advocacy doesn't end at ‘it's important’ but requires identifying an issue (large or small), understanding who it effects, and intervening in a community-minded fashion. It will teach me how to find resources in my community pertaining to childhood health. The knowledge and skill to put together an advocacy project are useful for every pediatrician. No matter what community or patient population I serve in my career I know that childhood health does not end when they walk out of the office or hospital.”

In some of the programs, the tool is utilized to plan a required project. In others, it functions as a more theoretical project planner. In programs that require a scholarly project, the project planning tool has been employed as the framework for developing community health and advocacy work that meets the requirements for scholarship.

• “At the Wake Forest Pediatric Residency Program, residents participate in a longitudinal advocacy curriculum. Wake Forest residents have been engaged in individual or paired advocacy projects for more than a decade. During the advocacy and chronic care rotation, as well as throughout the first year, residents meet with the advocacy director to identify areas of interest and begin to select (or plan) a project. The project planning tool now serves as the foundation for this orientation. The project planning tool has enriched the planning, implementation, and evaluation process.”
• “The Dartmouth Pediatric Residency uses the tool as the framework for a longitudinal community pediatrics curriculum through all 3 years of residency. In addition to a block experience in each year, elements are integrated into other rotations including behavior and development, adolescent, and an elective in global health. A core group of advocacy faculty have been identified, including both traditional medical-center based faculty as well as community agency faculty, who serve as mentors and advisors for projects.”
• “The Duke Pediatric Residency requires that residents complete a 1-month rotation in community pediatrics and advocacy during their second or third years of residency. Residents are required to complete the project planning tool as an educational exercise designed to teach residents the knowledge, skills, and attitudes necessary to develop and implement a community-based advocacy project. Several residents have also used this as a framework in developing CATCH grant applications.”

The project planning tool is also being used by faculty development programs in pediatrics and other specialties as part of their curricula. The Duke Endowment-funded Carolinas Collaborative, a collaborative among all eight pediatric residency training programs in the Carolinas, is using the tool to help each of the participating sites identify and develop community-based educational curricula designed to teach residents the knowledge, skills, and attitudes necessary to be effective advocates for children in the context of projects that address toxic stress in their communities.

The Missouri Collaborative for Advocacy and Resident Education (MOCARE) is a collaborative of the four pediatric residency training programs in Missouri, funded by the Missouri Foundation for Health. MOCARE is using the tool as a leadership development exercise for involved faculty and residents, focusing on improving resilience of families in the communities the programs serve.

The Oregon Health Care Quality Corporation is utilizing the tool as a part of a longitudinal physicians' academy, working with rural primary care physicians and allied health professionals in a yearlong fellowship designed to empower them to become effective community advocates and leaders. The CPTI project planning tool is being used by each of the fellows to design and implement community-based interventions.

At our institution, this project planning tool has been utilized not only for resident advocacy training but also for faculty development and undergraduate medical education. The tool provides a structure for faculty development presented during a faculty meeting. Additionally, for medical students or other learners planning and implementing advocacy projects in the community, the tool offers a structured model to effectively incorporate concepts that may be overlooked by a novice, such as incorporating stakeholders from the beginning, developing measurable objectives, and considering evaluation before project implementation. The project planning tool has enhanced the effectiveness of a long-standing advocacy curriculum that has become part of the culture of our pediatric training program.

Our intent in developing this project planning tool has been to create a versatile and useful curricular activity to frame community health and advocacy. Among us, it has been used for single learners, groups, and block or longitudinal experiences. Its adoption as a faculty development tool and as a part of undergraduate medical education suggests its versatility. The fact that it has been successfully incorporated in such disparate ways demonstrates that our intent has been realized. We have learned much from each other about how the tool has been utilized and adapted to meet the needs of learners in different programs or with different needs. We hope that as others begin to employ the project planning tool, they will share with us their experiences and suggestions, as we continue to learn how to better teach physicians and others how to better collaborate with their communities.

## Discussion

The CPTI project planning tool has been effectively employed at multiple levels, from graduate medical education to faculty development and community-based, quality-improvement development programs. While the tool was initially developed at a single institution, our affiliation with the CPTI has allowed us to collaborate with a number of colleagues across the country, resulting in a product easily adaptable to any learning environment and for any community.

Through the development and early dissemination of the CPTI project planning tool, we have been able to iteratively transform the process and edit the content to create a tool that has been effectively used across different programs and for different learner levels and professions. It has been fascinating to witness the tool's evolution from a single institution's exercise for pediatric interns. Each program adopting the tool has employed it differently, yet it has proven to be adaptable. We are very pleased with the ease with which it has been able to meet the needs of disparate curricula to help learners across varied spectra achieve desired levels of competence, from practice to implementation of community health and advocacy principles.

While the project planning tool has been incorporated into a number of training programs, there are some limitations to its use. First, although we have informal data on learner satisfaction, we lack quantitative data from learners about their experience. Second, while the assessment rubric was developed by an expert panel, it has not been validated. Third, while the tool can be used in a number of ways, with different learners, and in varied curricular environments, most of its implementations have been in the realm of child health, and we do not have enough experience with the tool in other specialty areas, in either medicine or other health-related disciplines, to be able to anticipate what challenges might arise.

It is our expectation that further dissemination and incorporation will yield further evolution and adaptation. As health systems move to a population health approach, we remain very excited by the prospect of further adoption by undergraduate and graduate medical education curricula of a tool that can help transform not only training but also professional practice. The evaluation rubric can be adapted to meet each user's assessment requirements, be they competencies, milestones, or entrustable professional activities.

By demonstrating to learners that advocacy is an essential art of a health care provider's identity and professional responsibility and giving them concrete tools with which they can collaborate with their communities, the CPTI project planning tool can become a key curricular element in every training program's portfolio. Through the CPTI, we hope to establish a collaborative, internet-based resource enabling users of the project planning tool to share their adaptations and discuss their successes and suggestions for change.

## Appendices

A. CHAMP.pdf
B. CHAMP Mapping Tool.pdf
C. AAP CPTI Project Planning Tool.docx
D. AAP CPTI Project Planning Tool Milestones-Based Assessment Rubric.docx
E. Project Planning Tool Users Guide.pptx All appendices are peer reviewed as integral parts of the Original Publication.
