# Supplementary material for: The Community Pediatrics Training Initiative Project Planning Tool: A Practical Approach to Community-Based Advocacy
Source: MedEdPORTAL. 2017 Sep 18;13:10630. doi: 10.15766/mep_2374-8265.10630 (PMC6338167; doi:10.15766/mep_2374-8265.10630)
Supplement: Supplementary file 1 — A. CHAMP.pdf B. CHAMP Mapping Tool.pdf C. AAP CPTI Project Planning Tool.docx D. AAP CPTI Project Planning Tool Milestones-Based Assessment Rubric.docx E. Project Planning Tool Users Guide.pptx [file mep-13-10630-s001.zip › A. CHAMP.pdf]

CHAMP

AAP CPTI Community Health and Advocacy Milestones Profile

- All **BOLDED** competencies are among the 21 Milestones that need to be reported to the ACGME presently.
- The **BLACK text** indicates the competency number from the original ABP Milestones Document, 2012.
  - https://www.abp.org/abpwebsite/publicat/milestones.pdf
- The **RED text** indicates the competency number from January 2013 regarding reporting upon the first 21 of the 51 total Milestones if it differs from the competency number assigned in the original ABP Milestones document. These are the Milestones that are currently being reported.
  - http://acgme.org/acgmeweb/Portals/0/PDFs/Milestones/PediatricsMilestones.pdf

CPTI Community Health and Advocacy Milestones Profile

Goals & Objectives

A. Culturally Effective Care

Pediatricians must demonstrate skills that result in effective care of children and families from all cultural backgrounds and from diverse communities.

Graduates are expected to:	Interpersonal Communication Skills	Systems Based Practice	Practice Based Learning and Improvement	Professionalism	Personal/ Professional Development
1. Identify and manage cultural attributes, stereotypes, and biases they bring to clinical encounters.	**ICS-1** (ICS-1) **Communicate effectively with patients, families, and the public, as appropriate, across a broad range of socioeconomic and cultural backgrounds**<br><br>**ICS-2** (ICS-2) **Demonstrate the insight and understanding into emotion and human response to emotion that allow one to appropriately develop and manage human interactions**		**PBLI-1** (PBLI-1) **Identify strengths, deficiencies, and limits in one's knowledge and expertise**	**Prof 2** Show responsiveness to patient needs that supersedes self-interest<br><br>**Prof 5** Demonstrate sensitivity and responsiveness to a diverse patient population, including but not limited to diversity in gender, age, culture, race, religion, disabilities and sexual orientation	**PPD-8** (Prof 6) **Recognize that ambiguity is part of clinical medicine and respond by utilizing appropriate resources in dealing with uncertainty**
2. Integrate into clinical encounters an understanding of diversity (family composition, gender, age, culture, race, religion, disabilities, sexual orientation, and cultural beliefs and practices) by recognizing and respecting families' cultural backgrounds.	**ICS-1** (ICS-1) **Communicate effectively with patients, families, and the public, as appropriate, across a broad range of socioeconomic and cultural backgrounds**<br><br>**ICS-2** (ICS-2) **Demonstrate the insight and understanding into emotion and human response to emotion that allow one to appropriately develop and manage human interactions**	**SBP-2** (SBP-1) **Coordinate patient care within the health system relevant to their clinical specialty**	**PBLI-3** (PBLI-2) **Identify and perform appropriate learning activities to guide personal and professional development**<br><br>**PBLI-8** Develop the necessary skills to be an effective teacher<br><br>**PBLI-9** Participate in the education, of patients, families, students, residents, and other health professionals	**Prof 5** Demonstrate sensitivity and responsiveness to a diverse patient population, including but not limited to diversity in gender, age, culture, race, religion, disabilities and sexual orientation	

CPTI Community Health and Advocacy Milestones Profile

3. Identify children, youth, or families who have limited English language proficiency and demonstrate the ability to use professional interpreters and written materials in the family's primary language to maximize communication	**ICS-1 (ICS-1)**<br>**Communicate effectively with patients, families, and the public, as appropriate, across a broad range of socioeconomic and cultural backgrounds**	**SBP-2 (SBP-1)**<br>**Coordinate patient care within the health system relevant to their clinical specialty**	**PBLI-7**<br>Use information technology to optimize learning and care delivery<br><br>**PBLI-8**<br>Develop the necessary skills to be an effective teacher	**Prof 5**<br>Demonstrate sensitivity and responsiveness to a diverse patient population, including but not limited to diversity in gender, age, culture, race, religion, disabilities and sexual orientation	
4. Identify, analyze, and describe health disparities, as well as organizational assets and barriers to delivering culturally effective services.	**ICS-3**<br>Communicate effectively with physicians, other health professionals, and health related agencies<br><br>**ICS-4**<br>Work effectively as a member or leader of a health care team or other professional group<br><br>**ICS-5**<br>Act in a consultative role to other physicians and health professionals	**SBP-1**<br>Work effectively in various health care delivery settings and systems relevant to their clinical specialty<br><br>**SBP-4 (SBP-2)**<br>**Advocate for quality patient care and optimal care systems**<br><br>**SBP-7**<br>Know how to advocate for the promotion of health and the prevention of disease and injury in populations	**PBLI-4 (PBLI-3)**<br>**Systematically analyze practice using quality improvement methods with the goal of practice improvement**	**Prof 4 (Prof 2)**<br>**Demonstrate a sense of duty and accountability to patients, society and the profession**<br><br>**Prof 5**<br>Demonstrate sensitivity and responsiveness to a diverse patient population, including but not limited to diversity in gender, age, culture, race, religion, disabilities and sexual orientation	

CPTI Community Health and Advocacy Milestones Profile

5. Describe and outline quality improvement activities to achieve health care equity.	**ICS-3**<br>Communicate effectively with physicians, other health professionals, and health related agencies<br><br>**ICS-4**<br>Work effectively as a member or leader of a health care team or other professional group<br><br>**ICS-5**<br>Act in a consultative role to other physicians and health professionals	**SBP-1**<br>Work effectively in various health care delivery settings and systems relevant to their clinical specialty<br><br>**SBP-4 (SBP-2)**<br>**Advocate for quality patient care and optimal care systems**	**PBLI-4 (PBLI-3)**<br>**Systematically analyze practice using quality improvement methods with the goal of practice improvement**	**Prof 5**<br>Demonstrate sensitivity and responsiveness to a diverse patient population, including but not limited to diversity in gender, age, culture, race, religion, disabilities and sexual orientation	

CPTI Community Health and Advocacy Milestones Profile

Goals & Objectives

B. Child Advocacy

Recognizing their unique roles, pediatricians should advocate for the well being of patients, families, and communities. They must develop advocacy skills to address relevant individual, community, and population health issues.

Graduates are expected to:	Interpersonal Communication Skills	Systems Based Practice	Practice Based Learning and Improvement	Professionalism	Personal/ Professional Development
1. Identify and discuss individual, family, and community (local, state and/or national) concerns that impact children's health.	**ICS-1** **(ICS-1)**<br>**Communicate effectively with patients, families, and the public, as appropriate, across a broad range of socioeconomic and cultural backgrounds**<br><br>**ICS-2** **(ICS-2)**<br>**Demonstrate the insight and understanding into emotion and human response to emotion that allow one to appropriately develop and manage human interactions**<br><br>**ICS-3**<br>Communicate effectively with physicians, other health professionals, and health related agencies	**SBP-4** **(SBP-2)**<br>**Advocate for quality patient care and optimal care systems**<br><br>**SBP-7**<br>Know how to advocate for the promotion of health and the prevention of disease and injury in populations	**PBLI-3** **(PBLI-2)**<br>**Identify and perform appropriate learning activities to guide personal and professional development**	**Prof 4** **(Prof 2)**<br>**Demonstrate a sense of duty and accountability to patients, society and the profession**	

CPTI Community Health and Advocacy Milestones Profile

2. Formulate an attainable plan of action in response to a community health need.	**ICS-1 (ICS-1)**<br>**Communicate effectively with patients, families, and the public, as appropriate, across a broad range of socioeconomic and cultural backgrounds**<br><br>**ICS-3**<br>Communicate effectively with physicians, other health professionals, and health related agencies<br><br>**ICS-4**<br>Work effectively as a member or leader of a health care team or other professional group	**SBP-1**<br>Work effectively in various health care delivery settings and systems relevant to their clinical specialty<br><br>**SBP-4 (SBP-2)**<br>**Advocate for quality patient care and optimal care systems**<br><br>**SBP-7**<br>Know how to advocate for the promotion of health and the prevention of disease and injury in populations	**PBLI-2**<br>Set learning and improvement goals<br><br>**PBLI-3 (PBLI-2)**<br>**Identify and perform appropriate learning activities to guide personal and professional development**	**Prof 4 (Prof 2)**<br>**Demonstrate a sense of duty and accountability to patients, society and the profession**	
3. Identify and describe resources to effectively advocate for the well-being of patients, families and communities.	**ICS-1 (ICS-1)**<br>**Communicate effectively with patients, families, and the public, as appropriate, across a broad range of socioeconomic and cultural backgrounds**<br><br>**ICS-3**<br>Communicate effectively with physicians, other health professionals, and health related agencies<br><br>**ICS-4**<br>Work effectively as a member or leader of a health care team or other professional group	**SBP-2 (SBP-1)**<br>**Coordinate patient care within the health system relevant to their clinical specialty**<br><br>**SBP-4 (SBP-2)**<br>**Advocate for quality patient care and optimal care systems**<br><br>**SBP-7**<br>Know how to advocate for the promotion of health and the prevention of disease and injury in populations		**Prof 4 (Prof 2)**<br>**Demonstrate a sense of duty and accountability to patients, society and the profession**	**PPD-6**<br>Provide leadership that enhances team functioning, the learning environment and/or health care system/environment with the ultimate intent of improving care of patients<br><br>**PPD-8 (Prof 6)**<br>**Recognize that ambiguity is part of clinical medicine and respond by utilizing appropriate resources in dealing with uncertainty**

CPTI Community Health and Advocacy Milestones Profile

4. Communicate effectively with community groups and the media.	**ICS-1** **(ICS-1)**<br>**Communicate effectively with patients, families, and the public, as appropriate, across a broad range of socioeconomic and cultural backgrounds**<br><br>**ICS-2** **(ICS-2)**<br>**Demonstrate the insight and understanding into emotion and human response to emotion that allow one to appropriately develop and manage human interactions**<br><br>**ICS-3**<br>Communicate effectively with physicians, other health professionals, and health related agencies<br><br>**ICS-4**<br>Work effectively as a member or leader of a health care team or other professional group		**PBLI-8**<br>Develop the necessary skills to be an effective teacher<br><br>**PBLI-9**<br>Participate in the education, of patients, families, students, residents, and other health professionals	**Prof 4** **(Prof 2)**<br>**Demonstrate a sense of duty and accountability to patients, society and the profession**	
5. Find and use evidence and data to communicate, educate, affect attitude change, and/or obtain funding to achieve specific health outcomes.	**ICS-1** **(ICS-1)**<br>**Communicate effectively with patients, families, and the public, as appropriate, across a broad range of socioeconomic and cultural backgrounds**<br><br>**ICS-3**<br>Communicate effectively with physicians, other health professionals, and health related agencies<br><br>**ICS-4**<br>Work effectively as a member or leader of a health care team or other professional group	**SBP-4** **(SBP-2)**<br>**Advocate for quality patient care and optimal care systems**<br><br>**SBP-7**<br>Know how to advocate for the promotion of health and the prevention of disease and injury in populations	**PBLI-2**<br>Set learning and improvement goals<br><br>**PBLI-6**<br>Locate, appraise, and assimilate evidence form scientific studies related to their patient's health problems	**Prof 4** **(Prof 2)**<br>**Demonstrate a sense of duty and accountability to patients, society and the profession**	

CPTI Community Health and Advocacy Milestones Profile

6. Describe and discuss key features of the legislative process, and identify and communicate with key legislators, community leaders, child advocates, and/or agency administrators about child and family health concerns	**ICS-1 (ICS-1)**<br>**Communicate effectively with patients, families, and the public, as appropriate, across a broad range of socioeconomic and cultural backgrounds**<br><br>**ICS-2 (ICS-2)**<br>**Demonstrate the insight and understanding into emotion and human response to emotion that allow one to appropriately develop and manage human interactions**<br><br>**ICS-3**<br>Communicate effectively with physicians, other health professionals, and health related agencies	**SBP-4 (SBP-2)**<br>**Advocate for quality patient care and optimal care systems**<br><br>**SBP-7**<br>Know how to advocate for the promotion of health and the prevention of disease and injury in populations	**PBLI-8**<br>Develop the necessary skills to be an effective teacher<br><br>**PBLI-9**<br>Participate in the education, of patients, families, students, residents, and other health professionals	**Prof 2**<br>Show responsiveness to patient needs that supersedes self-interest<br><br>**Prof 4 (Prof 2)**<br>**Demonstrate a sense of duty and accountability to patients, society and the profession**	**PPD-6**<br>Provide leadership that enhances team functioning, the learning environment and/or health care system/environment with the ultimate intent of improving care of patients

CPTI Community Health and Advocacy Milestones Profile

Goals & Objectives

C. Medical Home

Pediatricians must be able to identify and/or provide a medical home for all children and families under their care. As defined by the American Academy of Pediatrics, medical home is a model for delivering primary care that is accessible, continuous, comprehensive, family-centered, coordinated, compassionate, and culturally effective.

Graduates are expected to:	Interpersonal Communication Skills	Systems Based Practice	Practice Based Learning and Improvement	Professionalism	Personal/ Professional Development
1. Recognize the family as the principal caregiver and expert in their child's care, the center of strength and support for the child.	**ICS-1 (ICS-1)** **Communicate effectively with patients, families, and the public, as appropriate, across a broad range of socioeconomic and cultural backgrounds**<br><br>**ICS-2 (ICS-2)** **Demonstrate the insight and understanding into emotion and human response to emotion that allow one to appropriately develop and manage human interactions**	**SBP-2 (SBP-1)** **Coordinate patient care within the health system relevant to their clinical specialty**	**PBLI-1 (PBLI-1)** **Identify strengths, deficiencies, and limits in one's knowledge and expertise**<br><br>**PBLI-3 (PBLI-2)** **Identify and perform appropriate learning activities to guide personal and professional development**	**Prof 1 (Prof 1)** **Demonstrate humanism, compassion, integrity, and respect for others based on the characteristics of an empathetic practitioner**<br><br>**Prof 2** Show responsiveness to patient needs that supersedes self-interest	
2. Identify state and national resources such as Medicaid and WIC, as well as relevant state and local programs and resources that support families and child development.	**ICS-3** Communicate effectively with physicians, other health professionals, and health related agencies		**PBLI-1 (PBLI-1)** **Identify strengths, deficiencies, and limits in one's knowledge and expertise**<br><br>**PBLI-10** Take primary responsibility for lifelong learning to improve knowledge, skills, and practice performance through familiarity with general and experience-specific goals and objectives and attendance at conferences	**Prof 4 (Prof 2)** **Demonstrate a sense of duty and accountability to patients, society and the profession**	

CPTI Community Health and Advocacy Milestones Profile

3. Partner with families and youth to access resources (including health care financing), and coordinate care to meet the special needs of patients with acute and chronic conditions, at home and in the school setting.	ICS-1 (ICS-1)<br>**Communicate effectively with patients, families, and the public, as appropriate, across a broad range of socioeconomic and cultural backgrounds**<br><br>ICS-2 (ICS-2)<br>**Demonstrate the insight and understanding into emotion and human response to emotion that allow one to appropriately develop and manage human interactions**<br><br>**ICS-3**<br>Communicate effectively with physicians, other health professionals, and health related agencies<br><br>**ICS-4**<br>Work effectively as a member or leader of a health care team or other professional group<br><br>**ICS-5**<br>Act in a consultative role to other physicians and health professionals	SBP-2 (SBP-1)<br>**Coordinate patient care within the health system relevant to their clinical specialty**<br><br>SBP-5 (SBP-3)<br>**Work in interprofessional teams to enhance patient safety and improve patient care quality**	**PBLI-9**<br>Participate in the education, of patients, families, students, residents, and other health professionals	Prof 4 (Prof 2)<br>**Demonstrate a sense of duty and accountability to patients, society and the profession**	

CPTI Community Health and Advocacy Milestones Profile

4. Collaborate with families and communities to help navigate the health care system, including transition to adult care.	**ICS-1** **(ICS-1)**<br>**Communicate effectively with patients, families, and the public, as appropriate, across a broad range of socioeconomic and cultural backgrounds**<br><br>**ICS-3**<br>Communicate effectively with physicians, other health professionals, and health related agencies<br><br>**ICS-4**<br>Work effectively as a member or leader of a health care team or other professional group<br><br>**ICS-5**<br>Act in a consultative role to other physicians and health professionals	**SBP-1**<br>Work effectively in various health care delivery settings and systems relevant to their clinical specialty<br><br>**SBP-2** **(SBP-1)**<br>**Coordinate patient care within the health system relevant to their clinical specialty**	**PBLI-9**<br>Participate in the education, of patients, families, students, residents, and other health professionals	**Prof 4** **(Prof 2)**<br>**Demonstrate a sense of duty and accountability to patients, society and the profession**	**PPD-6**<br>Provide leadership that enhances team functioning, the learning environment and/or health care system/environment with the ultimate intent of improving care of patients<br><br>**PPD-8** **(Prof 6)**<br>**Recognize that ambiguity is part of clinical medicine and respond by utilizing appropriate resources in dealing with uncertainty**
5. Describe and outline quality improvement activities that result in improved access, coordination, continuity, outcomes of care.	**ICS-3**<br>Communicate effectively with physicians, other health professionals, and health related agencies<br><br>**ICS-4**<br>Work effectively as a member or leader of a health care team or other professional group<br><br>**ICS-5**<br>Act in a consultative role to other physicians and health professionals	**SBP-1**<br>Work effectively in various health care delivery settings and systems relevant to their clinical specialty<br><br>**SBP-4** **(SBP-2)**<br>**Advocate for quality patient care and optimal care systems**<br><br>**SBP-7**<br>Know how to advocate for the promotion of health and the prevention of disease and injury in populations	**PBLI-4** **(PBLI-3)**<br>**Systematically analyze practice using quality improvement methods with the goal of practice improvement**		**PPD-6**<br>Provide leadership that enhances team functioning, the learning environment and/or health care system/environment with the ultimate intent of improving care of patients

6. Identify and access practice tools that support the provision of a medical home, e.g. electronic health records, coding, and accreditation standards (such as NCQA).	**ICS-6**<br>Maintain comprehensive, timely, and legible medical records, if applicable	**SBP-2** **(SBP-1)**<br>**Coordinate patient care within the health system relevant to their clinical specialty**<br><br>**SBP-3**<br>Incorporate considerations of cost awareness and risk-benefit analysis in patient and/or population-based care as appropriate	**PBLI-4** **(PBLI-3)**<br>**Systematically analyze practice using quality improvement methods with the goal of practice improvement**<br><br>**PBLI-7**<br>Use information technology to optimize learning and care delivery		

CPTI Community Health and Advocacy Milestones Profile

Goals & Objectives

D. Special Populations

Pediatricians must be competent in the care of children in special populations, including (but not limited to) children and youth in substitute care, homeless children and youth, children and youth with chronic conditions, immigrants and refugees, and children and youth who are adopted.

Graduates are expected to:	Interpersonal Communication Skills	Systems Based Practice	Practice Based Learning and Improvement	Professionalism	Personal/ Professional Development
1. Identify youth at risk for poor health outcomes and/or with special health care needs; identify the special populations that exist in a community.	**ICS-1 (ICS-1)**<br>**Communicate effectively with patients, families, and the public, as appropriate, across a broad range of socioeconomic and cultural backgrounds**<br><br>**ICS-2 (ICS-2)**<br>**Demonstrate the insight and understanding into emotion and human response to emotion that allow one to appropriately develop and manage human interactions**	**SBP-7**<br>Know how to advocate for the promotion of health and the prevention of disease and injury in populations	**PBLI-1 (PBLI-1)**<br>**Identify strengths, deficiencies, and limits in one's knowledge and expertise**<br><br>**PBLI-2**<br>Set learning and improvement goals<br><br>**PBLI-3 (PBLI-2)**<br>**Identify and perform appropriate learning activities to guide personal and professional development**	**Prof 1 (Prof 1)**<br>**Demonstrate humanism, compassion, integrity, and respect for others based on the characteristics of an empathetic practitioner**<br><br>**Prof 4 (Prof 2)**<br>**Demonstrate a sense of duty and accountability to patients, society and the profession**<br><br>**Prof 5**<br>Demonstrate sensitivity and responsiveness to a diverse patient population, including but not limited to diversity in gender, age, culture, race, religion, disabilities and sexual orientation	

CPTI Community Health and Advocacy Milestones Profile

2. Screen for risks specific to defined special populations.	**ICS-1** **(ICS-1)**<br>**Communicate effectively with patients, families, and the public, as appropriate, across a broad range of socioeconomic and cultural backgrounds**<br><br>ICS-2 **(ICS-2)**<br>**Demonstrate the insight and understanding into emotion and human response to emotion that allow one to appropriately develop and manage human interactions**		**PBLI-6**<br>Locate, appraise, and assimilate evidence form scientific studies related to their patient's health problems	Prof 5<br>Demonstrate sensitivity and responsiveness to a diverse patient population, including but not limited to diversity in gender, age, culture, race, religion, disabilities and sexual orientation	
3. Demonstrate a working knowledge of psychosocial issues, legal protections, policies, and services provided for these populations at the local, state, and federal levels.	**ICS-2** **(ICS-2)**<br>**Demonstrate the insight and understanding into emotion and human response to emotion that allow one to appropriately develop and manage human interactions**<br><br>**ICS-4**<br>Work effectively as a member or leader of a health care team or other professional group	**SBP-1**<br>Work effectively in various health care delivery settings and systems relevant to their clinical specialty	**PBLI-1** **(PBLI-1)**<br>**Identify strengths, deficiencies, and limits in one's knowledge and expertise**<br><br>**PBLI-3** **(PBLI-2)**<br>**Identify and perform appropriate learning activities to guide personal and professional development**	**Prof 4** **(Prof 2)**<br>**Demonstrate a sense of duty and accountability to patients, society and the profession**<br><br>Prof 5<br>Demonstrate sensitivity and responsiveness to a diverse patient population, including but not limited to diversity in gender, age, culture, race, religion, disabilities and sexual orientation	

CPTI Community Health and Advocacy Milestones Profile

Goals & Objectives

E. Pediatrician as Consultant/Collaborative Leader/Partner

Pediatricians must be able to act as child health consultants in their communities. Using collaborative skills, they must be able to work with multidisciplinary teams, community members, educators, and representatives from community organizations and legislative bodies.

Graduates are expected to:	Interpersonal Communication Skills	Systems Based Practice	Practice Based Learning and Improvement	Professionalism	Personal/ Professional Development
1. Identify potential opportunities to serve as a health consultant in the community where he/she practices pediatrics and demonstrate the ability to communicate effectively with a variety of audiences within that community.	**ICS-1 (ICS-1)**<br>**Communicate effectively with patients, families, and the public, as appropriate, across a broad range of socioeconomic and cultural backgrounds**<br><br>**ICS-3**<br>Communicate effectively with physicians, other health professionals, and health related agencies<br><br>**ICS-4**<br>Work effectively as a member or leader of a health care team or other professional group<br><br>**ICS-5**<br>Act in a consultative role to other physicians and health professionals	**SBP-7**<br>Know how to advocate for the promotion of health and the prevention of disease and injury in populations	**PBLI-1 (PBLI-1)**<br>**Identify strengths, deficiencies, and limits in one's knowledge and expertise**<br><br>**PBLI-9**<br>Participate in the education, of patients, families, students, residents, and other health professionals	**Prof 4 (Prof 2)**<br>**Demonstrate a sense of duty and accountability to patients, society and the profession**	**PPD-6**<br>Provide leadership that enhances team functioning, the learning environment and/or health care system/environment with the ultimate intent of improving care of patients

2. Describe and discuss the essential qualities of community partnerships including shared vision, the use of complementary strengths, the willingness to collaborate, and the development of agreed-on boundaries.	**ICS-1 (ICS-1)**<br>**Communicate effectively with patients, families, and the public, as appropriate, across a broad range of socioeconomic and cultural backgrounds**<br><br>**ICS-2 (ICS-2)**<br>**Demonstrate the insight and understanding into emotion and human response to emotion that allow one to appropriately develop and manage human interactions**<br><br>**ICS-3**<br>Communicate effectively with physicians, other health professionals, and health related agencies<br><br>**ICS-4**<br>Work effectively as a member or leader of a health care team or other professional group		**PBLI-3 (PBLI-2)**<br>**Identify and perform appropriate learning activities to guide personal and professional development**<br><br>Prof 5<br>Demonstrate sensitivity and responsiveness to a diverse patient population, including but not limited to diversity in gender, age, culture, race, religion, disabilities and sexual orientation<br><br>**PBLI-9**<br>Participate in the education, of patients, families, students, residents, and other health professionals	**Prof 1 (Prof 1)**<br>**Demonstrate humanism, compassion, integrity, and respect for others based on the characteristics of an empathetic practitioner**<br><br>**Prof 4 (Prof 2)**<br>**Demonstrate a sense of duty and accountability to patients, society and the profession**	**PPD-6**<br>Provide leadership that enhances team functioning, the learning environment and/or health care system/environment with the ultimate intent of improving care of patients

CPTI Community Health and Advocacy Milestones Profile

3. Define and discuss principles of consensus building, including fostering inclusiveness, identifying mutual goals, setting measurable outcomes, using effective problem-solving strategies and negotiating towards consensus	**ICS-1 (ICS-1)** **Communicate effectively with patients, families, and the public, as appropriate, across a broad range of socioeconomic and cultural backgrounds**<br><br>**ICS-2 (ICS-2)** **Demonstrate the insight and understanding into emotion and human response to emotion that allow one to appropriately develop and manage human interactions**<br><br>**ICS-3** Communicate effectively with physicians, other health professionals, and health related agencies<br><br>**ICS-4** Work effectively as a member or leader of a health care team or other professional group		**PBLI-2** Set learning and improvement goals<br><br>**PBLI-3 (PBLI-2)** **Identify and perform appropriate learning activities to guide personal and professional development**	**Prof 1 (Prof 1)** **Demonstrate humanism, compassion, integrity, and respect for others based on the characteristics of an empathetic practitioner**<br><br>**Prof 4 (Prof 2)** **Demonstrate a sense of duty and accountability to patients, society and the profession**	**PPD-6** Provide leadership that enhances team functioning, the learning environment and/or health care system/environment with the ultimate intent of improving care of patients

CPTI Community Health and Advocacy Milestones Profile

<table>
<tr><td colspan="6">Goals & Objectives
F. Educational and Child Care Settings
Pediatricians must be able to interact with staff in schools and child care settings to improve the health and educational environments for children.</td></tr>
<tr><th>Graduates are expected to:</th><th>Interpersonal Communication Skills</th><th>Systems Based Practice</th><th>Practice Based Learning and Improvement</th><th>Professionalism</th><th>Personal/ Professional Development</th></tr>
<tr><td>1. Promote the children’s health and success in school by assessing children for school readiness, making appropriate referrals to relevant community services, and communicating and collaborating with school nurses, teachers and administration.</td><td>ICS-1 (ICS-1)
Communicate effectively with patients, families, and the public, as appropriate, across a broad range of socioeconomic and cultural backgrounds

ICS-3
Communicate effectively with physicians, other health professionals, and health related agencies

ICS-4
Work effectively as a member or leader of a health care team or other professional group</td><td>SBP-1
Work effectively in various health care delivery settings and systems relevant to their clinical specialty

SBP-5 (SBP-3)
Work in interprofessional teams to enhance patient safety and improve patient care quality

SBP-7
Know how to advocate for the promotion of health and the prevention of disease and injury in populations</td><td>PBLI-9
Participate in the education, of patients, families, students, residents, and other health professionals</td><td></td><td>PPD-6
Provide leadership that enhances team functioning, the learning environment and/or health care system/environment with the ultimate intent of improving care of patients</td></tr>
</table>

2. Explain how to work with families, educational, and child care institutions to help provide optimal learning environments for all children. This includes knowledge about high quality early education, the Individuals with Disabilities Education Act, participation in Individualized Education Plans and Individual Family Service Plans, and provision of medications and/or medical care in school settings.	**ICS-1 (ICS-1)**<br>**Communicate effectively with patients, families, and the public, as appropriate, across a broad range of socioeconomic and cultural backgrounds**<br><br>**ICS-3**<br>Communicate effectively with physicians, other health professionals, and health related agencies<br><br>**ICS-4**<br>Work effectively as a member or leader of a health care team or other professional group	**SBP-2 (SBP-1)**<br>**Coordinate patient care within the health system relevant to their clinical specialty**<br><br>**SBP-5 (SBP-3)**<br>**Work in interprofessional teams to enhance patient safety and improve patient care quality**	**PBLI-1 (PBLI-1)**<br>**Identify strengths, deficiencies, and limits in one's knowledge and expertise**<br><br>**PBLI-9**<br>Participate in the education, of patients, families, students, residents, and other health professionals	**Prof 4 (Prof 2)**<br>**Demonstrate a sense of duty and accountability to patients, society and the profession**	**PPD-6**<br>Provide leadership that enhances team functioning, the learning environment and/or health care system/environm ent with the ultimate intent of improving care of patients
3. Describe and discuss how a physician can collaborate to improve the physical, social, and health environment in schools and child care settings.	**ICS-1 (ICS-1)**<br>**Communicate effectively with patients, families, and the public, as appropriate, across a broad range of socioeconomic and cultural backgrounds**<br><br>**ICS-2 (ICS-2)**<br>**Demonstrate the insight and understanding into emotion and human response to emotion that allow one to appropriately develop and manage human interactions**<br><br>**ICS-3**<br>Communicate effectively with phys-icians, other health professionals, and health related agencies<br><br>**ICS-4**<br>Work effectively as a member or leader of a health care team or other	**SBP-1**<br>Work effectively in various health care delivery settings and systems relevant to their clinical specialty<br><br>**SBP-4 (SBP-2)**<br>**Advocate for quality patient care and optimal care systems**<br><br>**SBP-5 (SBP-3)**<br>**Work in interprofessional teams to enhance patient safety and improve patient care quality**<br><br>**SBP-7**<br>Know how to advocate for the promotion of health and the prevention of disease and injury in populations	**PBLI-3 (PBLI-2)**<br>**Identify and perform appropriate learning activities to guide personal and professional development**	**Prof 4 (Prof 2)**<br>**Demonstrate a sense of duty and accountability to patients, society and the profession**	**PPD-6**<br>Provide leadership that enhances team functioning, the learning environment and/or health care system/environm ent with the ultimate intent of improving care of patients

	professional group				

Goals & Objectives

G. Public Health and Prevention

Pediatricians must be able to practice from a population–based perspective and understand relationships between individual, family, and community-level health determinants that affect children and families in the communities they serve. Pediatricians must be able to apply community assets and resources to prevent illness, injury, and death.

Graduates are expected to:	Interpersonal Communication Skills	Systems Based Practice	Practice Based Learning and Improvement	Professionalism	Personal/ Professional Development
1. Describe and discuss modifiable risk factors and the evolving epidemiology of pediatric illnesses and their impact on child health and well-being and child health equity.	**ICS-3** Communicate effectively with physicians, other health professionals, and health related agencies<br><br>**ICS-4** Work effectively as a member or leader of a health care team or other professional group	**SBP-7** Know how to advocate for the promotion of health and the prevention of disease and injury in populations	**PBLI-1 (PBLI-1) Identify strengths, deficiencies, and limits in one's knowledge and expertise**<br><br>**PBLI-3 (PBLI-2) Identify and perform appropriate learning activities to guide personal and professional development**	**Prof 4 (Prof 2) Demonstrate a sense of duty and accountability to patients, society and the profession**	

CPTI Community Health and Advocacy Milestones Profile

2. Identify and discuss child health issues at the national, state, and local levels by accessing and using vital statistics, surveillance data, community asset mapping, and other sources of data.	**ICS-3**<br>Communicate effectively with physicians, other health professionals, and health related agencies<br><br>**ICS-4**<br>Work effectively as a member or leader of a health care team or other professional group	**SBP-7**<br>Know how to advocate for the promotion of health and the prevention of disease and injury in populations	**PBLI-6**<br>Locate, appraise, and assimilate evidence form scientific studies related to their patient's health problems	**Prof 4 (Prof 2)**<br>**Demonstrate a sense of duty and accountability to patients, society and the profession**	

3. Identify measurable outcomes for assessing progress in addressing child health issues, including health equity.	**ICS-3**<br>Communicate effectively with physicians, other health professionals, and health related agencies<br><br>**ICS-4**<br>Work effectively as a member or leader of a health care team or other professional group		**PBLI-2**<br>Set learning and improvement goals<br><br>**PBLI-4 (PBLI-3)**<br>**Systematically analyze practice using quality improvement methods with the goal of practice improvement**		
4. Identify and describe effective public health interventions at the individual, community, and national level, e.g. screening & prevention programs aimed at modifying risk factors for disease or adverse health outcomes, and case identification and tracking.	**ICS-3**<br>Communicate effectively with physicians, other health professionals, and health related agencies<br><br>**ICS-4**<br>Work effectively as a member or leader of a health care team or other professional group	**SBP-7**<br>Know how to advocate for the promotion of health and the prevention of disease and injury in populations	**PBLI-1 (PBLI-1)**<br>**Identify strengths, deficiencies, and limits in one's knowledge and expertise**<br><br>**PBLI-4 (PBLI-3)**<br>**Systematically analyze practice using quality improvement methods with the goal of practice improvement**	**Prof 4 (Prof 2)**<br>**Demonstrate a sense of duty and accountability to patients, society and the profession**	**PPD-6**<br>Provide leadership that enhances team functioning, the learning environment and/or health care system/environment with the ultimate intent of improving care of patients

5. Describe and discuss the individual practitioner's role within the greater public health infrastructure, including early identification, notification, mandated reporting, and emergency planning/response recovery	**ICS-3**<br>Communicate effectively with physicians, other health professionals, and health related agencies<br><br>**ICS-4**<br>Work effectively as a member or leader of a health care team or other professional group	**SBP-5** **(SBP-3)**<br>**Work in interprofessional teams to enhance patient safety and improve patient care quality**<br><br>**SBP-7**<br>Know how to advocate for the promotion of health and the prevention of disease and injury in populations	**PBLI-8**<br>Develop the necessary skills to be an effective teacher<br><br>**PBLI-9**<br>Participate in the education, of patients, families, students, residents, and other health professionals	**Prof 1** **(Prof 1)**<br>**Demonstrate humanism, compassion, integrity, and respect for others based on the characteristics of an empathetic practitioner**<br><br>**Prof 2**<br>Show responsiveness to patient needs that supersedes self-interest<br><br>**Prof 4** **(Prof 2)**<br>**Demonstrate a sense of duty and accountability to patients, society and the profession**	**PPD-6**<br>Provide leadership that enhances team functioning, the learning environment and/or health care system/environment with the ultimate intent of improving care of patients

Goals & Objectives

H. Inquiry and Application

Pediatricians should be capable of pursuing inquiry that advances the health of children, families, and communities.

Graduates are expected to:	Interpersonal Communication Skills	Systems Based Practice	Practice Based Learning and Improvement	Professionalism	Personal/ Professional Development
1. Assess and apply evidence-based practices for children and families relevant to the needs and resources of their communities.	**ICS-4**<br>Work effectively as a member or leader of a health care team or other professional group	**SBP-7**<br>Know how to advocate for the promotion of health and the prevention of disease and injury in populations	**PBLI-3** **(PBLI-2)**<br>**Identify and perform appropriate learning activities to guide personal and professional development**<br><br>**PBLI-6**<br>Locate, appraise, and assimilate evidence form scientific studies related to their patient's health problems		

2. Discuss how quality improvement assessments and methodology can be integrated into interactions with community organizations serving children and families.	ICS-1 **(ICS-1)**<br>**Communicate effectively with patients, families, and the public, as appropriate, across a broad range of socioeconomic and cultural backgrounds**<br><br>**ICS-4**<br>Work effectively as a member or leader of a health care team or other professional group	SBP-4 **(SBP-2)**<br>**Advocate for quality patient care and optimal care systems**	PBLI-4 **(PBLI-3)**<br>**Systematically analyze practice using quality improvement methods with the goal of practice improvement**		
3. Describe and discuss the ethical issues that relate to research and scholarship in communities.	ICS-2 **(ICS-2)**<br>**Demonstrate the insight and understanding into emotion and human response to emotion that allow one to appropriately develop and manage human interactions**<br><br>**ICS-4**<br>Work effectively as a member or leader of a health care team or other professional group		PBLI-3 **(PBLI-2)**<br>**Identify and perform appropriate learning activities to guide personal and professional development**		

4. Describe and discuss different methodologies of research in communities, including community-based participatory research.	**ICS-4**<br>Work effectively as a member or leader of a health care team or other professional group		**PBLI-1** **(PBLI-1)**<br>**Identify strengths, deficiencies, and limits in one's knowledge and expertise**<br><br>**PBLI-4** **(PBLI-3)**<br>**Systematically analyze practice using quality improvement methods with the goal of practice improvement**<br><br>**PBLI-6**<br>Locate, appraise, and assimilate evidence form scientific studies related to their patient's health problems		
