# Supplementary material for: The Community Pediatrics Training Initiative Project Planning Tool: A Practical Approach to Community-Based Advocacy
Source: MedEdPORTAL. 2017 Sep 18;13:10630. doi: 10.15766/mep_2374-8265.10630 (PMC6338167; doi:10.15766/mep_2374-8265.10630)
Supplement: Supplementary file 1 — A. CHAMP.pdf B. CHAMP Mapping Tool.pdf C. AAP CPTI Project Planning Tool.docx D. AAP CPTI Project Planning Tool Milestones-Based Assessment Rubric.docx E. Project Planning Tool Users Guide.pptx [file mep-13-10630-s001.zip › B. CHAMP Mapping Tool.pdf]

CHAMP Mapping Tool

AAP CPTI Community Health and Advocacy Milestones Mapping Tool

- All competencies listed in the column "Reporting Currently Required" are numbered according with the 2013 document listing the 21 Pediatric Milestones to be evaluated as of 2014. It can be found at:
  - http://acgme.org/acgmeweb/Portals/0/PDFs/Milestones/PediatricsMilestones.pdf
- All competencies listed in the column "Reporting Not Yet Required" are numbered according with the original 2012 Milestones document listing all 51 Pediatric Milestones. That document can be found at:
  - https://www.abp.org/abpwebsite/publicat/milestones.pdf
- Any questions regarding the numbering should be addressed to the lead author, Benjamin Hoffman at hoffmanb@ohsu.edu

Community Health and Advocacy Milestones Profile (CHAMP) Mapping Tool

Community Health and Advocacy Goals & Objectives					
**A. Culturally Effective Care**<br><br>Pediatricians must demonstrate skills that result in effective care of children and families from all cultural backgrounds and from diverse communities.<br><br>**Graduates are expected to:**	**Milestones-Based Competencies**<br>As of September 2014<br>Reporting Currently Required	Reporting Not Yet Required	**Rotation/ Curricular Activity**	**Assessment Method/ Demonstration of Competence**	**Level of Competence to be Demonstrated**<br>**-Knows**<br>**-Knows how**<br>**-Shows how**<br>**-Does**
1. Identify and manage cultural attributes, stereotypes, and biases they bring to clinical encounters	ICS1<br>ICS2<br>PBLI1<br>Prof6	Prof2<br>Prof5			
2. Integrate into clinical encounters an understanding of diversity (e.g. family composition, gender, age, culture, race, religion, disabilities, sexual orientation, and cultural beliefs and practices) by recognizing and respecting families' cultural backgrounds.	ICS1<br>ICS2<br>SBP1<br>PBLI2	PBLI8<br>PBLI9<br>Prof5			
3. Identify children, youth, or families who have limited English language Proficiency and demonstrate the ability to use Professional interpreters and written materials in the family's primary language to maximize communication.	ICS1<br>SBP1	PBLI7<br>PBLI8<br>Prof5			
4. Identify, analyze, and describe health disparities, as well as organizational assets and barriers to delivering culturally effective services.	SBP2<br>PBLI3<br>Prof2	ICS3<br>ICS4<br>ICS5<br>SBP1<br>Prof5			
5. Describe and outline quality improvement activities to achieve health care equity.	SBP2<br>PBLI3	ICS3<br>ICS4<br>ICS5<br>Prof5			

Community Health and Advocacy Milestones Profile (CHAMP) Mapping Tool

Community Health and Advocacy Goals & Objectives					
**B. Child Advocacy**<br><br>Recognizing their unique roles, pediatricians should advocate for the well-being of patients, families, and communities. They must develop advocacy skills to address relevant individual, community, and population health issues.<br><br>**Graduates are expected to:**	**Milestones-Based Competencies**<br>As of September 2014<br>Reporting Currently Required	**Milestones-Based Competencies**<br>As of September 2014<br>Reporting Not Yet Required	**Rotation/ Curricular Activity**	**Assessment Method/ Demonstration of Competence**	**Level of Competence to be Demonstrated**<br>**-Knows**<br>**-Knows how**<br>**-Shows how**<br>**-Does**
1. Identify and discuss individual, family, and community (local, state and/or national) concerns that impact children's health.	ICS2<br>SBP2<br>PBLI2<br>Prof2	ICS1<br>ICS3<br>SBP7			
2. Formulate an attainable plan of action in response to a community health need.	ICS1<br>SBP2<br>PBLI2<br>Prof2	ICS3<br>ICS4<br>SBP1<br>SBP7<br>PBLI2			
3. Identify and describe resources to effectively advocate for the well-being of patients, families, and communities.	ICS1<br>SBP1<br>SBP2<br>Prof4<br>Prof6	ICS3<br>SBP7<br>PPD6			
4. Communicate effectively with community groups and the media.	ICS1<br>ICS2<br>Prof2	ICS3<br>ICS4<br>PBLI8<br>PBLI9			
5. Find and use evidence and data to communicate, educate, affect attitude change, and/or obtain funding to achieve specific health outcomes.	ICS1<br>SBP2<br>Prof2	ICS3<br>ICS4<br>SBP7<br>PBLI8<br>PBLI9<br>Prof2<br>PPD6			
6. Describe and discuss key features of the legislative process, and identify and communicate with key legislators, community leaders, child advocates, and/or agency administrators about child and family health concerns.	ICS1<br>ICS2<br>SBP2<br>Prof2	ICS3<br>Prof2<br>SBP7<br>PBLI8<br>PBLI9<br>PPD6			

Community Health and Advocacy Milestones Profile (CHAMP) Mapping Tool

COMMUNITY PEDIATRICS TRAINING INITIATIVE
A program of the American Academy of Pediatrics

Community Health and Advocacy Goals & Objectives					
**C. Medical Home**<br><br>Pediatricians must be able to identify and/or provide a medical home for all children and families under their care. As defined by the American Academy of Pediatrics, medical home is a model for delivering primary care that is accessible, continuous, comprehensive, family-centered, coordinated, compassionate, and culturally effective.<br><br>**Graduates are expected to:**	**Milestones-Based Competencies**<br>As of September 2014<br>Reporting Currently Required	**Milestones-Based Competencies**<br>As of September 2014<br>Reporting Not Yet Required	**Rotation/ Curricular Activity**	**Assessment Method/ Demonstration of Competence**	**Level of Competence to be Demonstrated**<br><br>**-Knows**<br>**-Knows how**<br>**-Shows how**<br>**-Does**
1. Recognize the family as the principal caregiver and expert in their child's care, the center of strength and support for the child.	ICS1<br>ICS2<br>SBP2<br>PBLI1<br>PBLI2<br>Prof1	Prof2			
2. Identify state and national resources such as Medicaid and WIC, as well as relevant state and local programs and resources that support families and child development.	Prof2	ICS3<br>PBLI1<br>PBLI10			
3. Partner with families and youth to access resources (including health care financing), and coordinate care to meet the special needs of patients with acute and chronic conditions, at home and in the school setting.	ICS1<br>ICS2<br>SBP1<br>SBP3<br>Prof2	ICS3 ICS4<br>ICS5<br>PBLI9			
4. Collaborate with families and communities to help navigate the health care system, including transition to adult care.	ICS1<br>SBP1<br>Prof2<br>Prof6	ICS3 ICS4<br>ICS5<br>SBP1<br>PBLI9<br>PPD6			
5. Describe and outline quality improvement activities that result in improved access, coordination, continuity, and outcomes of care.	SBP2<br>PBLI3	ICS3 ICS4<br>ICS5<br>SBP1<br>SBP7<br>PPD6			
6. Identify and access practice tools that support the provision of a medical home, e.g. electronic health records, coding, and accreditation standards (such as NCQA).	SBP1<br>PBLI3	ICS6<br>SBP3<br>PBLI7			

COMMUNITY PEDIATRICS TRAINING INITIATIVE
A program of the American Academy of Pediatrics

Community Health and Advocacy Milestones Profile (CHAMP) Mapping Tool

Community Health and Advocacy Goals & Objectives					
**D. Special Populations**<br><br>Pediatricians must be competent in the care of children in special populations, including (but not limited to) children and youth in substitute care, homeless children and youth, children and youth with chronic conditions, immigrants and refugees, and children and youth who are adopted.<br><br>**Graduates are expected to:**	**Milestones-Based Competencies**<br>As of September 2014<br>Reporting Currently Required	 <br>Reporting Not Yet Required	**Rotation/ Curricular Activity**	**Assessment Method/ Demonstration of Competence**	**Level of Competence to be Demonstrated**<br>**-Knows**<br>**-Knows how**<br>**-Shows how**<br>**-Does**
1. Identify youth at risk for poor health outcomes and/or with special health care needs; identify the special populations that exist in a community.	ICS1<br>ICS2<br>PBLI1<br>PBLI2<br>Prof1<br>Prof2	PBLI2<br>Prof5<br>SBP7			
2. Screen for risks specific to defined special populations.	ICS1<br>ICS2	PBLI6<br>Prof5			
3. Demonstrate a working knowledge of psychosocial issues, legal protections, policies, and services provided for these populations at the local, state, and federal levels.	ICS2<br>PBLI1<br>PBLI2<br>Prof2	ICS4<br>SBP1<br>Prof5			

Community Health and Advocacy Milestones Profile (CHAMP) Mapping Tool

Community Health and Advocacy Goals & Objectives					
**E. Pediatrician as a Consultant/Collaborative Leader/Partner**<br><br>Pediatricians must be able to act as child health consultants in their communities. Using collaborative skills, they must be able to work with multidisciplinary teams, community members, educators, and representatives from community organizations and legislative bodies.<br><br>**Graduates are expected to:**	**Milestones-Based Competencies**<br>As of September 2014<br>Reporting Currently Required	**Milestones-Based Competencies**<br>As of September 2014<br>Reporting Not Yet Required	**Rotation/ Curricular Activity**	**Assessment Method/ Demonstration of Competence**	**Level of Competence to be Demonstrated**<br>**-Knows**<br>**-Knows how**<br>**-Shows how**<br>**-Does**
1. Identify potential opportunities to serve as a health consultant in the community where he/she practices pediatrics and demonstrate the ability to communicate effectively with a variety of audiences within that community.	ICS1<br>PBLI1<br>Prof2	ICS3<br>ICS4<br>ICS5<br>SBP7<br>PBLI9<br>PPD6			
2. Describe and discuss the essential qualities of community partnerships including shared vision, the use of complementary strengths, the willingness to collaborate, and the development of agreed-on boundaries.	ICS1<br>PBLI2<br>Prof1<br>Prof2	ICS2<br>ICS3<br>ICS4<br>PBLI5<br>PBLI9<br>PPD6			
3. Define and discuss principles of consensus building, including fostering inclusiveness, identifying mutual goals, setting measurable outcomes, using effective problem-solving strategies, and negotiating towards consensus.	ICS1<br>ICS2<br>PBLI2<br>Prof1<br>Prof2	ICS3<br>ICS4<br>PBLI2<br>PPD6			

Community Health and Advocacy Milestones Profile (CHAMP) Mapping Tool

Community Health and Advocacy Goals & Objectives					
**F. Educational and Child Care Settings**<br><br>Pediatricians must be able to interact with staff in schools and child care settings to improve the health and educational environments for children.<br><br>**Graduates are expected to:**	**Milestones-Based Competencies**<br>As of September 2014<br>Reporting Currently Required	Reporting Not Yet Required	**Rotation/ Curricular Activity**	**Assessment Method/ Demonstration of Competence**	**Level of Competence to be Demonstrated**<br>**-Knows**<br>**-Knows how**<br>**-Shows how**<br>**-Does**
1. Promote the children's health and success in school by assessing children for school readiness, making appropriate referrals to relevant community services, and communicating and collaborating with school nurses, teachers, and administration.	ICS1<br>SBP2	ICS3<br>ICS4<br>SBP1<br>PBLI9<br>PPD6<br>SBP7			
2. Explain how to work with families, educational, and child care institutions to help provide optimal learning environments for all children. This includes knowledge about high quality early education, the Individuals with Disabilities Education Act (IDEA), participation in Individualized Education Plans (IEP) and Individual Family Service Plans (IFSP), and provision of medications and/or medical care in school settings.	ICS1<br>SBP1<br>SBP3<br>PBLI1<br>Prof2	ICS3<br>ICS4<br>PBLI9<br>PPD6			
3. Describe and discuss how a physician can collaborate to improve the physical, social, and health environment in schools and child care settings.	ICS1<br>ICS2<br>SBP2<br>SBP3<br>PBLI2<br>Prof2	ICS3<br>ICS4<br>SBP1<br>PPD6<br>SBP7			

COMMUNITY PEDIATRICS TRAINING INITIATIVE
A program of the American Academy of Pediatrics

Community Health and Advocacy Milestones Profile (CHAMP) Mapping Tool

Community Health and Advocacy Goals & Objectives					
**G. Public Health and Prevention**<br><br>Pediatricians must be able to practice from a population–based perspective and understand relationships between individual, family, and community-level health determinants that affect children and families in the communities they serve. Pediatricians must be able to apply community assets and resources to prevent illness, injury, and death.<br><br>**Graduates are expected to:**	**Milestones-Based Competencies**<br>As of September 2014<br>Reporting Currently Required	 <br>Reporting Not Yet Required	**Rotation/ Curricular Activity**	**Assessment Method/ Demonstration of Competence**	**Level of Competence to be Demonstrated**<br>**-Knows**<br>**-Knows how**<br>**-Shows how**<br>**-Does**
1. Describe and discuss modifiable risk factors and the evolving epidemiology of pediatric illnesses and their impact on child health and well-being and child health equity.	PBLI1<br>PBLI2<br>Prof2	ICS3<br>ICS4<br>SBP7			
2. Identify and discuss child health issues at the national, state, and local levels by accessing and using vital statistics, surveillance data, community asset mapping, and other sources of data.	Prof2	ICS3<br>ICS4<br>SBP7<br>PBLI6			
3. Identify measurable outcomes for assessing progress in addressing child health issues, including health equity.	PBLI3	ICS3<br>ICS4<br>PLBI2			
4. Identify and describe effective public health interventions at the individual, community, and national level, e.g. screening & prevention programs aimed at modifying risk factors for disease or adverse health outcomes, and case identification and tracking.	PBLI1<br>PBLI3<br>Prof2	ICS3<br>ICS4<br>SBP7<br>PPD6			
5. Describe and discuss the individual practitioner's role within the greater public health infrastructure, including early identification, notification, mandated reporting, and emergency planning/response recovery.	SBP3<br>Prof1<br>Prof2	ICS3<br>ICS4<br>PBLI8<br>PBLI9<br>Prof2<br>PPD6			

COMMUNITY PEDIATRICS TRAINING INITIATIVE
A program of the American Academy of Pediatrics

Community Health and Advocacy Milestones Profile (CHAMP) Mapping Tool

Community Health and Advocacy Goals & Objectives					
**H. Inquiry and Application**<br><br>Pediatricians should be capable of pursuing inquiry that advances the health of children, families, and communities.<br><br>**Graduates are expected to:**	**Milestones-Based Competencies**<br>As of September 2014<br>Reporting Currently Required	Reporting Not Yet Required	**Rotation/ Curricular Activity**	**Assessment Method/ Demonstration of Competence**	**Level of Competence to be Demonstrated**<br>**-Knows**<br>**-Knows how**<br>**-Shows how**<br>**-Does**
1. Assess and apply evidence-based practices for children and families relevant to the needs and resources of their communities.	PBLI2	ICS4<br>SBP7<br>PBLI6			
2. Discuss how quality improvement assessments and methodology can be integrated into interactions with community organizations serving children and families.	ICS1<br>SBP2<br>PBLI3	ICS4			
3. Describe and discuss the ethical issues that relate to research and scholarship in communities.	ICS2<br>PBLI2	ICS4			
4. Describe and discuss different methodologies of research in communities, including community-based participatory research.	PBLI1<br>PBLI3	ICS4<br>PBLI6			
