# Supplementary material for: The Community Pediatrics Training Initiative Project Planning Tool: A Practical Approach to Community-Based Advocacy
Source: MedEdPORTAL. 2017 Sep 18;13:10630. doi: 10.15766/mep_2374-8265.10630 (PMC6338167; doi:10.15766/mep_2374-8265.10630)
Supplement: Supplementary file 1 — A. CHAMP.pdf B. CHAMP Mapping Tool.pdf C. AAP CPTI Project Planning Tool.docx D. AAP CPTI Project Planning Tool Milestones-Based Assessment Rubric.docx E. Project Planning Tool Users Guide.pptx [file mep-13-10630-s001.zip › C. AAP CPTI Project Planning Tool.docx]

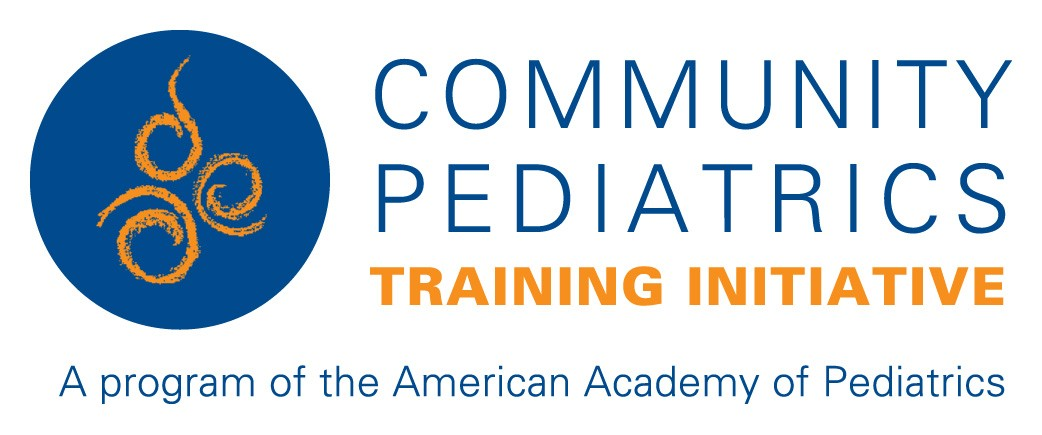


**Project Planning Tool:**

**Developing a Community Advocacy Project Proposal**

Advocacy is largely driven by passion related to solving a specific problem or working to ensure the voice of a specific population is heard. Through this activity, you will learn the basic process for working to effect change in a community. However, true passion related to a given field, advocacy issue, or population cannot be forced . . . it must come from within.

While some residents discover their passion during the course of their training (or even prior to becoming physicians) and progress to developing and implementing an advocacy program or project, many do not. If you fall into the latter group, *don’t worry* - the purpose of this activity is to learn the steps for defining an advocacy interest, and the process of designing a project to address the problem, so that you are prepared to advocate effectively whenever the population or problem finds you. This concept is very similar to preparing to effectively lead an actual resuscitation. While “it happens when it happens,” you can become better prepared for your role by participating in mock codes in a simulation lab.

Your tasks for this activity are:

- To define an advocacy issue that you wish to explore
- Build expertise by thinking through the process
- And practice the skills necessary to build collaborative partnerships with a community to advocate for kids.

While your work on this activity may end there, just like a mock code concludes in the simulation lab, you may use these skills to implement your ideas, apply for funding, or move on to a different idea. The overall goal of this activity is to equip you with the skills and preparation that you will need to act, when the right problem finds you at the right time.

**Learning Objectives for this Project Planning Tool:**

Upon completion of this exercise, you should be able to:

- Identify an area of interest related to child health and well-being.
- Locate population-level data and conduct a literature review to develop expertise about a particular advocacy topic.
- Identify key stakeholders and note key shared values and goals regarding a particular issue.
- Define three measurable objectives as they relate to your advocacy topic.
- Develop a plan to impact a community that will ideally lead to a change in your measurable

objectives.

- Describe a sample PDSA cycle as it relates to a particular advocacy issue.
- Describe your intended role in advocacy as a part of your professional role as a pediatrician.

So, where do you start? Many effective advocates will tell you that **the problem or the population finds you!**

There are 2 methods that can be very useful as you identify and narrow your focus for an advocacy project:

1. **Choose an issue about which you would like to build expertise and for which you would like to advocate.**

This issue might be one for which you have explored in the past, one by which you have personally been affected, something about which you simply wanted to learn more, something that you have questioned, or something that has impacted a patient and/or population for whom you have provided care. Once you have selected your issue, your task will involve building expertise related to the issue, and identifying potential community partners who can help you in developing and implementing an advocacy project related to your chosen issue. Some examples of advocacy issues that one might choose include promoting car seat safety among patients, preventing childhood obesity in a certain community, preventing gun violence, promoting programs to support early brain development, or increasing access to healthy food within a community. Ask yourself, “What issue(s) am I truly passionate about?” Identify the need and/or community in need of our advocacy, collaborate to develop assets, and change the world!

1. **Focus on a population for which you desire to advocate**.

For example, you may have an interest in working with undocumented Mexican immigrants, Syrian refugees, Native American populations, or children affected by a certain disease (such as cystic fibrosis or sickle cell disease). If you choose this approach, your task will be to engage the specific community and partner with them to identify their needs, address their concerns, and serve as an advocate on their behalf.

Whatever issue or population you choose, it is important to keep your focus specific and clearly defined.

Your advocacy proposal can involve any level of advocacy, from the individual-level (helping a child with limited financial resources obtain a mobility device), to the community level (developing an obesity prevention program within a specific neighborhood), to broader policy-level advocacy (working with a city councilor or even a state legislator to introduce a bicycle helmet law).

In developing a community advocacy project, it can be very helpful to consider the following 10 steps, which are strategically oriented to help guide you through the project development process.

**10 Steps for Community Advocacy**

1. [Identify the problem](#Step1) 7. [Ensure things are done WITH the community,](#Step7)

2. [Define the baseline](#Step2) [not TO the community](#Step7)
3. [Learn the literature](#Step3) 8. [Work diligently to accomplish goals and objectives](#Step8)

4. [Explore existing resources](#Step4) 9. [Develop tools for effective evaluation](#Step9)

5. [Develop your road map](#Step5) 10. [Regularly re-evaluate and reflect on plan and](#Step10)
6. [Build a coalition](#Step6) [project-related work](#Step10)

**Step 1: Identify the Problem**

As previously described, advocacy starts with passion about a particular issue, frustration about a specific problem, and/or a desire to work with a specific community or population. In real life, if you are going to work hard to effect change, you should do it because the issue(s) you are working for resonates powerfully with you. As a physician—and as a human being—you will find problems and/or populations that resonate deeply and inspire you to work toward positive change. Effective advocates discover the issues and populations they fight for in the course of their clinical practice and everyday lives, and not because they are mandated to advocate for something or someone. They embrace problem and/or population that finds them.

For this particular learning activity, you may not have the luxury of “organically” discovering an advocacy-related passion. You will need to identify and focus on a specific issue to complete this activity, which admittedly may feel a bit forced for some individuals.

Residents completing this activity often fall into 1 of 3 common scenarios in selecting an area of interest upon which they will focus:

- The choice of a particular issue or population is “obvious,” due to previous interest, expertise and (perhaps) involvement with an issue/population
- A resident may feel passionately about multiple issues, have many advocacy-related ideas, and wrestle with narrowing their focus to a single, specific issue
- A resident may not yet have developed a passion related to particular issue and experiences difficulty in pinpointing a topic about which he/she feels enthusiastic about enough to commit.

Regardless of the scenario with which you identify, it will be important to settle on an issue or population of focus by the end of the first week for the purpose of completing this learning activity. As they say at Nike, “Just do it!”

Another essential step in developing your community-based advocacy project proposal involves defining “your community;” You must be explicit about whom you think your “community” is; whose voice needs to be heard? For the purpose of this exercise, will your “community” involve all children in your city? Will it involve a subset of those children (i.e., students at one elementary school or a population of Somali immigrant children living in a particular neighborhood)? Be very specific about defining what population “your community” entails.

**My community is:**

**Mission, Vision, and Values:**

Now that you have settled on an advocacy focus and have defined “your community,” the next key step is to define your advocacy-related mission. Your mission describes your overall purpose for advocating for your particular issue or population. Developing this mission statement will help focus your thought process; while you may revise your mission over time, you will achieve a certain clarify of purpose by identifying why you are doing this work

**Develop your mission statement:** This should be a brief, one-sentence summary of your overall purpose related to your proposed project. The mission statement should provide a succinct, broad overview of your purpose, ideally summarized in 20 words or less.

Example of a mission statement: *To increase the number of children at Wilson Elementary School who receive weekend food backpacks.*

**What is your Mission?**

**Next, develop your vision statement:** Your vision statement should describe the change you hope to achieve, in broad terms. Your vision statement should relate directly to your mission. One way to think about defining a vision statement is to consider, “Once I have successfully implemented this project, THIS WILL HAPPEN!”

Example of a vision statement: *No child at Wilson Elementary School will go hungry on weekends or holidays.*

**What is your Vision?**

**Finally, develop a Values Statement**: Your values reflect the key principles that will inform and guide your work. They are core beliefs that you hold dear, that you will not compromise in the process of developing and implementing your project. They will serve as your “moral center” and keep you focused on what is important.

Examples of a values statement:

*Do what is best for kids*

*Focus on meeting the needs of the community in the community*

*A comprehensive approach, from the individual child up to the policy arena will achieve the best results*

**What are your values?**

**Step 2: Define the Baseline**

*Data drives everything.*

Everything you propose and implement must be based on the best available evidence. Having data related to your advocacy focus will drive your progress, enable you to assess the efficacy and impact of your efforts, and remains among the most powerful ways to influence change-makers.

For your area of interest, what is known about the community you have defined and the issue you have chosen to address? It is possible that your work may need to begin as a needs assessment, or a simple study to assess the extent of the problem you have chosen to focus on. Does any baseline data exist? Where can you find it? How can you measure it if it is not already known?

This could be thought of as being analogous to building the foundation for a house—you own the “land” (the project’s mission/vision/values), the “house” you intend to build will be the completed advocacy project, and the house’s foundation will provide its strength and stability. In this case, the project’s “foundation” is the background information and data about your chosen community and your issue.

As you develop your advocacy project, it is important to determine what data you need to know in order to develop a well-designed, meaningful project that will truly make a difference. Once you have determined what data is needed, the next step involves ascertaining whether someone has already collected it. If so, was this data collected in your own community, or was it collected elsewhere? (If elsewhere, can the available data be extrapolated to your own community and project?) Where are “the holes” (i.e., what data still needs to be collected)?

- What do you need to know to make your ideas meaningful?
- Has someone already collected this information? If so, was it in your community of interest, or somewhere else?
- Where are the “holes” in your data foundation?

**Here is the data I already have to inform the development and implementation of my project (i.e., what I already know):**

**Here is information/data that I will need to develop my ideas, but do not already have:**

We will come back to this point again, but it is very important to have a clear understanding of the needs of your community, as defined by that community. This can be achieved through a needs assessment of some sort- existing literature, surveys, focus groups etc. What information do you already know, and what might you want to know, from the community perspective? While learning everything about how to perform a community needs assessment is beyond the scope of this exercise, you can learn more about the process if you like here: <http://www.acf.hhs.gov/programs/ocs/resource/conducting-a-community-assessment-1>

**Step 3: Learn the Literature**

*It is essential to build content expertise related to the advocacy issue (and/or population) that you have chosen.*

To serve as an effective advocate for a specific issue or population, it is essential to develop a strong knowledge base specific to that issue. In simple terms, “To walk the walk, you must be able to talk the talk.” For instance, you cannot effectively advocate for issues related to improving the foster care system if you do not have a good knowledge base about foster care in your community, a clear sense of how the foster care system works, an understanding of who makes decisions related to the foster care system in your community, and a knowledge of what the “state of the art” is in the literature regarding foster care. What steps do you need to take to develop this knowledge base related to your advocacy area? You may be a true novice in terms of your chosen advocacy topic, or you may already understand the content area quite well. Either way, you will likely have to learn a lot more to be truly effective in effecting change.

Consider meeting with your local medical librarian for assistance with identifying the current literature related to your specific advocacy issue (or population) and obtaining the resources that you will need to pursue in order to build your content expertise.

**Share a small bibliography of resources that you identified that will help you build expertise in your chosen area:**

**Step 4: Explore Existing Resources**

*What initiatives/projects already exist or have been tried in the past (locally, regionally, and beyond)? Who is already working on this, and who will be your partners, mentors, and advisors? It is important to realize that other experts may have already explored your problem/issue of interest and are already involved in advocacy efforts related it.*

You may discover examples of prior advocacy efforts that have been successful that may closely mirror your own ideas - you need to know these - and who made them work - so you can learn from their successes. You may discover that someone has tried to implement an intervention that is similar to your idea, and that it was largely unsuccessful. If so, you will certainly want to learn from these examples as well to avoid repeating mistakes. For instance, consider the example of the very important issue of childhood obesity:

If simply counseling families during well-child visits was effective in decreasing obesity, our society would be in much better shape (both literally and figuratively). Unfortunately, this intervention - which many others have tried previously - has been found to be ineffective in decreasing childhood obesity. Therefore, a project based solely on obesity-related counseling during well-child visits will very likely *not* work as the best approach to addressing your community’s needs as related to the issue of childhood obesity.

You also need to be able to identify the content experts in your area of interest. For example, if you are interested in developing a project to increase bicycle helmet use among children, it is important to identify the individuals within your community who are considered the authorities and “champions” on this issue. As you work to identify these experts, you will want to ascertain whether there someone in your community or region who is a guru, or whether you should look nationally to other institutions, the private sector, or to government resources to find the experts? Once you identify these experts, be prepared to reach out to them - most would like nothing more than to engage with and mentor an enthusiastic pediatrician who is passionate about his/her area of expertise.

Make some lists:

**What exists in my community (your city and environs)?**

**What exists in my state or region?**

**What exists nationally? Where is it?**

**Identifying and Engaging Community Partners and Expertise:**

In planning and implementing a community-based project, it is very important to understand that you will be able to accomplish a great deal more through working in collaboration with others than you ever will be when attempting to venture out alone. You will need to develop a coalition of individuals and groups that can help you plan and implement your ideas in order to achieve your goals. You will need to identify leaders in your target community as well. Your coalition may include some of the local/regional/national experts and individuals involved with current efforts related to your issue/population that you have already identified above. You will do more focused coalition development later, but it is important to start thinking about your team now.

**Review the Community Partner Finder to help you think through categories of individuals and groups who can work with you in achieving your goals.**

**Here is a list of people or groups I will need to engage:**

**Step 5: Develop Your Road Map**

*Goals and objectives*

(The following is adapted from the AAP *A Pediatrician’s Guide to Proposal Writing* **(**[**https://www2.aap.org/commpeds/resources/grant_writing.html**](https://www2.aap.org/commpeds/resources/grant_writing.html)**)**

Essential parts of any project proposal are well-written goals and objectives. They describe the purpose of your project, what you specifically aim to accomplish, and how you will evaluate whether the project achieved its intended aims. The following information on goals and objectives should help you in writing your own. Please note that there are a variety of ways to write objectives as is evident in the examples given below.

**Goals**

Goals are broad statements of what your project will accomplish. They generally are conceptual and abstract, and not measurable. They may mirror your mission statement- that is completely fine.

Example 1: *Improve the health status of adolescents living in Cook County, Illinois.*

Example 2: *Decrease the infant mortality rate in rural Nebraska.*

Think about the goal as the ultimate destination of your trip- where do you hope to end up?

**Objectives**

Objectives are the measurable steps that will be used as guidelines for evaluation. They are the individual steps that will allow you to reach your destination, like the specific steps in a map.

All of your objectives should be SMART:

**S**pecific

**M**easurable

**A**ttainable

**R**elevant

**T**argeted to community

Objectives generally answer the following questions:

- What are you going to do?
- How will you do it?
- By when will you do it?
- How much change do you expect?

Objectives usually consist of the following three components:

- A single target health indicator or activity to be measured
- A target population
- A time frame

**Types of Objectives**

In proposal writing, there generally are four types of objectives: behavioral, performance, process, and product. Your project may use more than one of these types of objectives. Most importantly, project objectives need to be easily understood, clearly written, and realistic.

**Behavioral Objectives**

Behavioral objectives measure the desired change in knowledge, attitudes, beliefs, or behaviors that are expected to occur as a result of the project.

Example: *Eighty-five percent of the parents surveyed following the parenting education program will be able to identify three strategies to reduce childhood injuries in their home.*

**Performance Objectives**

Performance objectives measure the desired change in which a desired behavior will be adopted in a given time period. This time period usually refers to the length of the project period.

Example: *By the end of December 31, 2000, increase by 30% the number of children who participate in regular cardiovascular physical activities.*

Example: *By June 30, 2000, increase to 90% the number of children who have been fully immunized.*

Behavioral and performance objectives generally measure the proportion of the target population expected to show a change in the indicator or the amount of change expected in the indicator.

**Process Objectives**

Process objectives measure the procedures and tasks involved in implementing a project.

Example: *Provide at least 10 parenting workshops that promote injury prevention strategies to prevent or reduce the risk of injury to families.*

Example: *Distribute at least 10,000 materials and brochures about household injury prevention to parents of children from birth to 2 years old.*

**Product Objectives**

Product objectives measure the production of tangible items that are part of the project.

Example: *Create a notebook of reproducible meeting materials, including incentives, a template meeting agenda, and resources for communities interested in convening collaborative meetings.*

If your goals are where you want to end up, then the objectives are the individual steps that must be completed to get there. Think about a road trip to Vegas: Vegas is your goal. Your objectives are the segments of the journey that will ultimately get you to your destination (first day driving, second day driving- which roads you take, where you stop, etc.)

Think about your goals. These relate back to your mission and vision statements. What do you hope to achieve? What are the steps that must be completed in order to achieve your goal?

Let’s consider an example:

Imagine that you are caring for a patient in the ED who fell from a play structure at school, hit her head, and complained of neck pain. The EMT’s found her sitting up in the nursing office at the school, and grumble about how school playground personnel have no clue how to triage and care for basic injuries.

Consider what you could do to improve this situation. Where are the opportunities for change? Does the problem you want to address involve the school play structure itself, or the surface the child fell onto? Is this an issue with a child with ADHD who does not have systems in place to protect her? Does it involve the training of the playground aid who should have done something differently, or is the problem you want to address that schools in general have unsafe playgrounds?

Let’s say you decide that the problem is the process for treatment and triage of playground-related injuries occurring in your school district - and, more specifically, the school that your patient came from. Examples of goals/objectives you might choose to develop for your project are shown as follows:

Goals: To train Playground Aides at Wilson Elementary School in basic first aid, and in basic principles related to treatment and triage of playground-related injuries

Objectives:

- - - - Meet with the Head Nurse for the School District within the next 2 weeks.
      - Review the statistics related to reported injuries on the school playgrounds in our School District by week 3
      - Meet with the Principal and School Nurse of Wilson Elementary School to discuss implementing a pilot training program by week 5
      - Review Basic First Aid Principles, and develop a pilot curriculum for training playground aids by week 8
      - Meet with the EMS Coordinator to help develop a training curriculum. Work with EMT’s to develop materials and a script for the training presentation by week 12
      - Train 100% of the playground aides who work at the Wilson Elementary by week 16.

The following checklist can serve as a guide to help you decide if you are ready to finalize your project objectives:

- Are the objectives clear and easy to understand? Are they outcomes based? Are the outcomes included within each objective specific, measurable, attainable?
- Do the objectives include a single element that can be measured?
- Do the objectives use a realistic timeline? Are they realistic given your limitations and goals?
- Do the objectives represent a minimum level of accomplishment—are they truly achievable?

Now it is your turn to practice developing your own project-related goals and objectives:

**What are your goals? List what you want to accomplish through your advocacy:**

**Now list your objectives- what are the steps to helping you achieve success? How do you meet your goal(s)?**

List your objectives, and you see them now- be honest, some may be easy, some may be near-impossible. If you are not specific, however, you will never know what you need to achieve. Answer the question: By the time I am done, here is what will exist/ what will happen…

Once you have listed your objectives, take an honest, critical look at them: Some may be rather easy to accomplish, while others may seem nearly impossible. Keep in mind that your objectives should be specific. As you read each of your objectives, you (and anyone else reading each objective) should be able to answer the question: “As a result of my project/intervention, what will exist/what will happen (define how much of what will exist or happen) by when (clearly specify timeframe)?

**Now go back and look at your Mission, Vision and Values.**

Your goals and objectives should align with your Mission, Vision and Values. Drifting away from these should be a very important cue that you need to rethink your goals and objectives. If you set goals about what you wanted to achieve, and defined what was important to you, you need to stay true to these principles. If you find that your goals and objectives do not align with these principles, you need to either rethink your goals and objectives, or redefine what is important to you in your work.

How does your Mission/Vision/Values align with your goals and objectives? Which needs to change?

**Please jot down your thoughts on the alignment, and, your reformulated, better aligned Mission/Vision/Values or Goals and Objectives:**

**Step 6: Build Your Coalition**

*Think about who will help you in planning, in implementation, and never forget to know your opposition!*

In planning and implementing a community-based project, it is very important to understand that you will be able to accomplish a great deal more through working in collaboration with others than you ever will be attempting to venture out alone.

You started identifying your team above. Now that you have developed a clear sense of what you hope to achieve, it is time to really flesh out whom you need to engage. You will need to develop a coalition of individuals and groups that can help you plan and implement your ideas in order to achieve your goals. Your coalition may include some of the local/regional/national experts and individuals involved with current efforts related to your issue/population that you have already identified.

One principle that can be very useful in identifying the people and groups to engage in your coalition is Asset Based Community Development.

Whatever you do, wherever and whenever you do it, you will not be able to do anything alone. You will need to develop a coalition of people and groups that can help you plan and implement your ideas to help you achieve you goals. Some of these may overlap with your local/regional/national experts and the list of resources in your community.

Think about who will help you plan, learn what you need to learn about your problem and your community, and who can help you craft your solution. Your team will likely have both thinkers and doers; there will likely be some overlap, and your list will not be complete by any means.

**Identifying and Engaging Community Partners and Content Experts**

Identify Resources: Identify and connect with the individuals who can aid you in developing and implementing your proposed project. Reach out to these individuals and meet with them (whenever possible) to discuss ideas, refine your proposal, and enlist support for the work you are planning! Utilize the “Community Wheel” as a guide throughout this process. Consider which individuals might be able to assist you in both planning and implementation of your project. These may include members of local community groups, or even regional or national content experts. Although identifying these project “champions” and engaging with them may sometimes seem daunting, individuals who are passionate about a particular advocacy issue or population will likely be thrilled about collaborating on a project related to the issue or patient population that they care about so deeply.

**Asset Based Community Development**

This is the “glass is half-full” approach to working within a community to effect positive change. Instead of simply focusing on the problem within the community, this approach involves taking stock of what knowledge/skills and attitudes you (and your project) already possess, and what assets still need to be brought to the project through the development of partnerships. There is likely already tremendous power and capacity in the community in which you will be working - locating and harnessing this existing capacity is a crucial step in the project development process. Throughout this process, you may often need to serve as the “glue” that brings together the disparate parts of your coalition. Consider what assets and skills you will require to plan and implement your idea. In considering the assets within a community, there are 5 major “categories” of support that can be brought together in synergy in the process of planning and implementing a community-based project. These are listed below (taken from ABCD Institute, Northwestern Univ. [www.abcdinstitute.org/](http://www.abcdinstitute.org/) ):

1. Talents and skills of individuals
   - Who do you know?
   - What can they do?
     - Think about your “planning” partners and your “doing” partners- some might be both!
2. Associations and networks of relationships?
   - What do you need?
   - Who do you and your collaborators already know?
     - Remember “6 degrees of separation…”
3. Institutions and Professional Entities
   - Groups, clubs, corporations, governments etc. These are often the locus of expertise, money and power, and therefore it is very important to become familiar with these entities.
4. Physical Assets
   - Land
   - Property
   - Buildings
   - Equipment
5. Economic Assets
   - Sweat equity
   - Consumer spending power
     - Money is power- people control where they spend it. This translates into power…
   - Local Business Assets
     - As above, money is power; who already has it in your community?

As you consider bringing community assets together, it is important to reflect on the following questions: How do you get people’s attention? How to do you influence the status quo and effect change? This may be the hardest part - the dreaming is easy. It is the translation from dreaming to doing that can be particularly challenging.

**Here is a Ridiculously Exhaustive Master List of Potential Community Groups and Associations** whom you could potentially engage with in the process of developing an implementing a community-based project. This list is provided to demonstrate the breadth of the potential assets within a community. Some of these may exist in your community, while some may not. (We bet you did not think of a Kite Flying Club!)

- Addiction prevention recovery groups
  - Drug ministry/Testimonial group for addicts
  - Campaign for a Drug Free Neighborhood
  - High School Substance Abuse Committee
- Advisory Community Support Groups
  - Friends of the Library
  - Neighborhood Park Advisory Council
- Animal Care Groups
  - Cat Owner’s Association
  - Humane Society
- Anti Crime Groups
  - Children’s Safe Haven Neighborhood group
  - Police Neighborhood Watch
  - Senior Safety Group
- Athletic Groups
  - Soccer clubs
  - Little leagues
- Block Clubs
  - Condo Owner’s Association
  - Building Council
  - Tenant Club
- Business Organizations/Support Groups
  - Jaycees
  - Local Chamber of Commerce
  - Economic Development Council
  - Local Restaurant Association
- Charitable Groups and Drives
  - Local hospital auxiliary
  - Local United Way
  - United Negro College Fund Drive
- Civic Events Groups
  - Local parade planning committee
  - Arts and crafts fair
  - July 4^th^ Carnival committee
  - Health fair committee
- Cultural Groups
  - Community choir
  - Drama club
  - Dance organization
  - High school band
- Disability/Special Needs Groups
  - Special Olympics planning committee
  - Local American Lung Association
  - Local Americans with Disabilities Association
  - Local Muscular Dystrophy Association
- Education groups
  - Local school council
  - Local book clubs
  - Parent Teacher Association
  - Literacy Council
  - Tutoring groups
- Elderly Groups
  - Hospital senior groups
  - Westside senior clubs
  - Church senior clubs
  - Senior craft club
- Environment Groups
  - Neighborhood recycling group
  - Sierra Club
  - Adopt-A-Stream
  - Bike path committee
  - Clean air committee
  - Pollution Council
  - Save the Park committee
- Governmental groups
  - City government
  - State government
  - Federal government
- Family Support Groups
  - Teen parent organization
  - Foster parents’ support group
  - Parent Alliance group
- Health Advocacy and Fitness Groups
  - Weight Watchers
  - YMCA/YWCA Fitness groups
  - Neighborhood health councils
  - Traffic safety organizations
  - Child injury prevention groups
  - Yoga club
- Heritage Groups
  - Black Empowerment Group
  - Norwegian Society
  - Neighborhood historical society
  - African American Heritage Society
  - La Raza
- Hobby and Collectors Groups
  - Coin collectors association
  - Stamp collectors association
  - Arts and crafts club
  - Garden club of neighbors
  - Sewing club
  - Antique collectors
- Men’s Groups
  - Fraternal orders
  - Church Men’s Organizations
  - Men’s sports organizations
  - Fraternities
- Mentoring Groups
  - After school mentors
  - Peer mentoring groups
  - Church mentoring groups
  - Big Brothers, Big Sisters
  - Rights of Passage Organizations
- Mutual Support Groups
  - La Leche League
  - Disease support groups
  - Parent-to-Parent groups
  - Family-to-Family groups
- Neighborhood Improvement Groups
  - Neighborhood garden club
  - Council of Block Clubs
  - Anti-Crime Council
  - Clean-up Campaign
- Political Organizations
  - Democratic Club
  - Republican Club
- Recreation Groups
  - Kite-flying club
  - Bowling leagues
  - Basketball leagues
  - Body Builders club
  - Little League
- Religious Groups
  - Churches
  - Mosques
  - Synagogues
  - Men’s Religious Groups
  - Women’s Religious Groups
  - Youth Religious Groups
- Service Groups
  - Zonta
  - Optimist
  - Rotary Clubs
  - Lions Club
  - Kiwanis Club
- Social Group
  - Bingo club
  - Card playing club
  - Social activity club
  - Dance Club
- Social Cause/Advocacy/Issue Groups
  - Get out the Vote Clubs
  - Peace Club
  - Hunger Organizations
  - Vigil Against Violence
  - Community Action Council
  - Social Outreach Ministry
  - Soup Kitchen Group
- Union Groups
  - Industrial (UAW)
  - Grafts
  - Union (Plumbing Council)
- Veteran’s Groups
  - Veterans of Foreign Wars
  - Women’s Veteran Organizations
- Women’s Groups
  - Social Organizations
  - Women’s Sports Groups
  - Women’s Auxiliary
  - Mother’s Board
  - Eastern Star
- Youth Groups
  - After school group
  - 4-H
  - Girl and Boy Scouts
  - Junior Achievement
  - Campfire Girls

There are some people or groups who may not be expected to do the work in addressing your issue, but you may need to **COMMUNICATE** with for educational, strategic or political reasons. Think about who will help you **PLAN** your advocacy work (your thinkers and ambassadors to the community). Think about who will help you **IMPLEMENT** your ideas (your doers). While some people/groups may fill only one area, some may fill 2 or 3.

Make certain to consider representatives from the community with whom you are working- this will be discussed further in the next section. Fill in the table on the next page to start building your coalition! If you have more than 8 ideas, just add them in!

| **Name of group or individual** | **Communicate** | **Plan** | **Implement** |
| --- | --- | --- | --- |
|  |  |  |  |
|  |  |  |  |
|  |  |  |  |
|  |  |  |  |
|  |  |  |  |
|  |  |  |  |
|  |  |  |  |
|  |  |  |  |
|  |  |  |  |

Finally, it is extremely important to understand that as you work as an advocate within your community, there will always be potential barriers to change, as well as individuals who may oppose your project. This opposition may be motivated by physical, financial, emotional, historical, political, or even irrational concerns. As you work to implement your advocacy project, it is important to foresee any potential opponents who could derail your efforts to improve the well-being of your community and even talk with these individuals to understand their perspective related to your area of interest and/or planned project.

**List your potential OPPONENTS here- acknowledging that your biggest foes may be the last people you thought they would be!**

**Step 7: Ensure that you are Doing Things WITH the Community, not TO the Community.**

Your project should:

- - Be outcomes-oriented
  - Have a measurable impact
  - Be relevant and valuable to the community

It is important to ensure that you are not forcing your will upon an unwilling audience! Even though you are honest, ethical and sincere in your work, you cannot assume that you are speaking for your community—or presume to know what they truly want and need—without including community members in the process!

Review your goals and objectives once AGAIN, checking for 2 key elements:

1. Make certain that your objectives can be measured- that they are tied to specific outcomes which can be monitored and reported, both at baseline and after you have been working. To verify that your project is effective or valuable, you must be able to measure its outcomes.
2. As discussed above, it is critical to make certain that you have asked and listened to the community with whom you are working to ensure it is their voice that is being represented, not yours. There is a tremendous tendency for physicians to want to speak for their patients. While this can certainly be appropriate at times, it is critical to truly engage the actual members of a community in defining their community’s problem(s) and developing the solutions to them. Review your project goal(s) and objectives from the perspective of ensuring they focus on the community’s needs, assets, and solutions (and not just yours). Jot your reflections on this here:

**Step 8: Work Diligently to Accomplish Your Goals and Objectives**

*Start small and fail small, but dream big.*

At this point, you hopefully have a better understanding of the many elements involved in planning a community-based advocacy project. The effort required to complete the necessary groundwork can certainly be huge, depending on the scope of the planned projects. It can take weeks, months, and even years before one is actually ready to implement the work they have set out and planned to do! In the realm of advocacy, patience and perseverance are essential! If you try to implement a project too early, before all the essential preparatory steps have been completed, you will likely set yourself up for failure. This concept can be compared to preparing a special meal - if you start prepared with a specific, proven recipe, the right amounts of quality ingredients, and proper equipment and technique, you will likely succeed. In any kitchen, the preparation can take hours, with a delicious meal is the end-result. One must have patience and perseverance to get there!

Like anything important in life, it can be both disheartening and discouraging to fall short of one’s goals and objectives in advocacy-related work, especially when the work involves a topic that you feel very passionate about. As pediatricians, we are largely a group of dreamers. While it is essential to “dream big,” it is equally essential to remain realistic about what we can reasonably achieve during the implementation phase of our work. As with our special meal, if we try to cook something that is beyond our capability, we are unlikely to succeed. We will all experience failures and setbacks in our work, sometimes due to mistakes or miscalculations we make, and sometimes for reasons beyond our control. It is much easier to correct small failures than colossal ones.

At times, it is best to scale back our original goals and objectives in a stepwise fashion, so that we plan a series of small successes that can translate into a significant positive changes for a community overall, as opposed to trying to solve all of the problems related to a particular issue overnight. This also means our failures will be smaller and easier to circumvent.

Stop and reflect on the project you have planned. Does it seem feasible, too big, or too narrowly-focused? Write a few reflective statements about your plans, in terms of their feasibility, putting them into a realistic perspective- do your goals and objectives seem too “big,” too small, or just right?

**Step 9: Develop Tools for Effective Evaluation**

*Prove what you are doing works.*

We have talked a lot previously about identifying tangible and measurable outcomes for your project. If you are going to ask for money, time, influence or anything else from others, you must be able to demonstrate to them that what you are doing (or will do) is effective in the overall sense, and also that you have been able (or will be able) to achieve your specific objectives. In planning to evaluate your project, it can be helpful to answer the following questions:

Do you have a specific plan for measuring outcomes (overall numbers of individuals impacted, surveys designed to measure change, epidemiological measures, etc.)?

- What are your specific outcomes measures? They should be aligned with your objectives.
- Will you need anything else to demonstrate efficacy of your program?
- How will you and everyone else know if it worked?
- Think about timing, about short, medium and long term goals and what the specific measures of success might be.

Fill in the Action Step Chart below. Be as detailed as you can be, identifying who will do what, specifying timing as well as you can, what you will need, and how you will know if that step was successful.

This can be daunting, but remember, this is a thinking exercise, so it does not have to be perfect. Do your best to think through the process.

| **Action Step** | **By Whom** | **By When** | **Resources and Support Needed** | **Possible Opponents** | **Evaluation Measures** |
| --- | --- | --- | --- | --- | --- |
|  |  |  |  |  |  |
|  |  |  |  |  |  |
|  |  |  |  |  |  |
|  |  |  |  |  |  |
|  |  |  |  |  |  |

**Step 10: Regularly Re-evaluate and Reflect on Your Plan and Your Project-related Work.**

*What are you doing that works? What do you need to do differently to improve upon your project?*

Think Quality Improvement, think PDSA (Plan-Do-Study-Act)…Duke University School of Medicine has some great resources for thinking about Quality Improvement: <http://patientsafetyed.duhs.duke.edu/module_a/methods/pdsa.html>

Every resident must engage in some QI work as part of their training, and the skills learned there are no different that those needed to ensure effective advocacy work. A key to being an excellent pediatrician must include the ability to practice reflectively. Reflective practice involves consciously “stepping back” and reflecting on each patient interaction, each day, each rotation, and each and every endeavor you undertake in order to reflect on and identify what you are doing that works well, as well as what do you need to do differently and how you will make necessary changes.

Such practice serves as the basis for formal Quality Improvement initiatives—critically examining current practice, identifying areas where improvement is needed, implementing changes, and measuring outcomes to determine if the changes implemented are actually improving practice.

Practicing reflectively in a consistent, conscious manner is an invaluable skill, in advocacy work as well as in clinical practice. Acknowledging this and determining your own methods for reflective practice will help to ensure your success, both as a pediatrician and a child health advocate.

Spend a few minutes reflecting on what you have learned through this process? Was it harder or easier than you thought? Did you pick the right area? What would you change if you were going to go forward with your project?

**Congratulations!**

You have now completed all of the steps that are essential in developing a community-based advocacy project. If this is where your adventure as an advocate ends for now, you have developed a valuable skill set that can be applied to advocacy endeavors you may choose to pursue in the future.

If the issue you defined through this activity is one that has “found you,” then you should certainly continue to pursue it! All pediatricians must serve as advocates for children - for those in seen in their own clinical practices, as well as for *all* children, who are in need of skilled, committed individuals to speak out and work on their behalf. The goal of this learning activity is to train pediatricians to advocate effectively; through this work, you have begun to develop the skills you will need to do this throughout your career. As with other skills, practice is essential to developing proficiency. We encourage you to use these skills often - thoughtfully, compassionately, and enthusiastically - as you serve children and help to improve their well-being throughout your career as a pediatrician.
