# Supplementary material for: The Community Pediatrics Training Initiative Project Planning Tool: A Practical Approach to Community-Based Advocacy
Source: MedEdPORTAL. 2017 Sep 18;13:10630. doi: 10.15766/mep_2374-8265.10630 (PMC6338167; doi:10.15766/mep_2374-8265.10630)
Supplement: Supplementary file 1 — A. CHAMP.pdf B. CHAMP Mapping Tool.pdf C. AAP CPTI Project Planning Tool.docx D. AAP CPTI Project Planning Tool Milestones-Based Assessment Rubric.docx E. Project Planning Tool Users Guide.pptx [file mep-13-10630-s001.zip › D. AAP CPTI Project Planning Tool Milestones-Based Assessment Rubric.docx]

**Project Planning Tool: Developing a Community Advocacy Project Proposal**

**Assessment Rubric**


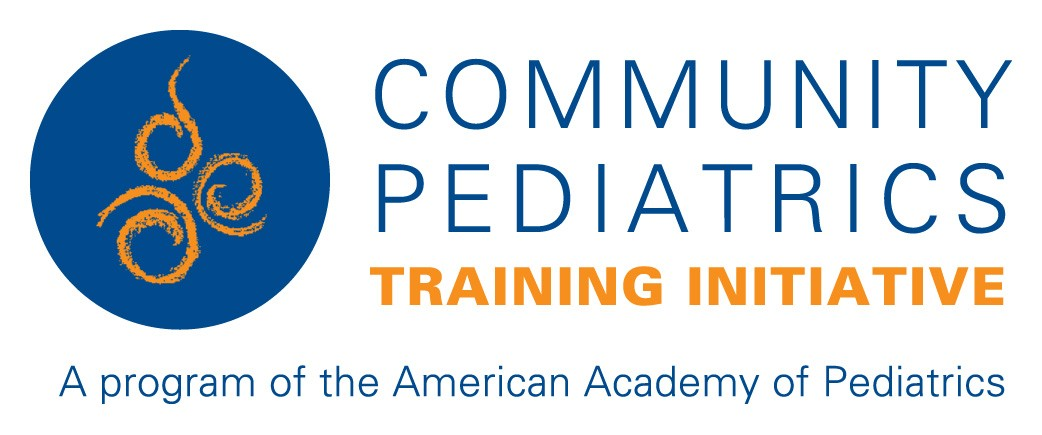


|  | **1** | **2** | **3** | **4** | **5** |
| --- | --- | --- | --- | --- | --- |
| **10 Steps for Community-based Advocacy**  Pediatrics Milestone Number (Refer to key on page 4.) | **Inadequately Addressed** | **Fell Short of Expectations** | **Met Expectations** | **Exceeded Expectations**  **Advanced Skills** | **Exceeded Expectations**  **Fully Proficient** |
| **Developmental Milestones** | | | | | |
| **Step 1: Identify the Problem** | **Poorly defined issue, is not able to specifically state the problem. Community poorly defined.** |  | **Defines a problem and is able to articulate the issues clearly. Community is defined.** |  | **Problem is well-defined and specific. Community is identified and clearly well understood.** |
| Identifies an issue that relates to child health and well-being **SBP2***; **PROF2; (**SBP7; PBLI10)** |  |  |  |  |  |
| Clearly defines target population/community  (SBP7) |  |  |  |  |  |
|  | | | | | |
| **Step 2: Define the Baseline** | **Minimal to no data. Unable to assimilate information into plans clearly.** |  | **Data clearly represents the community. Demonstrates understanding of application of that data to the issue.** |  | **Very detailed data collection and analysis. Level of understanding approaching expert.** |
| Locates, appraises, and assimilates evidence from community health data to develop expertise about the advocacy topic  (PBLI6) |  |  |  |  |  |
|  | | | | | |
| **Step 3: Learn the Literature** | **Inadequate search. Unable to demonstrate knowledge on the issues.** |  | **Effective search and clear understanding of the issues.** |  | **Exhaustive search with extensive bibliography. Level of understanding approaching expert.** |
| Locates, appraises, and assimilates evidence from scientific studies and community health data to develop expertise about the advocacy topic (PBLI6) |  |  |  |  |  |
|  | | | | | |
| **Step 4: Explore Existing Resources** | **Unable to identify resources.** |  | **Clear ability to recognize and find resources at various levels.** |  | **Comprehensive catalog of existing resources at all levels.** |
| Categorizes existing community resources (locally, statewide and nationally) needed to help advance advocacy agenda **SBP2**; **Prof6;** (PPD6) |  |  |  |  |  |
|  | **1** | **2** | **3** | **4** | **5** |
| **10 Steps for Community-based Advocacy**  Pediatrics Milestone Number (Refer to key on page 4.) | **Inadequately Addressed** | **Fell Short of Expectations** | **Met Expectations** | **Exceeded Expectations**  **Advanced Skills** | **Exceeded Expectations**  **Fully Proficient** |
| **Developmental Milestones** | | | | | |
| **Step 5: Develop Your Road Map** | **Goals and objectives inadequate and not aligned with needs of community.** |  | **Goals and objectives are SMART^1^ and consider needs of community.** |  | **Detailed, SMART objectives, well aligned with community. Program could launch tomorrow.** |
| Defines measurable goals and objectives as they relate to the advocacy topic (SBP1; SBP7; PBLI2)  *^1^SMART= Specific, Measurable, Attainable, Relevant, Targeted to the Community* |  |  |  |  |  |
|  | | | | | |
| **Step 6: Build Your Coalition** | **Does not identify key coalition members or possible opposition.** |  | **Identifies a number of coalition members from across the community and beyond, and delineates potential opposition.** |  | **Exhaustive list of coalition members, able to stratify by role and level of involvement. Potential opposition and threats to success fully identified.** |
| Clearly outlines potential partners (and potential opposition) to communicate, plan, and implement project  **ICS1**; **SBP1;** (ICS3; ICS4; PBLI8; PBLI9; PPD6) |  |  |  |  |  |
|  |  |  |  |  |  |
| **Step 7: Ensure Project Includes Voice of Community** | **Voice of community absent. A proposal to do something TO a community, not WITH a community.** |  | **Voice of community present and collaborative efforts are included.** |  | **Evidence of authentic partnerships. Emphasis clearly on the community.** |
| Identifies assets within the community and describes plan to include and respond to input from community stakeholders in project planning and implementation  **ICS1**; **ICS2**; **PBLI4**; **SBP1;** (ICS3; ICS4) |  |  |  |  |  |
|  |  |  |  |  |  |
| **Step 8: Revisit Goals and Objectives** | **Unable to effectively reflect. Unable to accept feedback.** |  | **Able to articulate need for change based on experience.** |  | **Clearly identifies both need for change, as well as clearly articulates a plan to do so.** |
| Reflects on short- and long-term feasibility of goals and objectives to enhance population health  **PBLI3;** (PBLI10; SBP1; SBP7) |  |  |  |  |  |
|  | **1** | **2** | **3** | **4** | **5** |
| **10 Steps for Community-based Advocacy**  Pediatrics Milestone Number (Refer to key on page 4.) | **Inadequately Addressed** | **Fell Short of Expectations** | **Met Expectations** | **Exceeded Expectations**  **Advanced Skills** | **Exceeded Expectations**  **Fully Proficient** |
| **Developmental Milestones** | | | | | |
| **Step 9: Develop Tools for Evaluation** | **Minimal plans for evaluation.** |  | **Evaluation plan will measure some changes, focus on knowledge change.** |  | **Meaningful evaluation, measures of behavior change or health status included.** |
| Identifies methods for measurement of short term, intermediate and long range goals and objectives  **PBLI3;** (SBP1) |  |  |  |  |  |
| **Step 10: Reflect on Plan** | **Unable to employ reflection and QI principles to identify opportunities for change.** |  | **Reflection leads to identification of areas in need of transformation. Able to discuss how plans would be altered.** |  | **Sophisticated reflections lead to concrete and nuanced discussion of transformation of proposal.** |
| Uses reflection to identify and perform appropriate learning activities and evaluate one’s own work in spirit of self-improvement to enhance population health  **PBLI3**; **PROF1**; **PROF2** |  |  |  |  |  |
|  | | | | | |
| **Overall Evaluation** |  |  |  |  |  |
| Demonstrates knowledge and skills necessary to fulfill the role of advocate for children.  **SBP2**; **PROF1**; **PROF2;** (PBLI10) |  |  |  |  |  |
| Areas of Particular Strength: | | | | | |
| Areas of Focus for Future Learning: | | | | | |

*** BOLD: Milestones currently being reported with New Milestones number.**

****** ( ): Milestones not yet reported and labeled according to the Original Pediatric Milestones Project^1^; see key below.

Pediatrics Milestones Key

Milestones by Original Designation with **BOLD** Designation of those currently reported**^1^**

| Milestone Number  (**Bold= Currently reported with New Milestones number)** | Description |
| --- | --- |
| SBP1 | Work effectively in various health care delivery settings and systems relevant to their clinical specialty |
| **SBP2 (SBP1)** | Coordinate patient care within the health system relevant to their clinical specialty |
| **SBP4 (SBP2)** | Advocate for quality patient care and optimal care systems |
| SBP7 | Know how to advocate for the promotion of heath and the prevention of disease and injury in populations |
| **ICS1 (ICS1)** | Communicate effectively with patients, families, and the public, as appropriate, across a broad range of socioeconomic and cultural backgrounds |
| **ICS2 (ICS2)** | Demonstrate the insight and understanding into emotion and human response to emotion that allow one to appropriately develop and manage human interactions |
| ICS3 | Communicate effectively with physicians, other health professionals, and health related agencies |
| ICS4 | Work effectively as a members or leader of a health care team or other professional group |
| PBLI2 | Identify strengths, deficiencies, and limits in one’s knowledge and expertise |
| PBLI3 | Identify and perform appropriate learning activities to guide personal and professional development |
| **PBLI4 (PBLI3)** | Systematically analyze practice using quality improvement methods with the goal of practice improvement |
| **PBLI5(PBLI4)** | Incorporate formative evaluation feedback into daily practice |
| PBLI6 | Locate, appraise, and assimilate evidence from scientific studies related to their patient’s health problems |
| PBLI8 | Develop the necessary skills to be an effective teacher |
| PBLI9 | Participate in the education of patients, families, students, residents, and other health professionals |
| PBLI10 | Take primary responsibility for lifelong learning to improve knowledge, skills, and practice performance through familiarity with general and experience-specific goals and objectives and attendance at conferences |
| **PROF2(Prof1)** | Show responsiveness to patient needs that supersedes self-interest |
| **PROF4(Prof2)** | Demonstrate a sense of duty and accountability to patients, society, and the profession |
| PPD6 | Provide leadership that enhances team functioning, the learning environment, and/or health care system/environment with the ultimate intent of improving care of patients |
| **PPD8(Prof6)** | Recognize that ambiguity is part of clinical medicine and respond by utilizing appropriate resources in dealing with uncertainty |

References:

1. The Pediatrics Milestones Project. A Joint Initiative of the American Board of Pediatrics and the Accreditation Council for Graduate Medical Education. Available at: <https://www.abp.org/sites/abp/files/pdf/milestones.pdf>. Accessed June 5, 2016.
2. Hoffman B et al. The community health and advocacy milestones profile: A novel tool linking community pediatrics and advocacy training to assessment of milestones-based competence in pediatric residency training. View from the Association of Pediatric Program Directors. *Academic Pediatrics* 2016;16(4):309-313.
