# Supplementary material for: The Community Pediatrics Training Initiative Project Planning Tool: A Practical Approach to Community-Based Advocacy
Source: MedEdPORTAL. 2017 Sep 18;13:10630. doi: 10.15766/mep_2374-8265.10630 (PMC6338167; doi:10.15766/mep_2374-8265.10630)
Supplement: Supplementary file 1 — A. CHAMP.pdf B. CHAMP Mapping Tool.pdf C. AAP CPTI Project Planning Tool.docx D. AAP CPTI Project Planning Tool Milestones-Based Assessment Rubric.docx E. Project Planning Tool Users Guide.pptx [file mep-13-10630-s001.zip › E. Project Planning Tool Users Guide.pptx]

## Slide 1
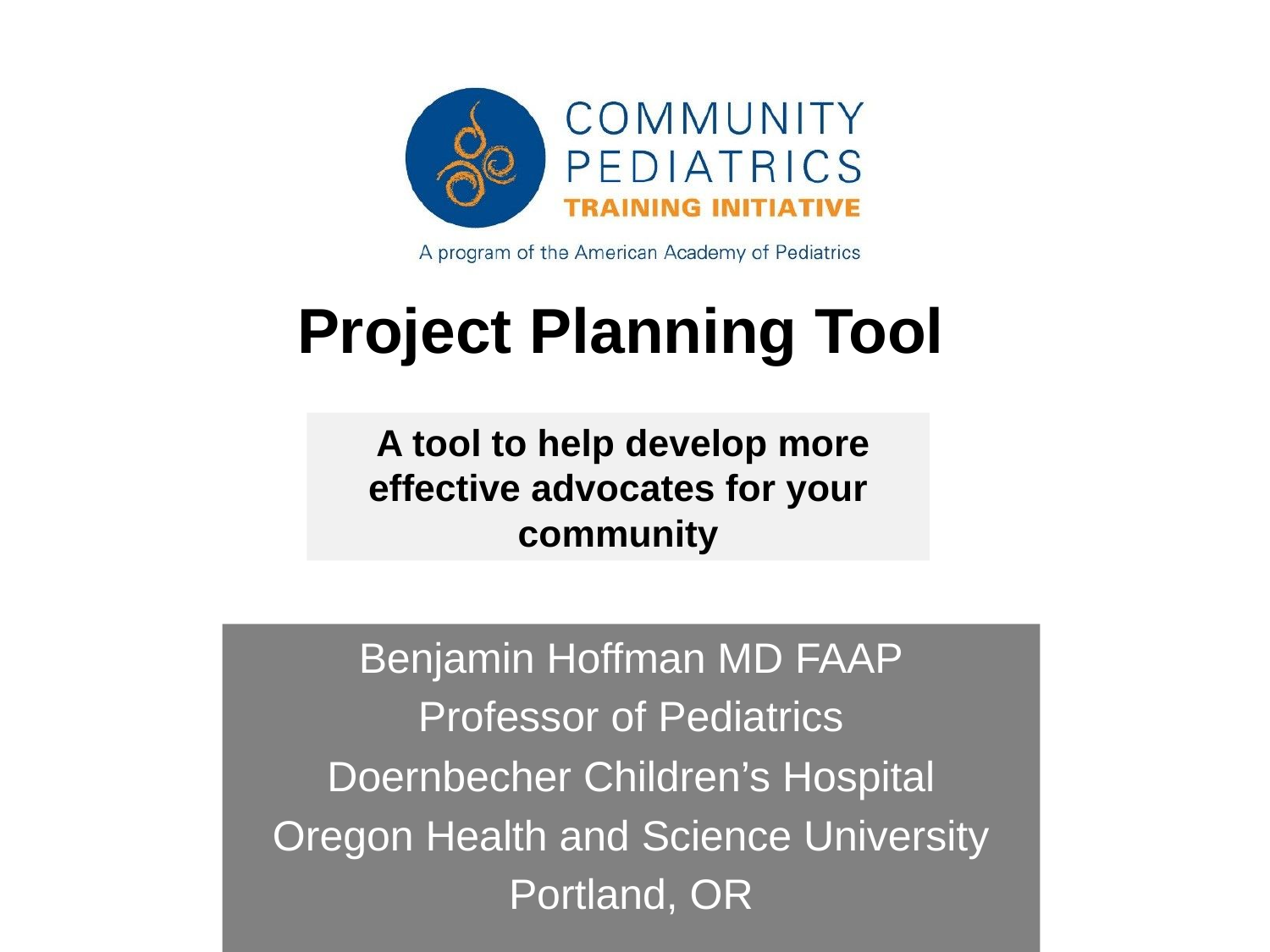

Project Planning Tool
# A tool to help develop more effective advocates for your community
Benjamin Hoffman MD FAAP
Professor of Pediatrics
Doernbecher Children’s Hospital
Oregon Health and Science University
Portland, OR

## Slide 2
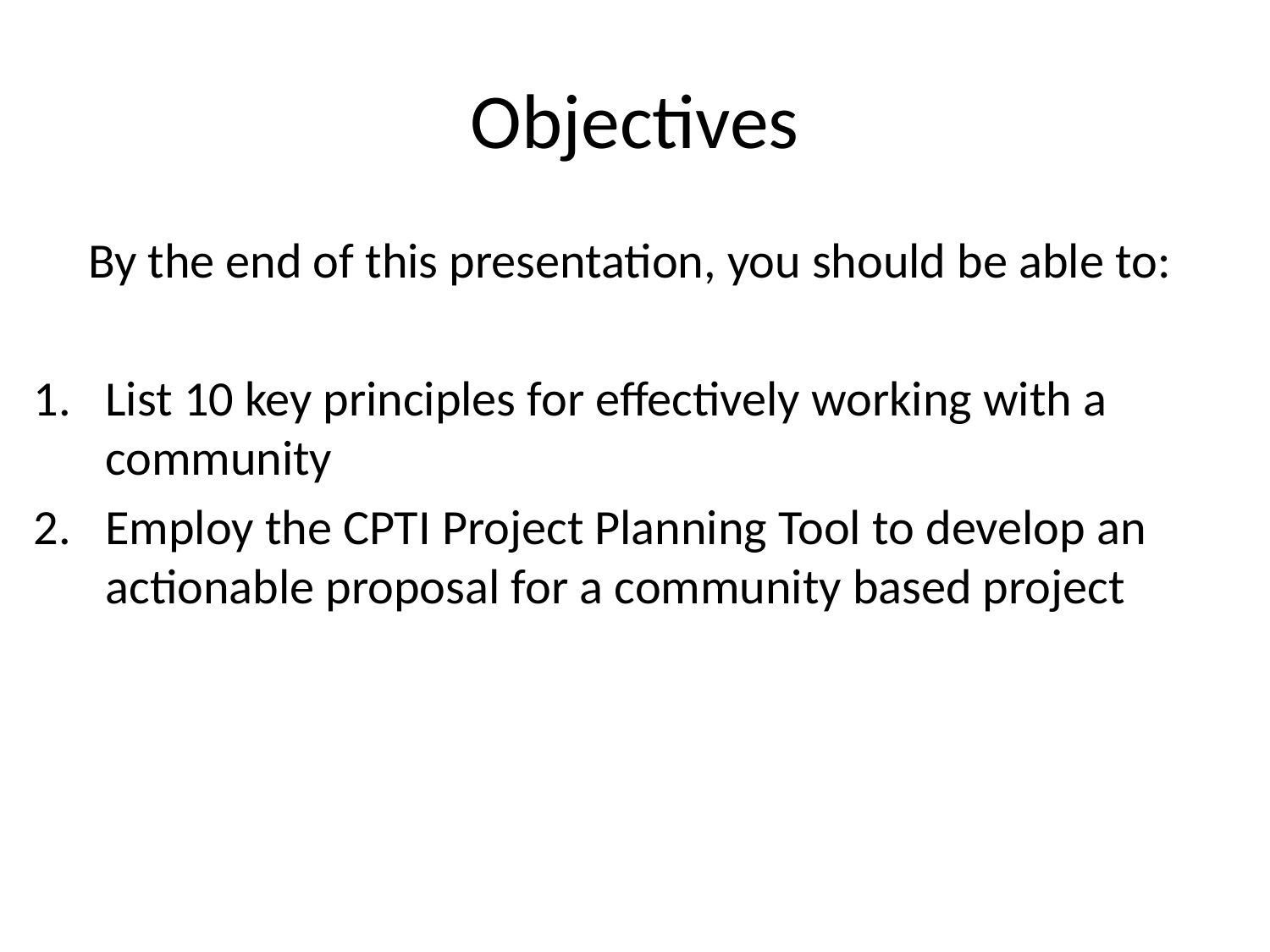

# Objectives
By the end of this presentation, you should be able to:
List 10 key principles for effectively working with a community
Employ the CPTI Project Planning Tool to develop an actionable proposal for a community based project

## Slide 3
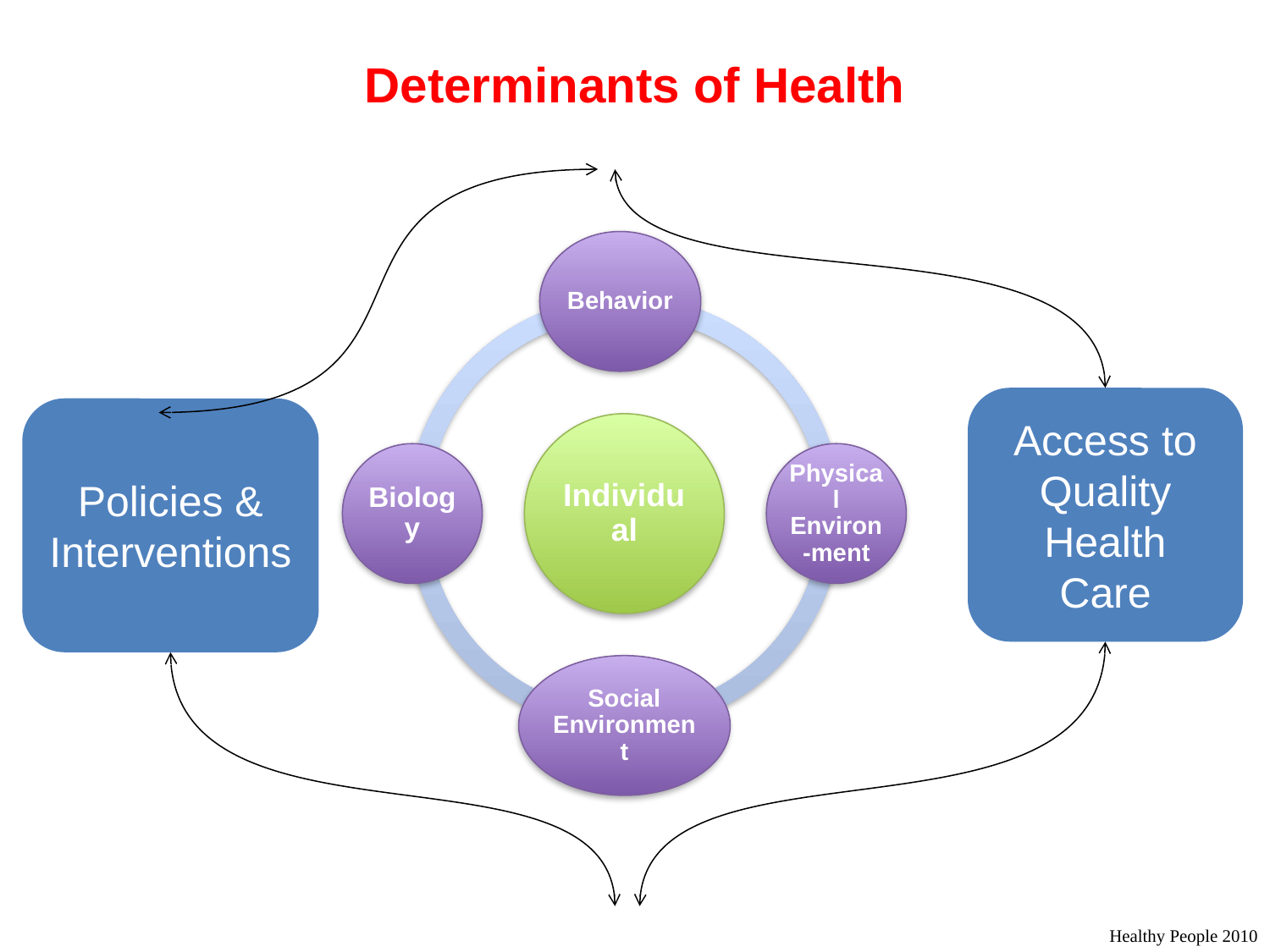

Determinants of Health
Access to Quality Health Care
Policies & Interventions
Healthy People 2010

## Slide 4
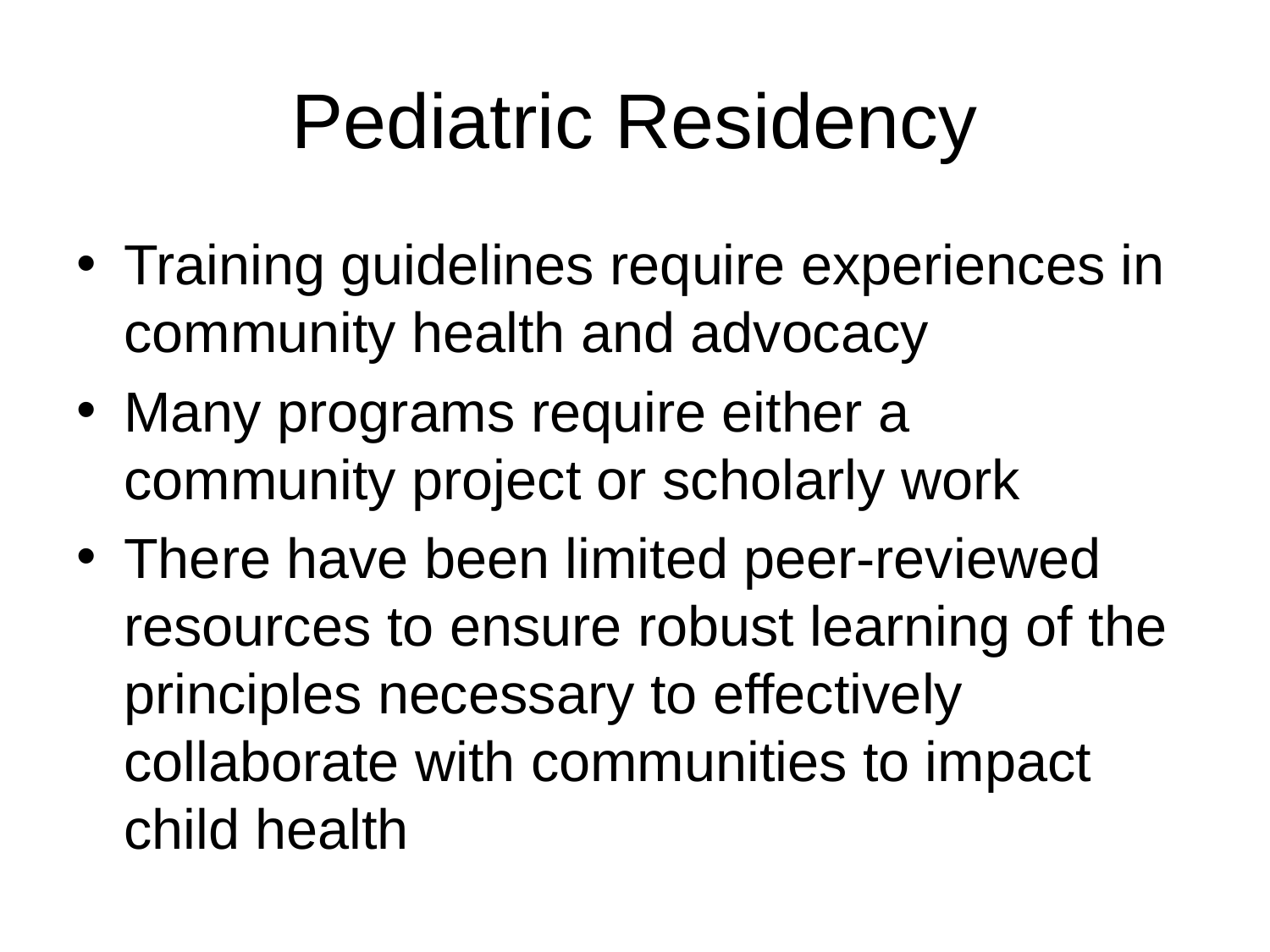

# Pediatric Residency
Training guidelines require experiences in community health and advocacy
Many programs require either a community project or scholarly work
There have been limited peer-reviewed resources to ensure robust learning of the principles necessary to effectively collaborate with communities to impact child health

## Slide 5
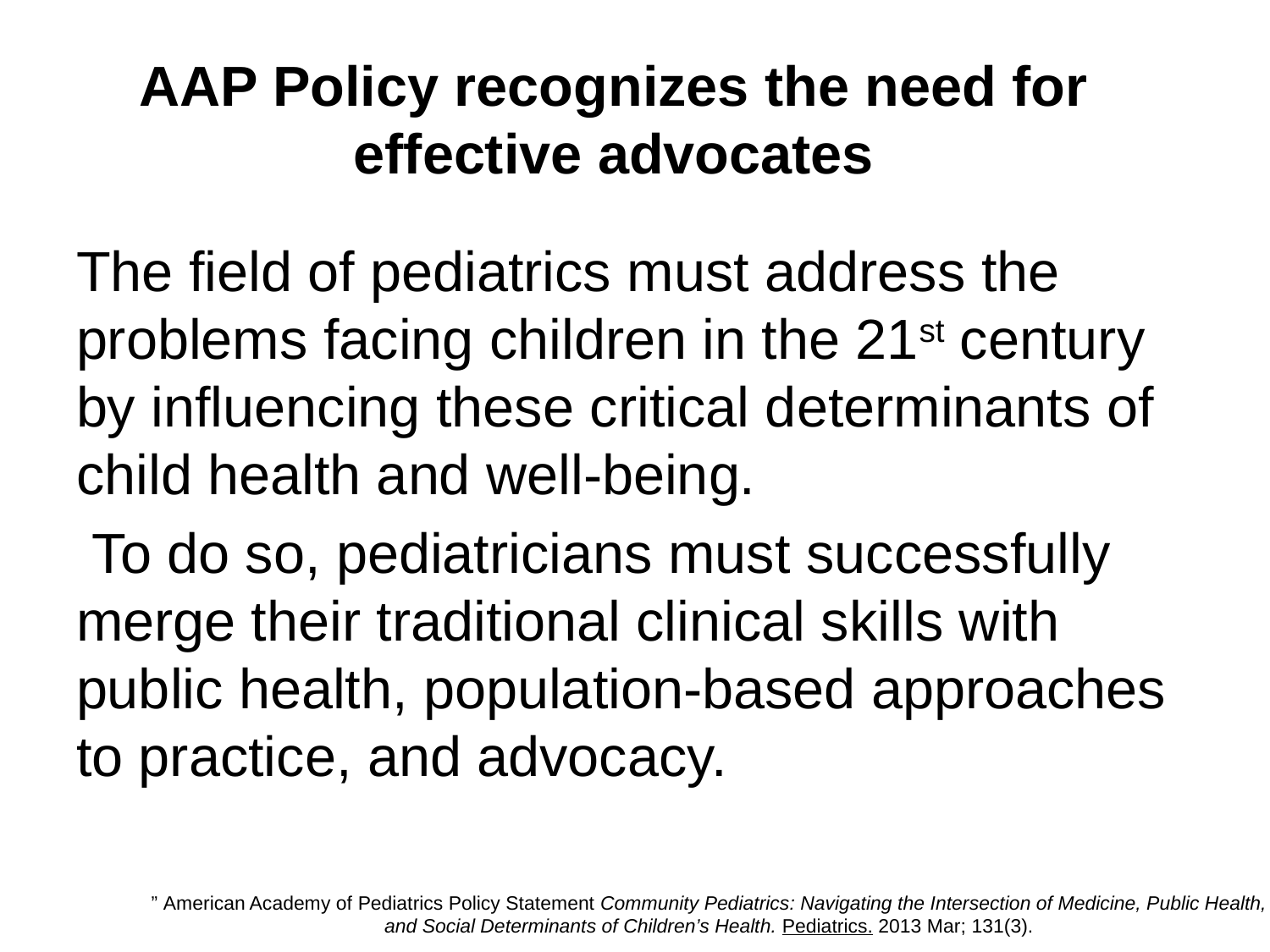

# AAP Policy recognizes the need for effective advocates
The field of pediatrics must address the problems facing children in the 21st century by influencing these critical determinants of child health and well-being.
 To do so, pediatricians must successfully merge their traditional clinical skills with public health, population-based approaches to practice, and advocacy.
” American Academy of Pediatrics Policy Statement Community Pediatrics: Navigating the Intersection of Medicine, Public Health, and Social Determinants of Children’s Health. Pediatrics. 2013 Mar; 131(3).

## Slide 6
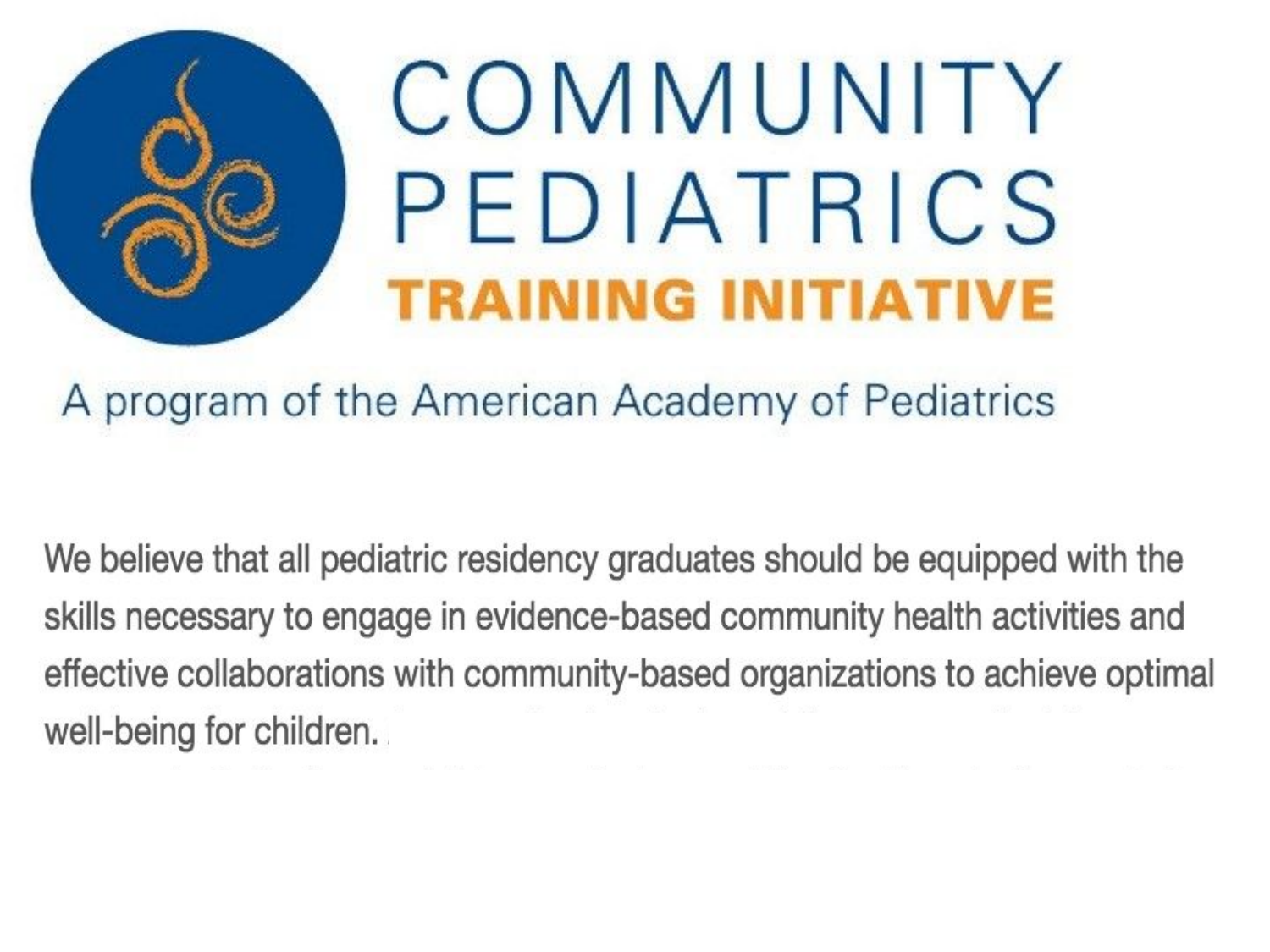

## Slide 7
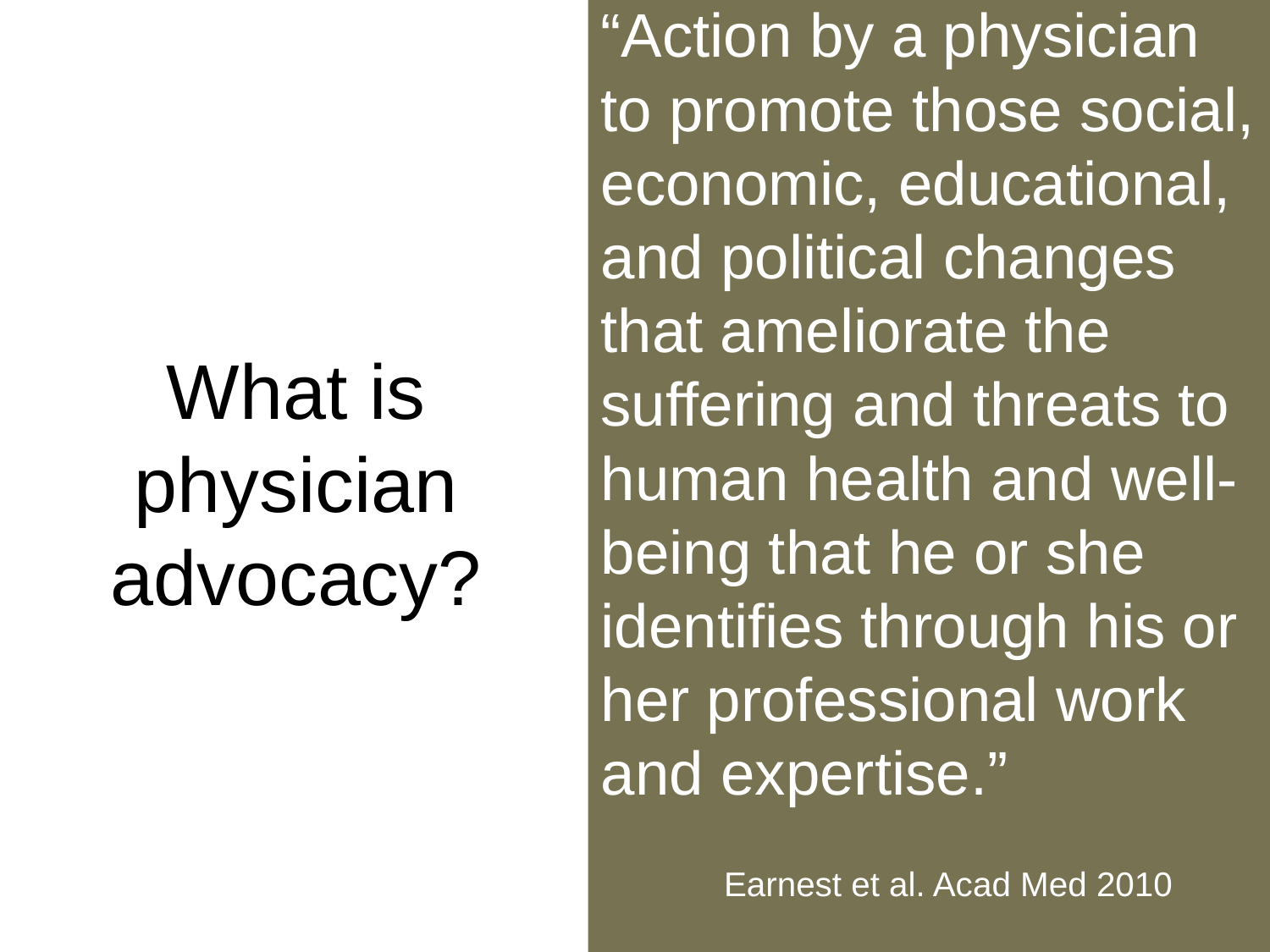

“Action by a physician to promote those social, economic, educational, and political changes that ameliorate the suffering and threats to human health and well-being that he or she identifies through his or her professional work and expertise.”
	Earnest et al. Acad Med 2010
# What is physician advocacy?

## Slide 8
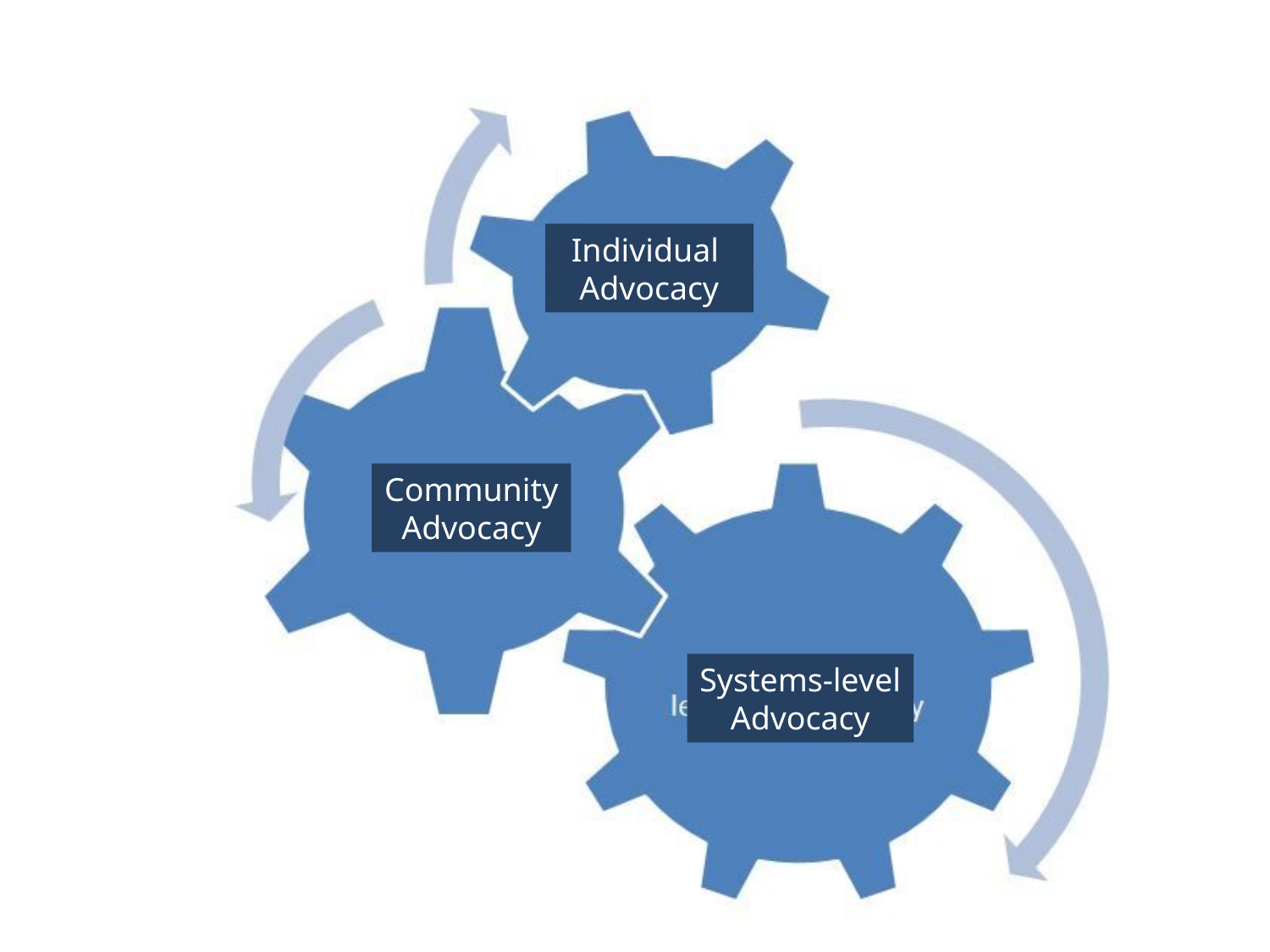

Individual
Advocacy
Community
Advocacy
Systems-level
Advocacy

## Slide 9
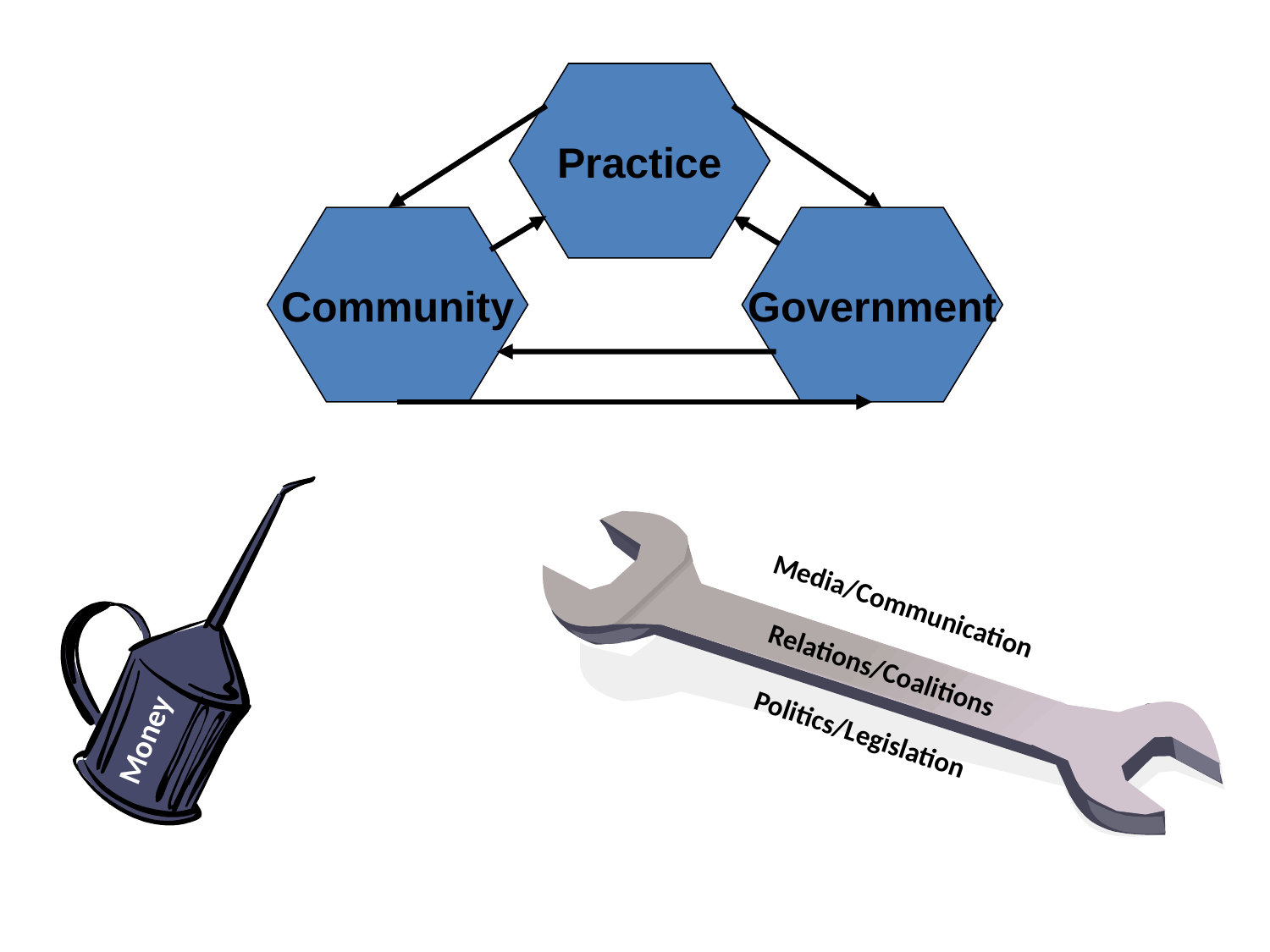

Practice
Community
Government
Media/Communication
Relations/Coalitions
Politics/Legislation
Money

## Slide 10
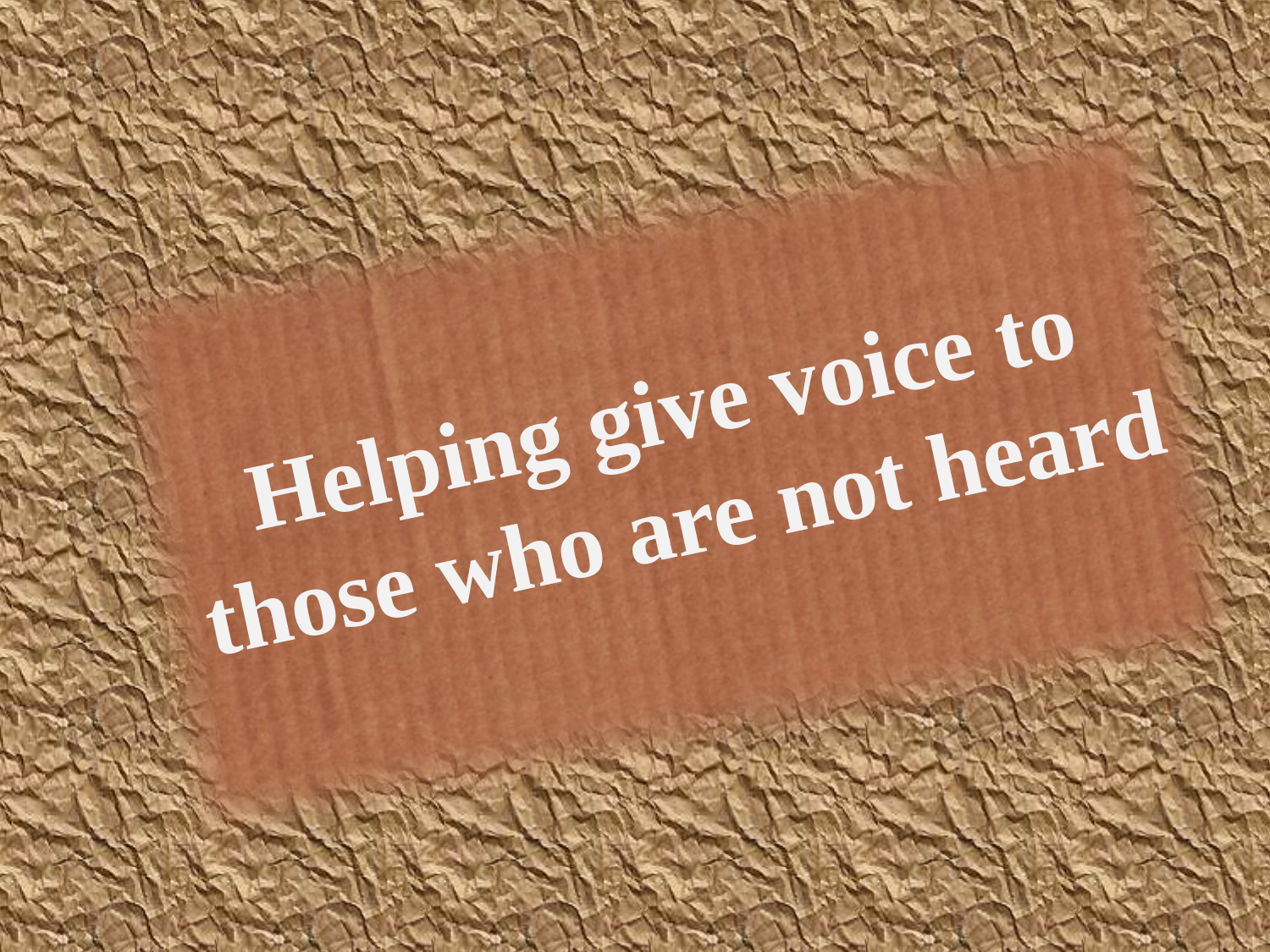

# Helping give voice to those who are not heard

## Slide 11
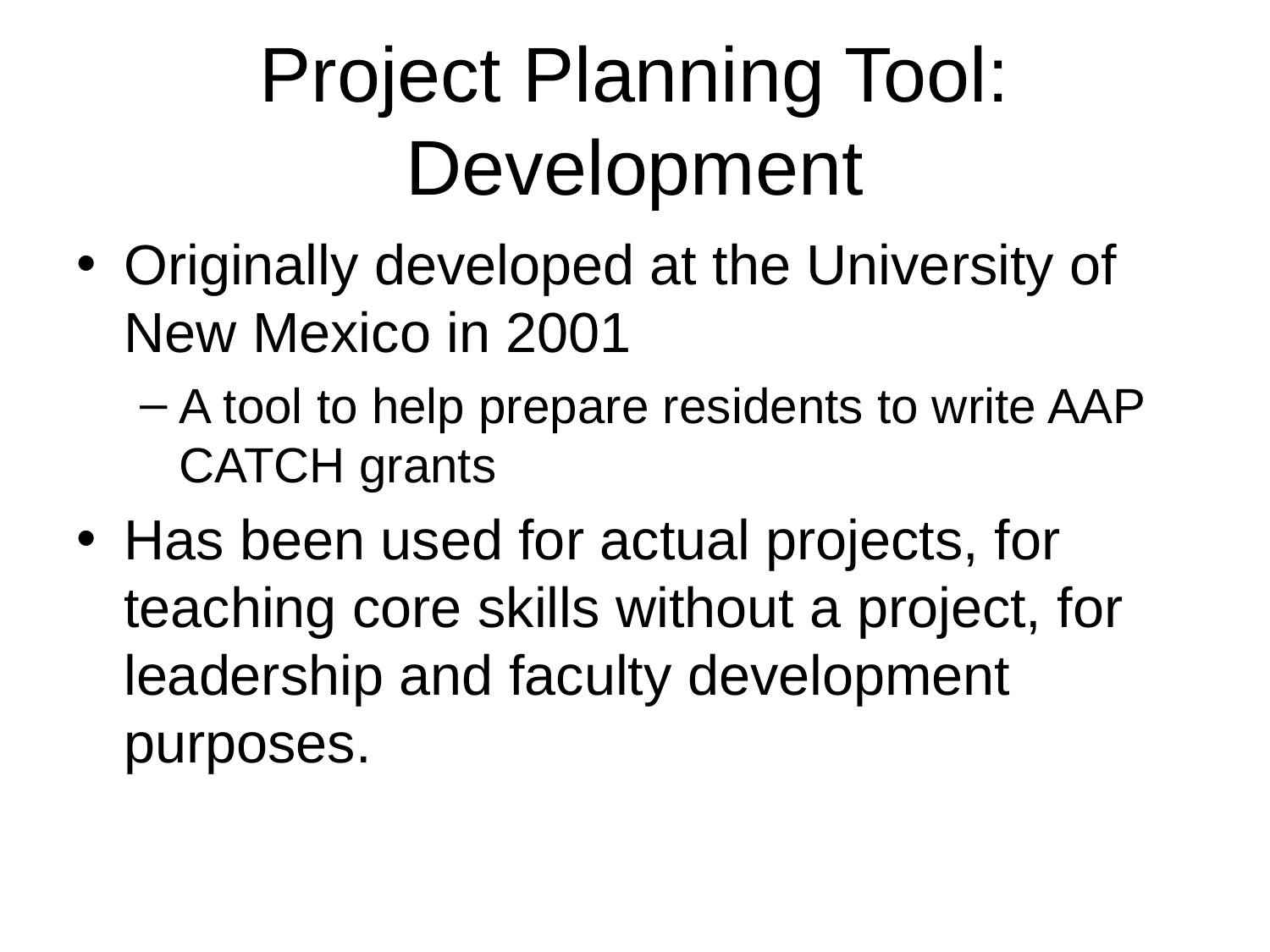

# Project Planning Tool: Development
Originally developed at the University of New Mexico in 2001
A tool to help prepare residents to write AAP CATCH grants
Has been used for actual projects, for teaching core skills without a project, for leadership and faculty development purposes.

## Slide 12
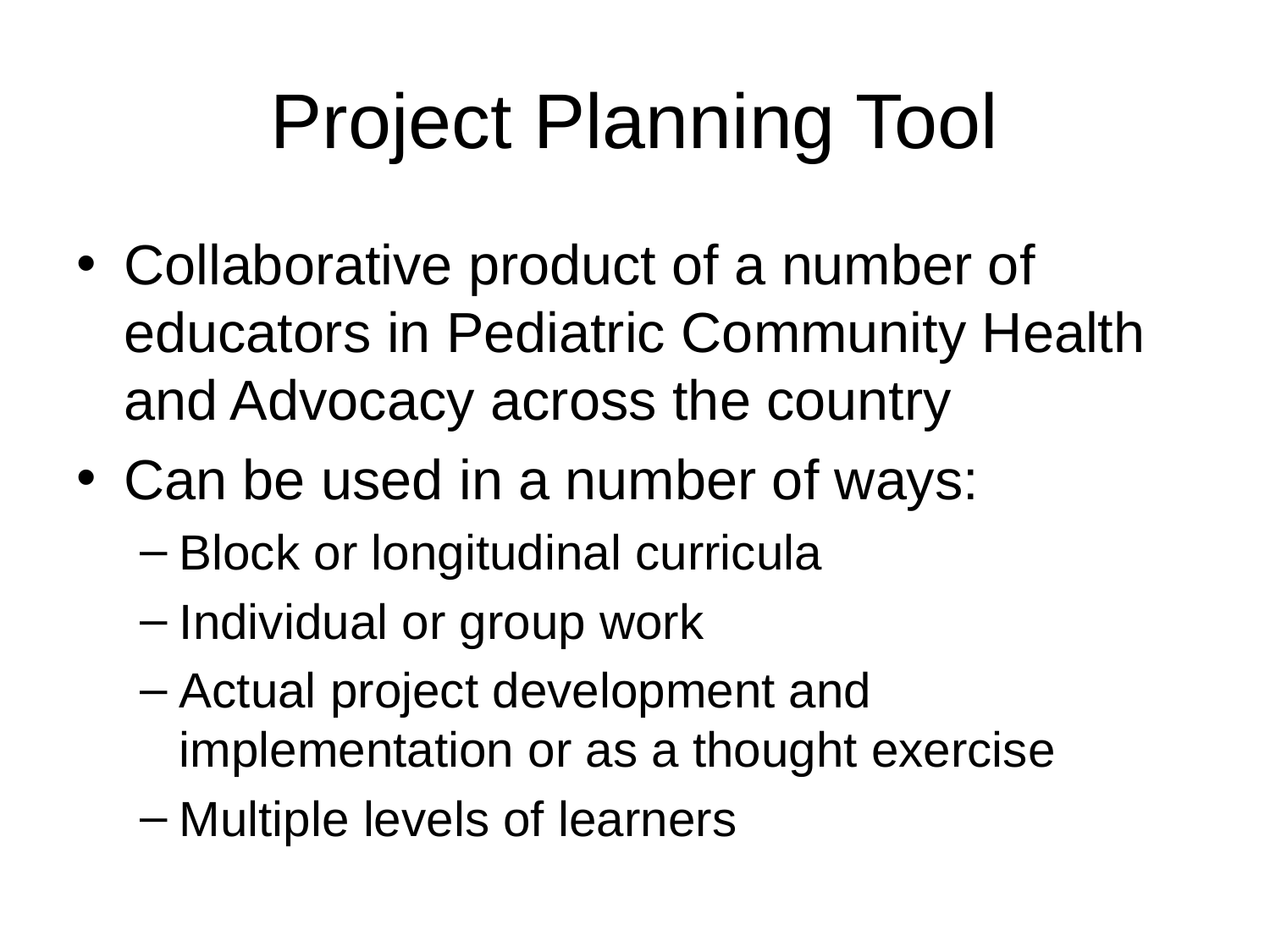

# Project Planning Tool
Collaborative product of a number of educators in Pediatric Community Health and Advocacy across the country
Can be used in a number of ways:
Block or longitudinal curricula
Individual or group work
Actual project development and implementation or as a thought exercise
Multiple levels of learners

## Slide 13
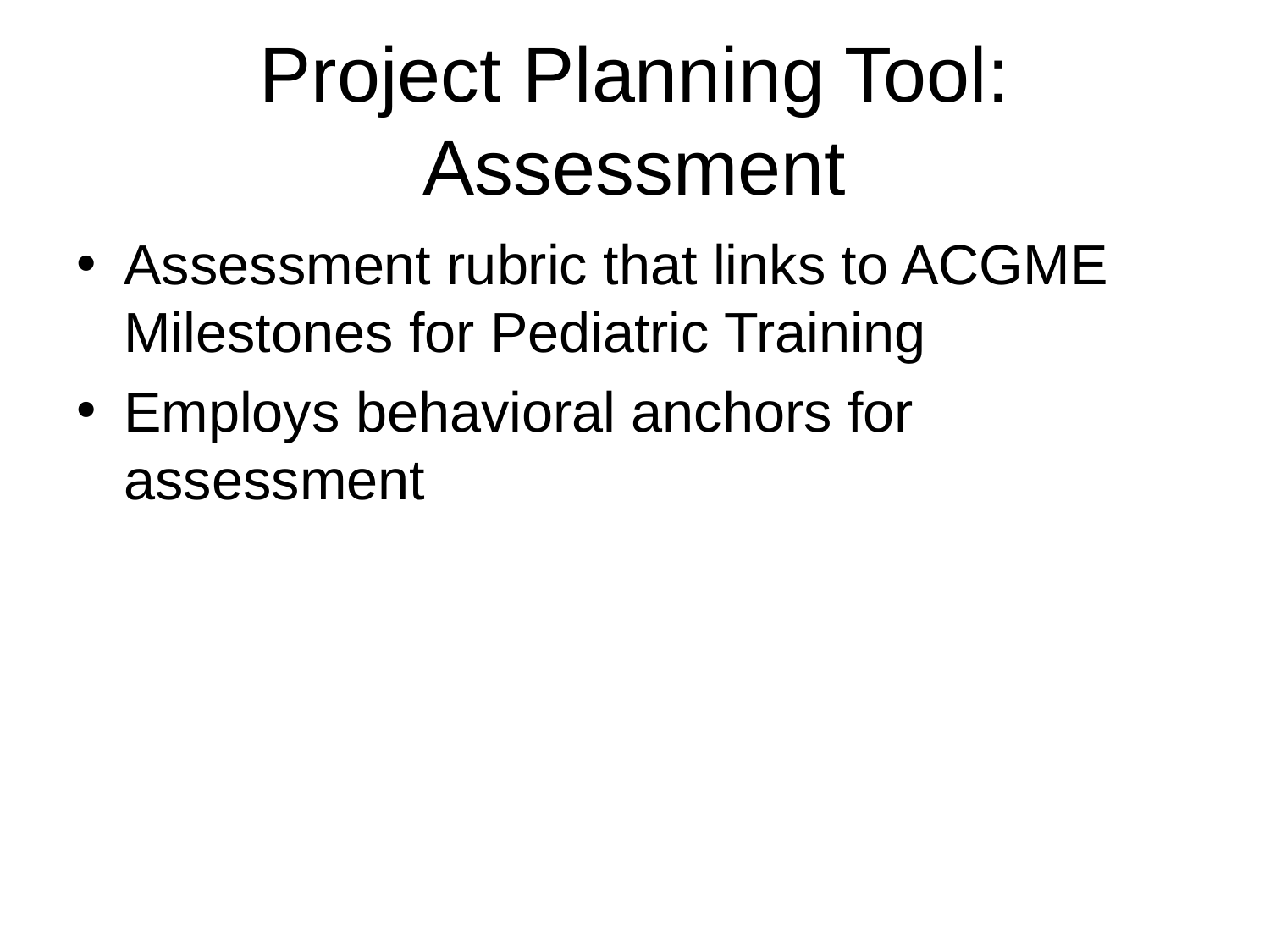

# Project Planning Tool: Assessment
Assessment rubric that links to ACGME Milestones for Pediatric Training
Employs behavioral anchors for assessment

## Slide 14
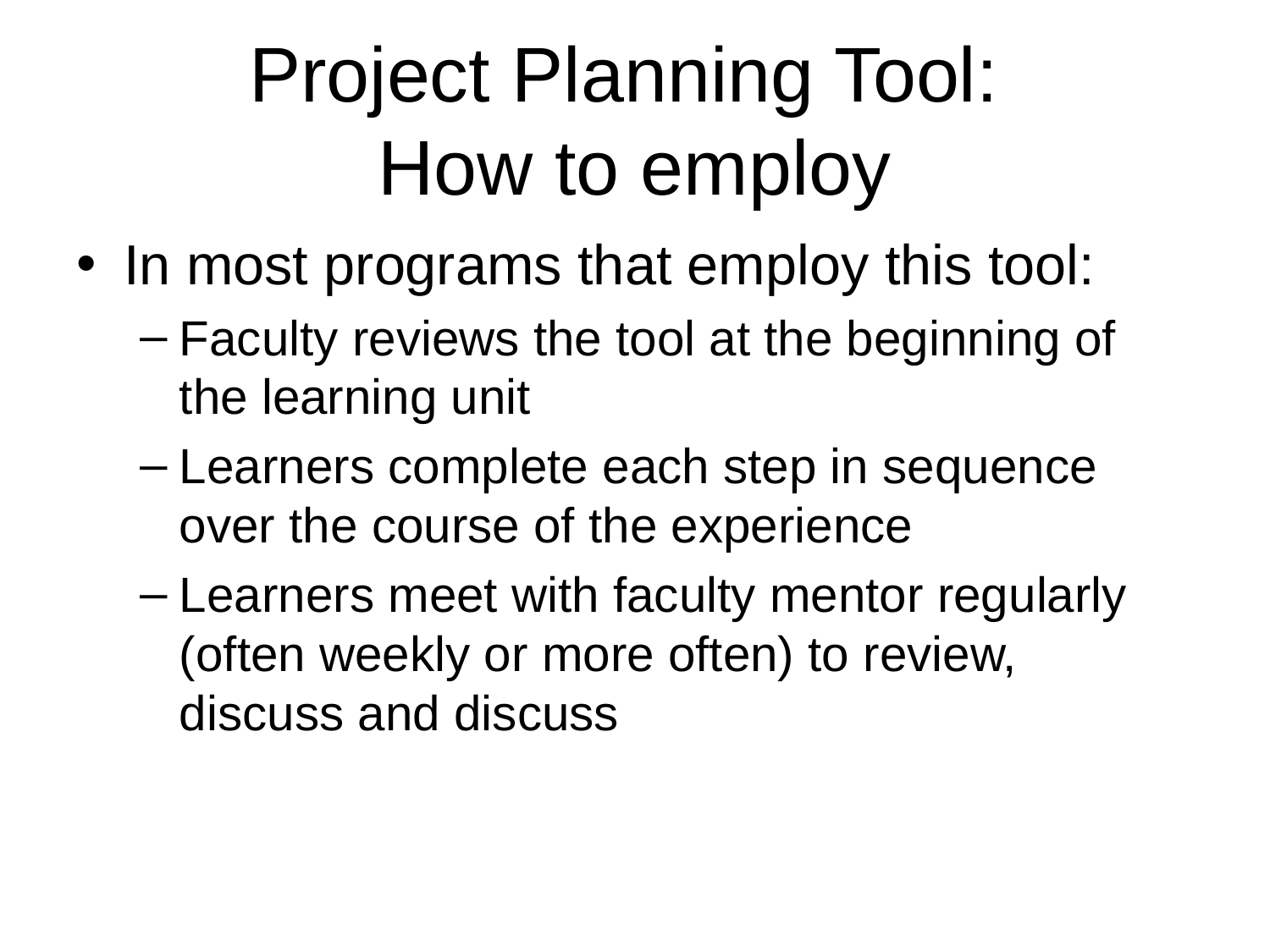

# Project Planning Tool: How to employ
In most programs that employ this tool:
Faculty reviews the tool at the beginning of the learning unit
Learners complete each step in sequence over the course of the experience
Learners meet with faculty mentor regularly (often weekly or more often) to review, discuss and discuss

## Slide 15
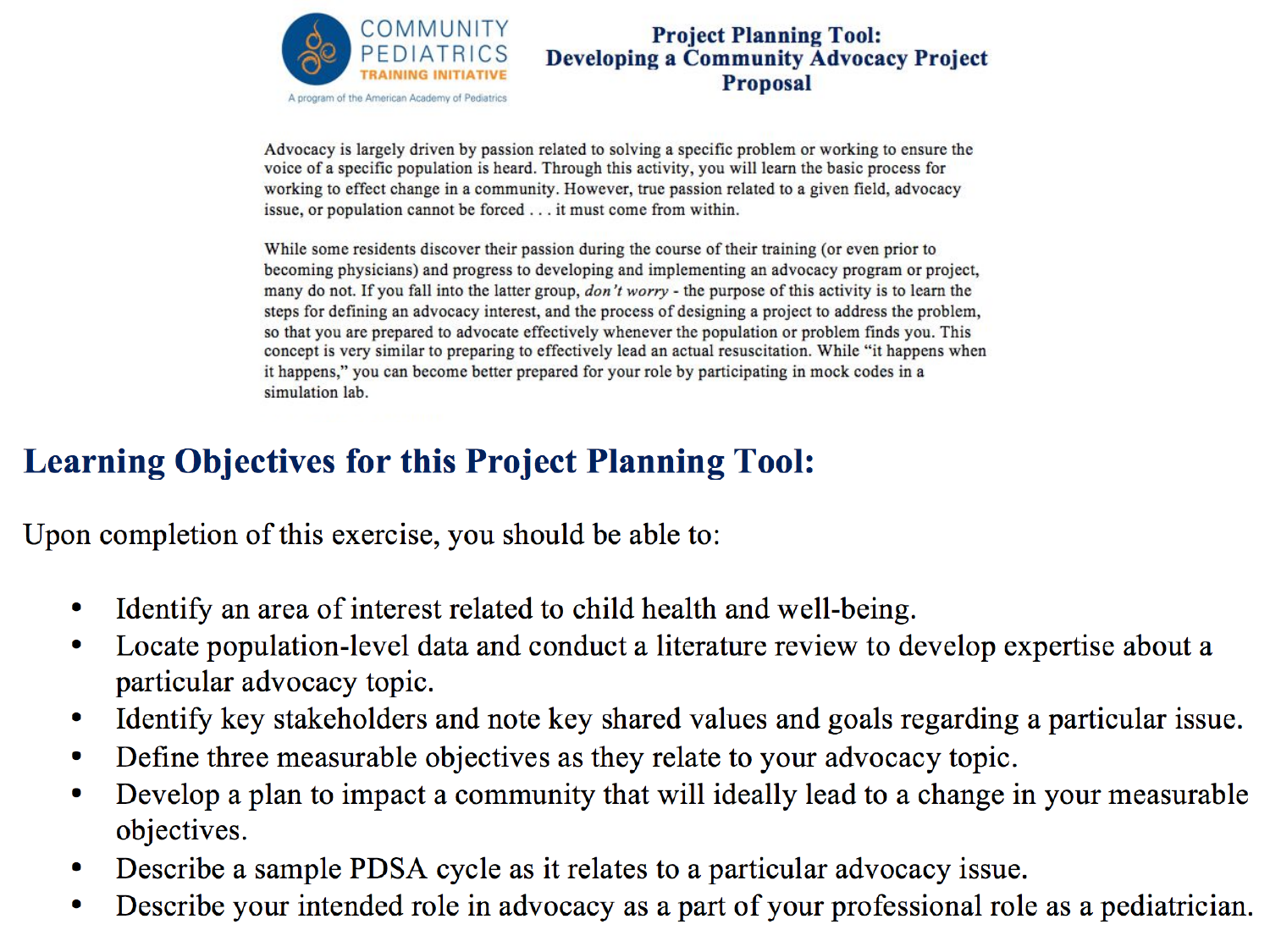

## Slide 16
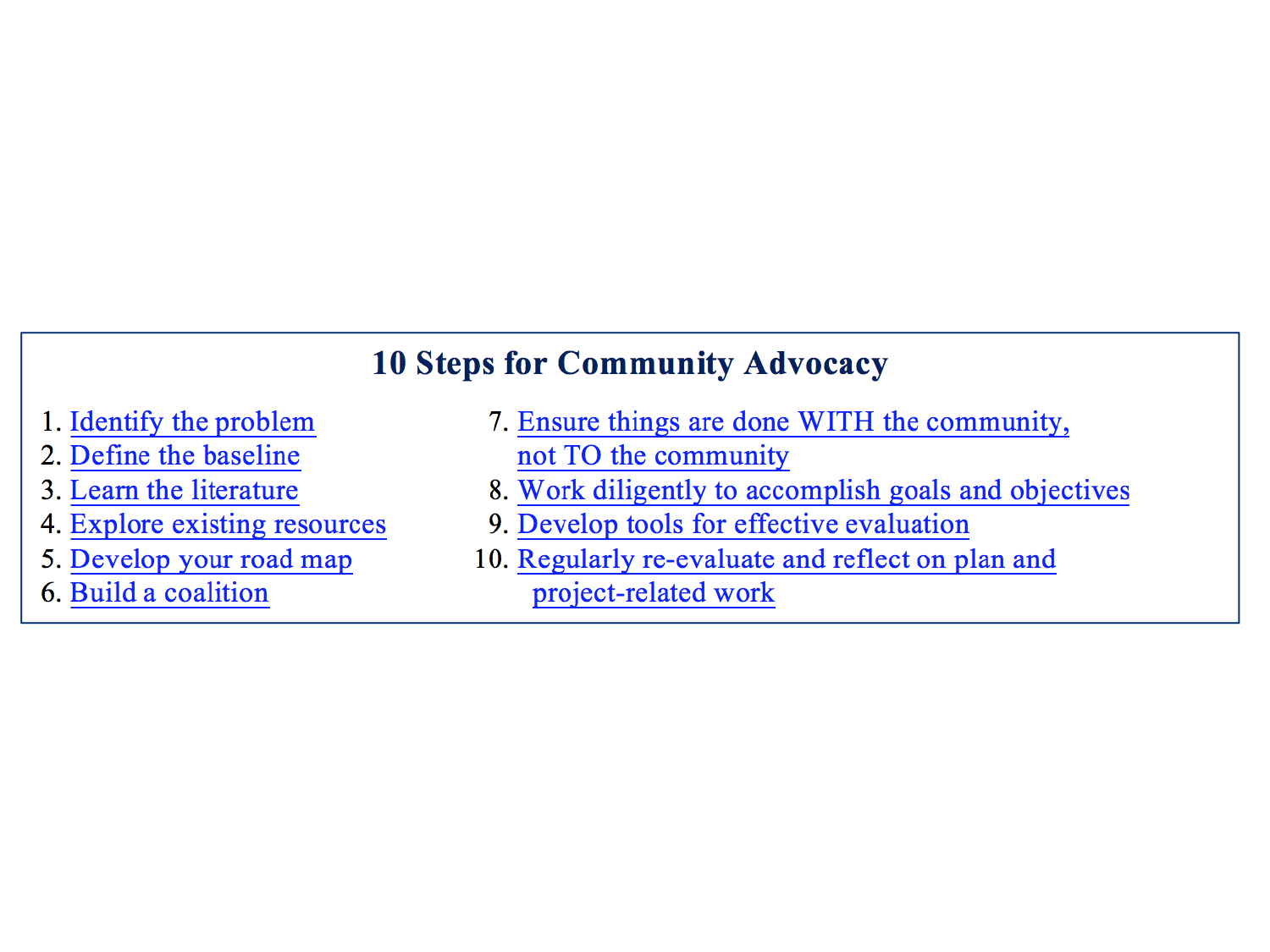

## Slide 17
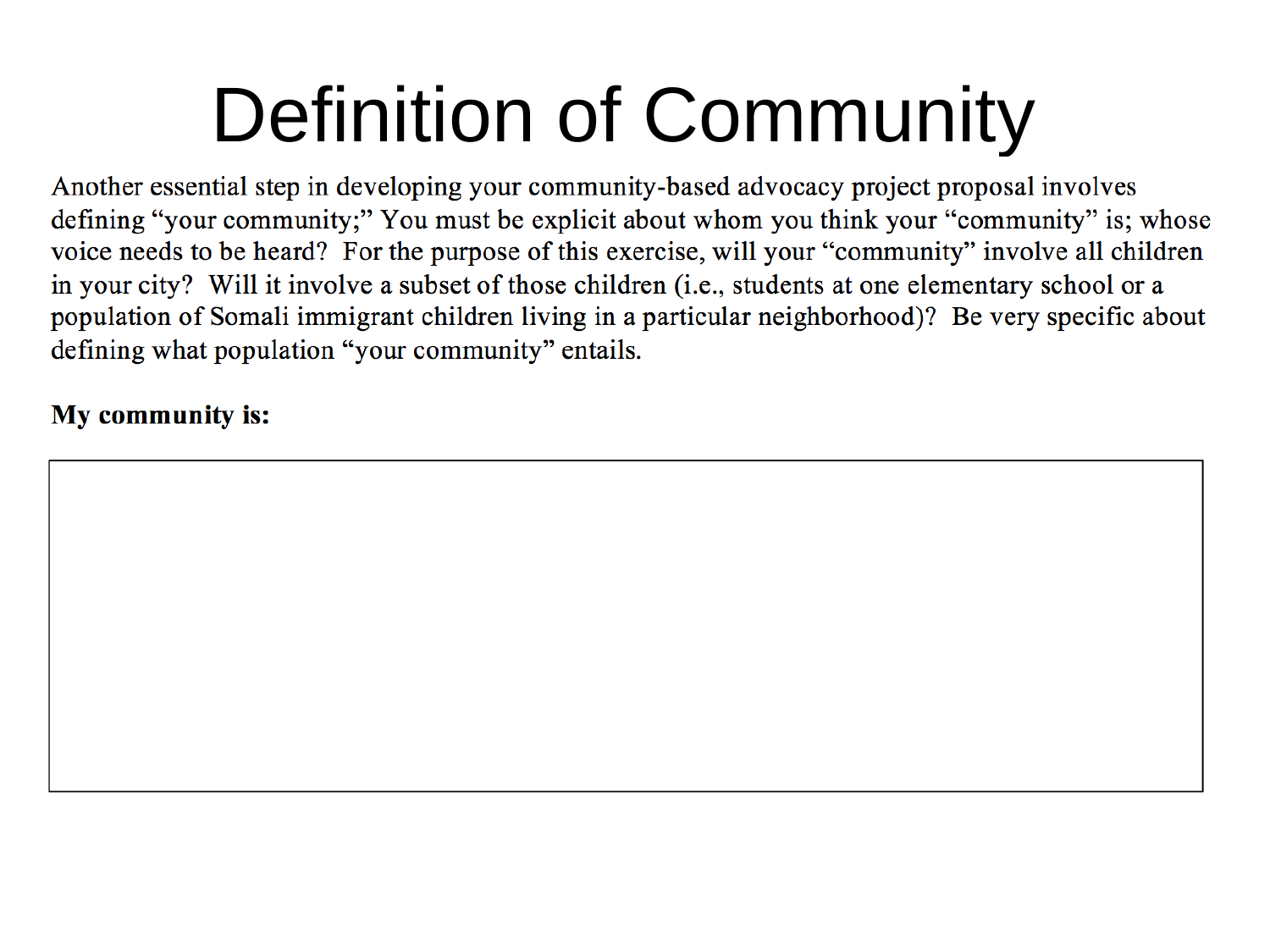

# Definition of Community

## Slide 18
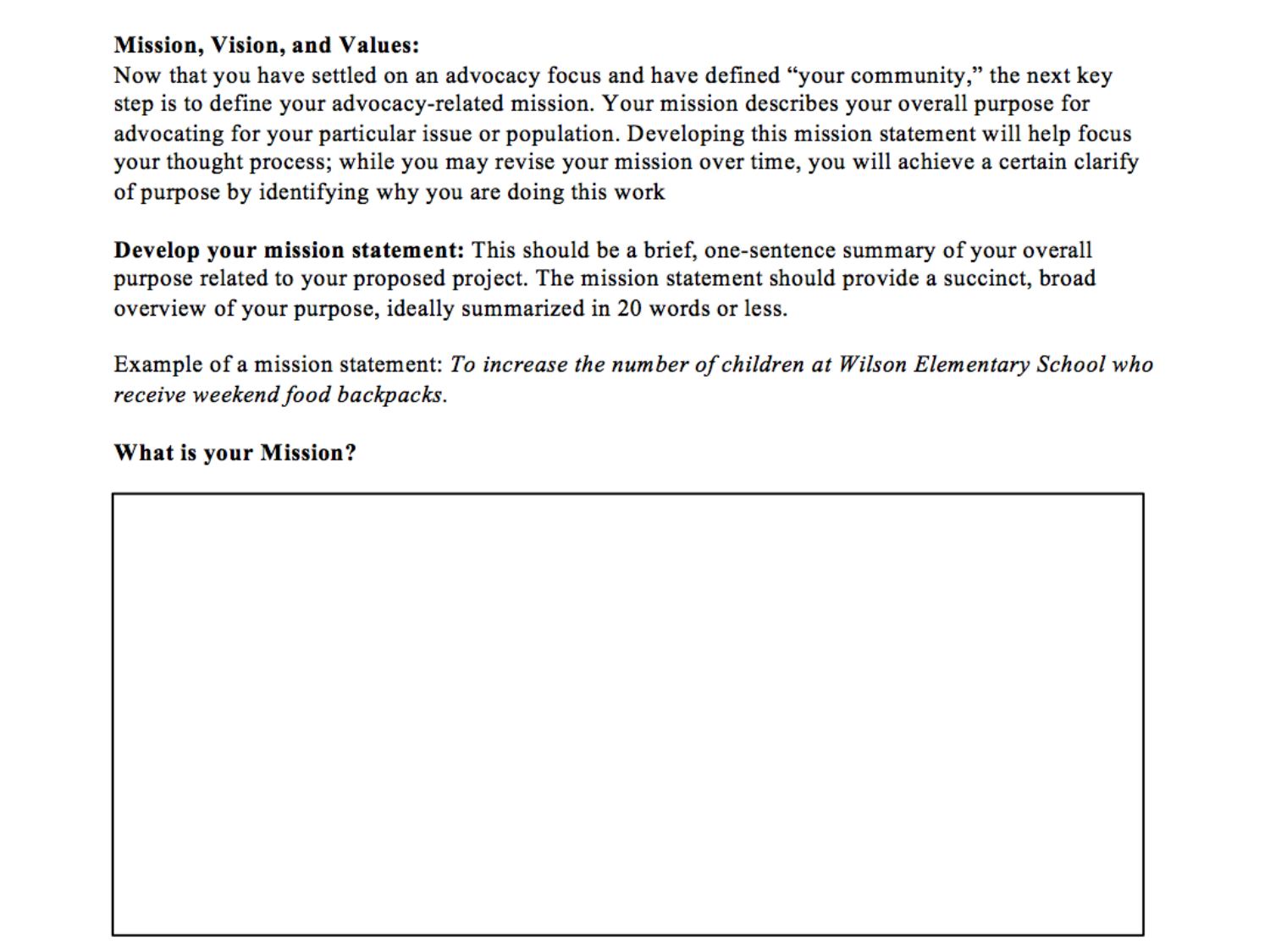

#

## Slide 19
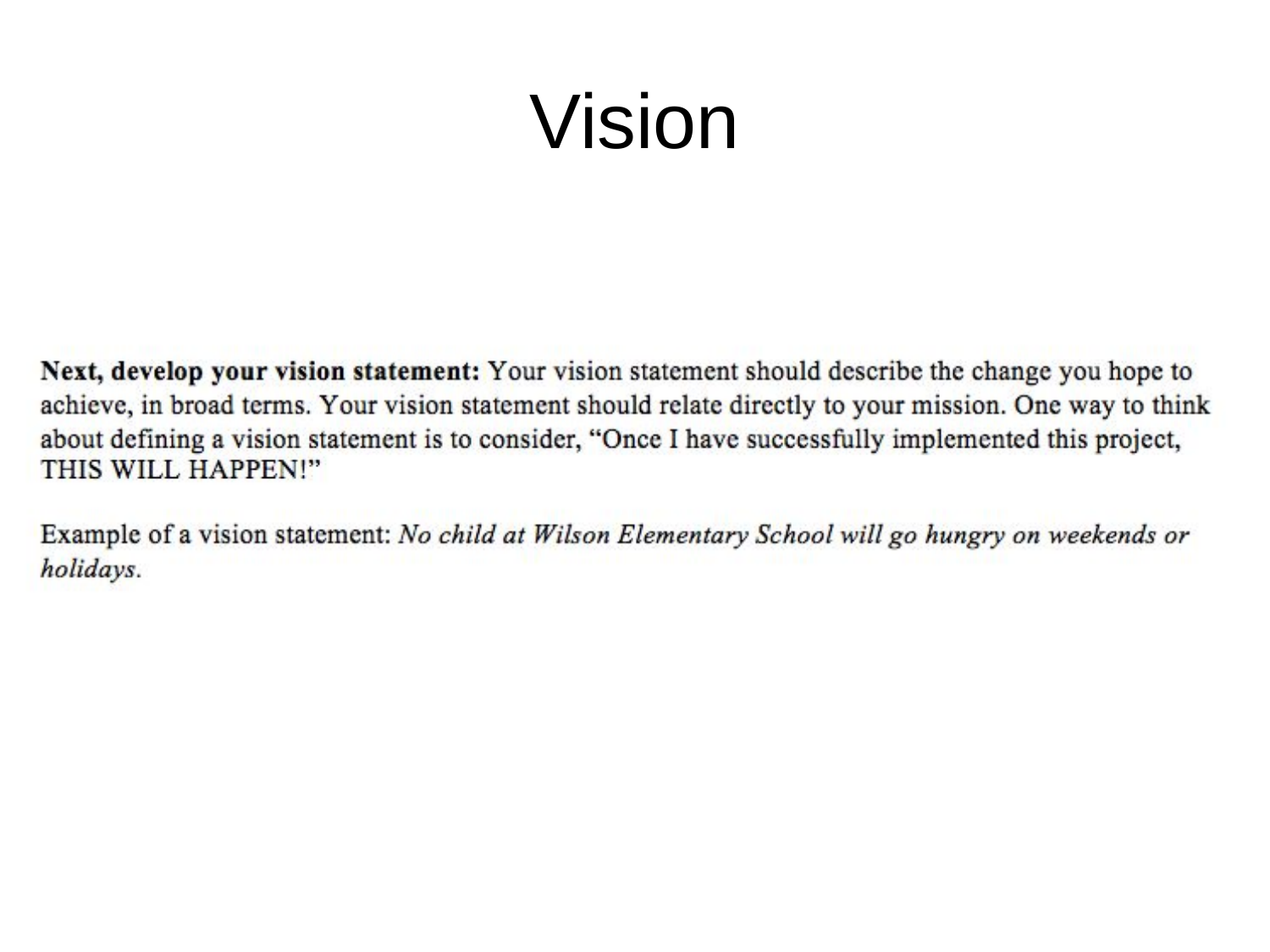

# Vision

## Slide 20
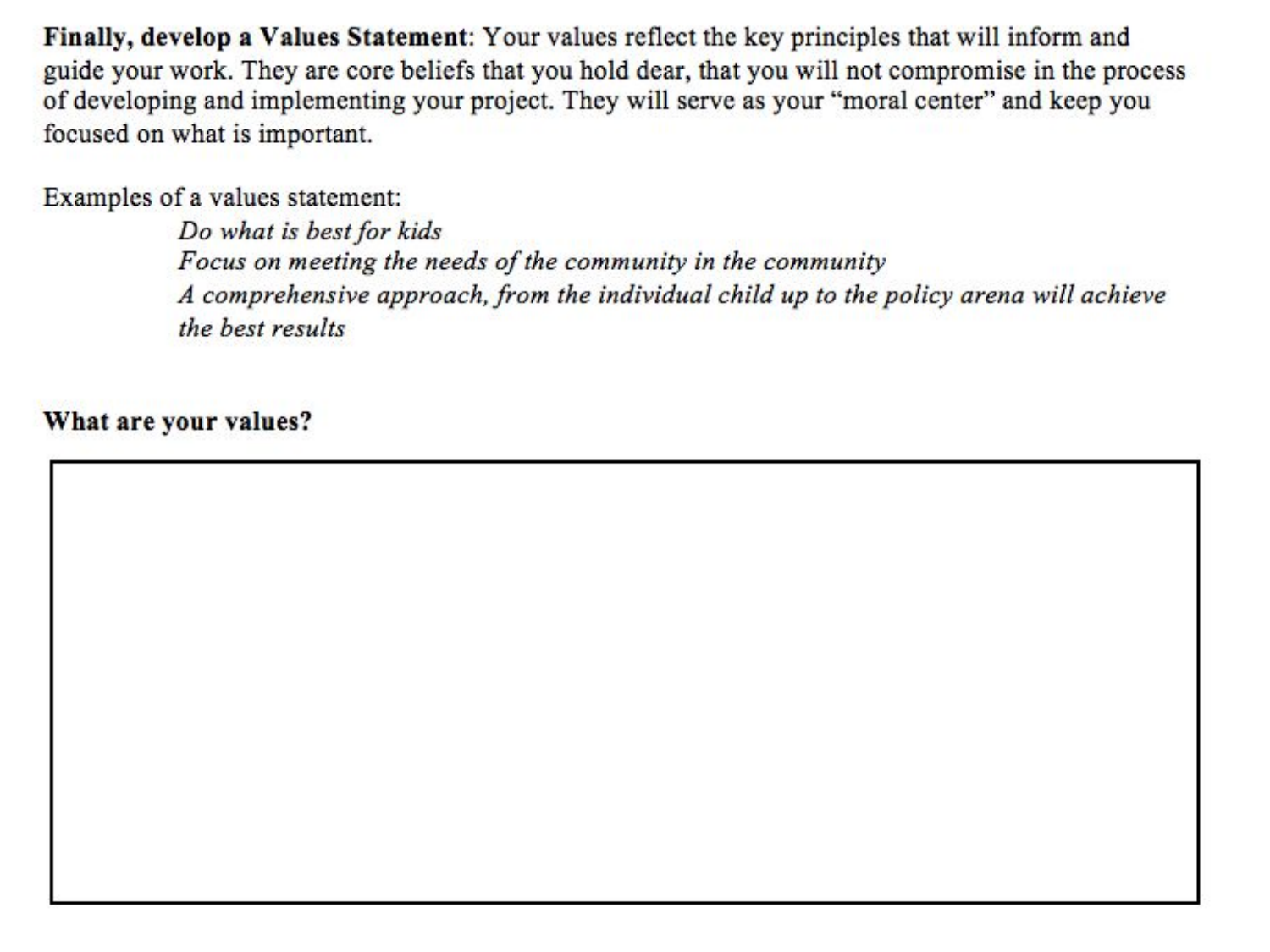

#

## Slide 21
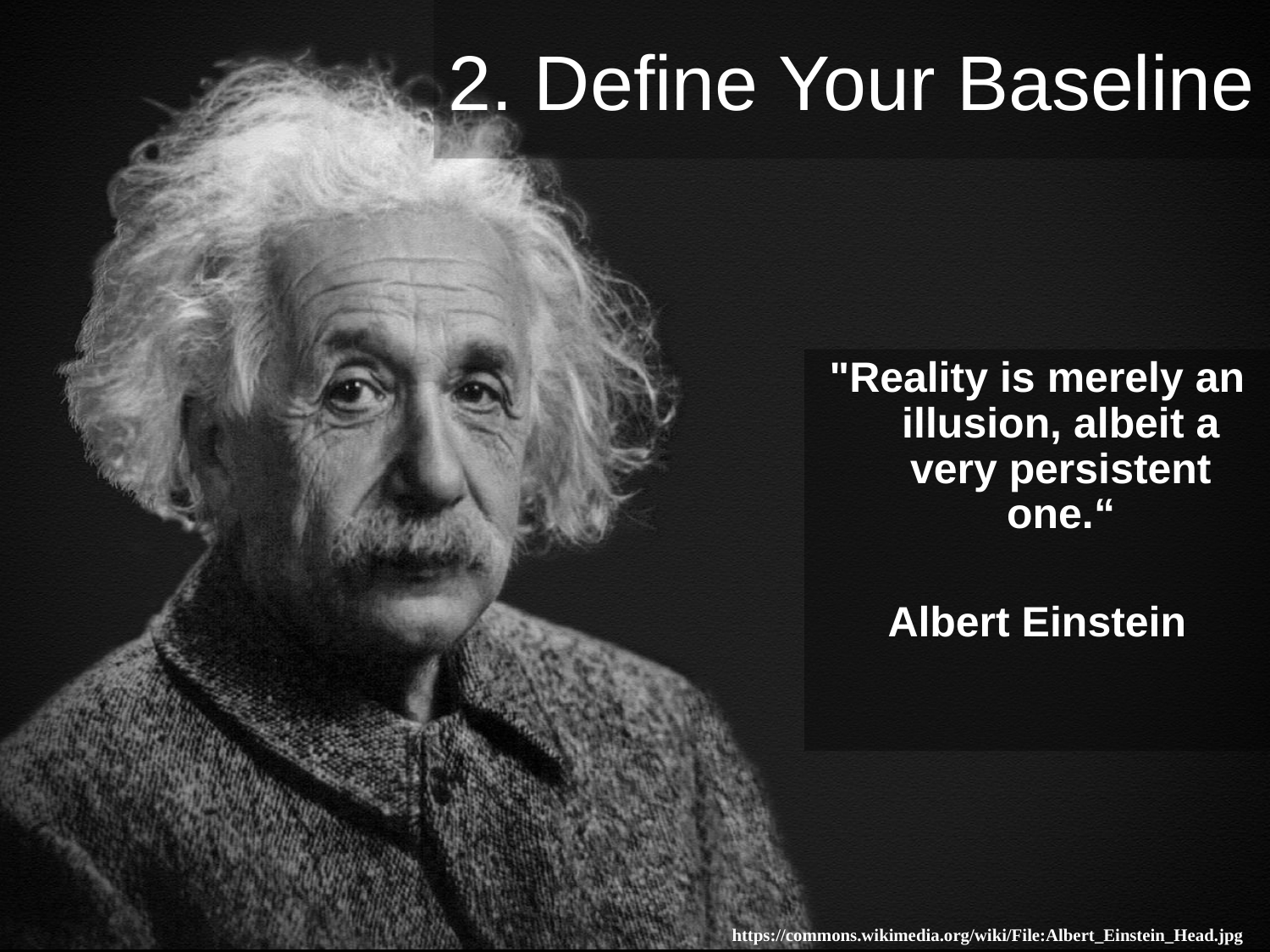

# 2. Define Your Baseline
"Reality is merely an illusion, albeit a very persistent one.“
Albert Einstein
https://commons.wikimedia.org/wiki/File:Albert_Einstein_Head.jpg

## Slide 22
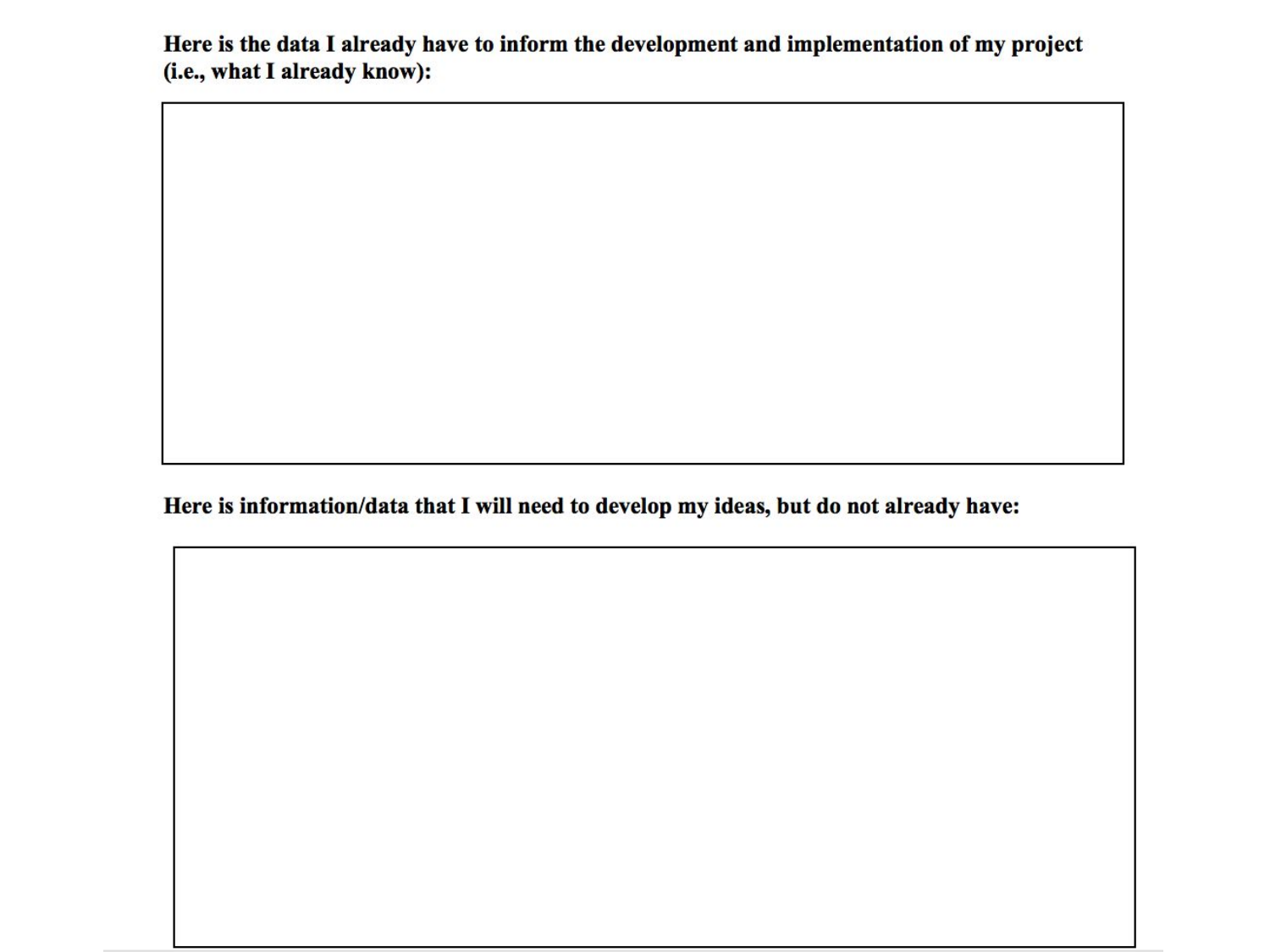

## Slide 23
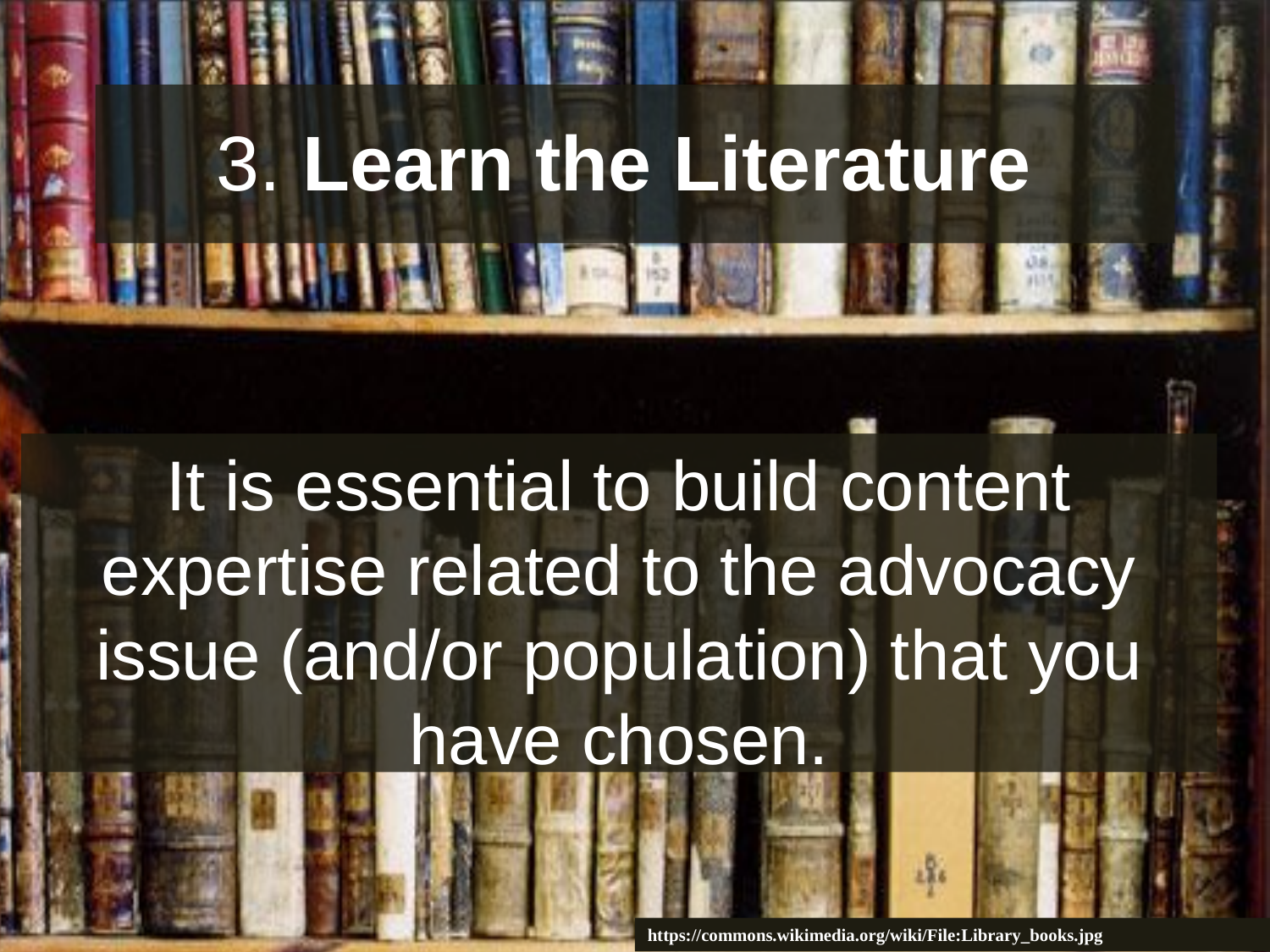

# 3. Learn the Literature
It is essential to build content expertise related to the advocacy issue (and/or population) that you have chosen.
https://commons.wikimedia.org/wiki/File:Library_books.jpg

## Slide 24
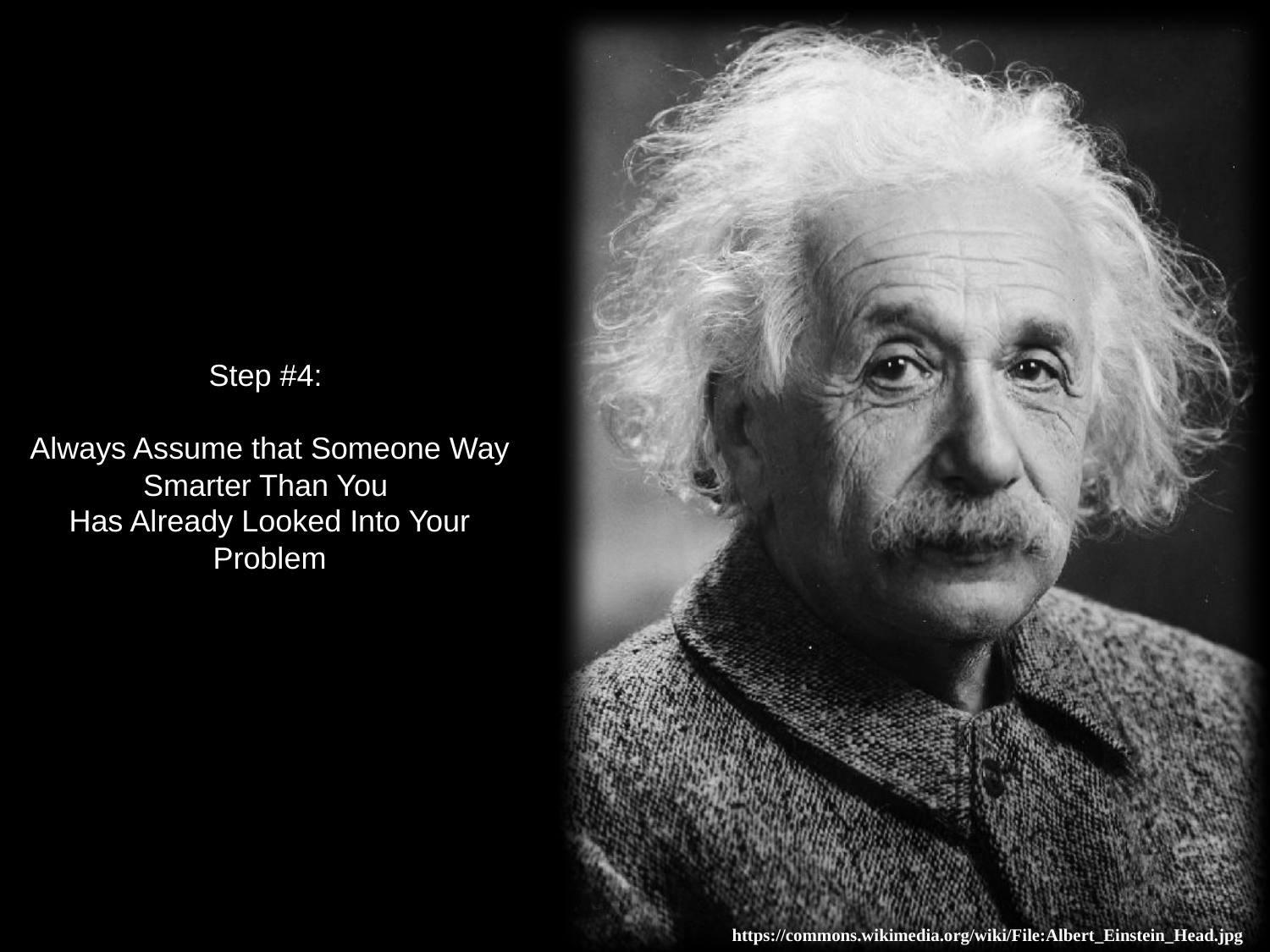

# Step #4: Always Assume that Someone Way Smarter Than You Has Already Looked Into Your Problem
https://commons.wikimedia.org/wiki/File:Albert_Einstein_Head.jpg

## Slide 25
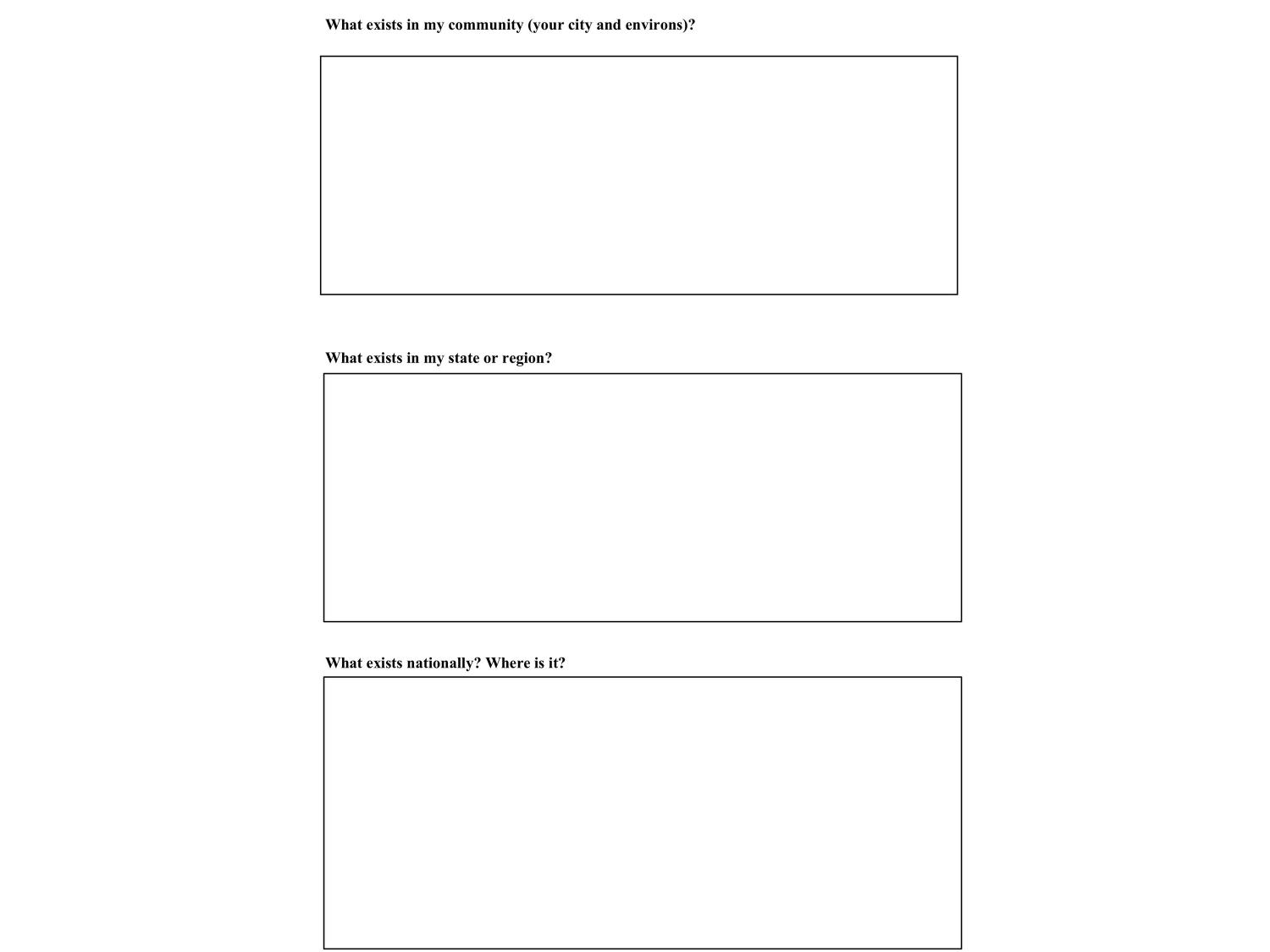

## Slide 26
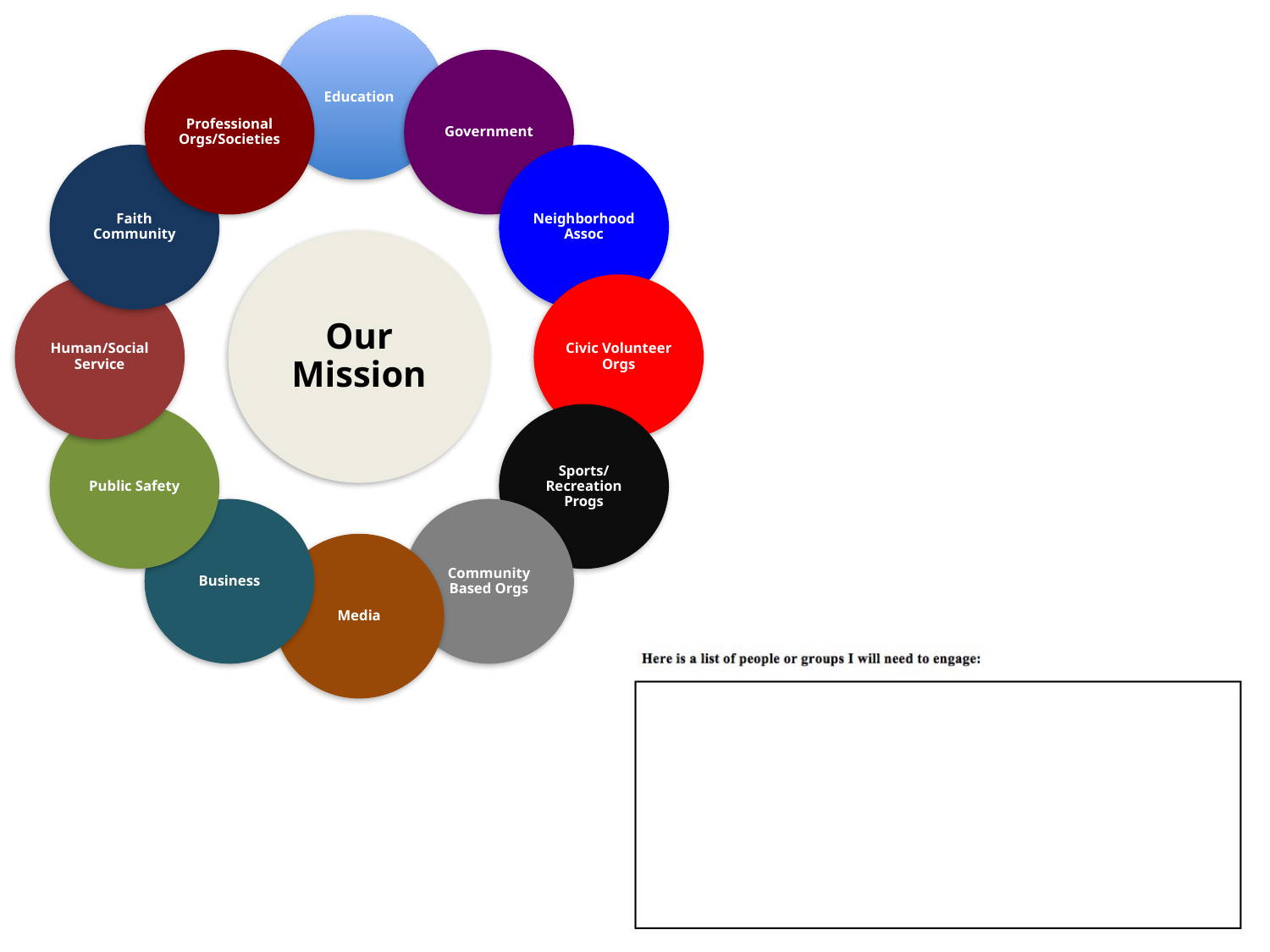

## Slide 27
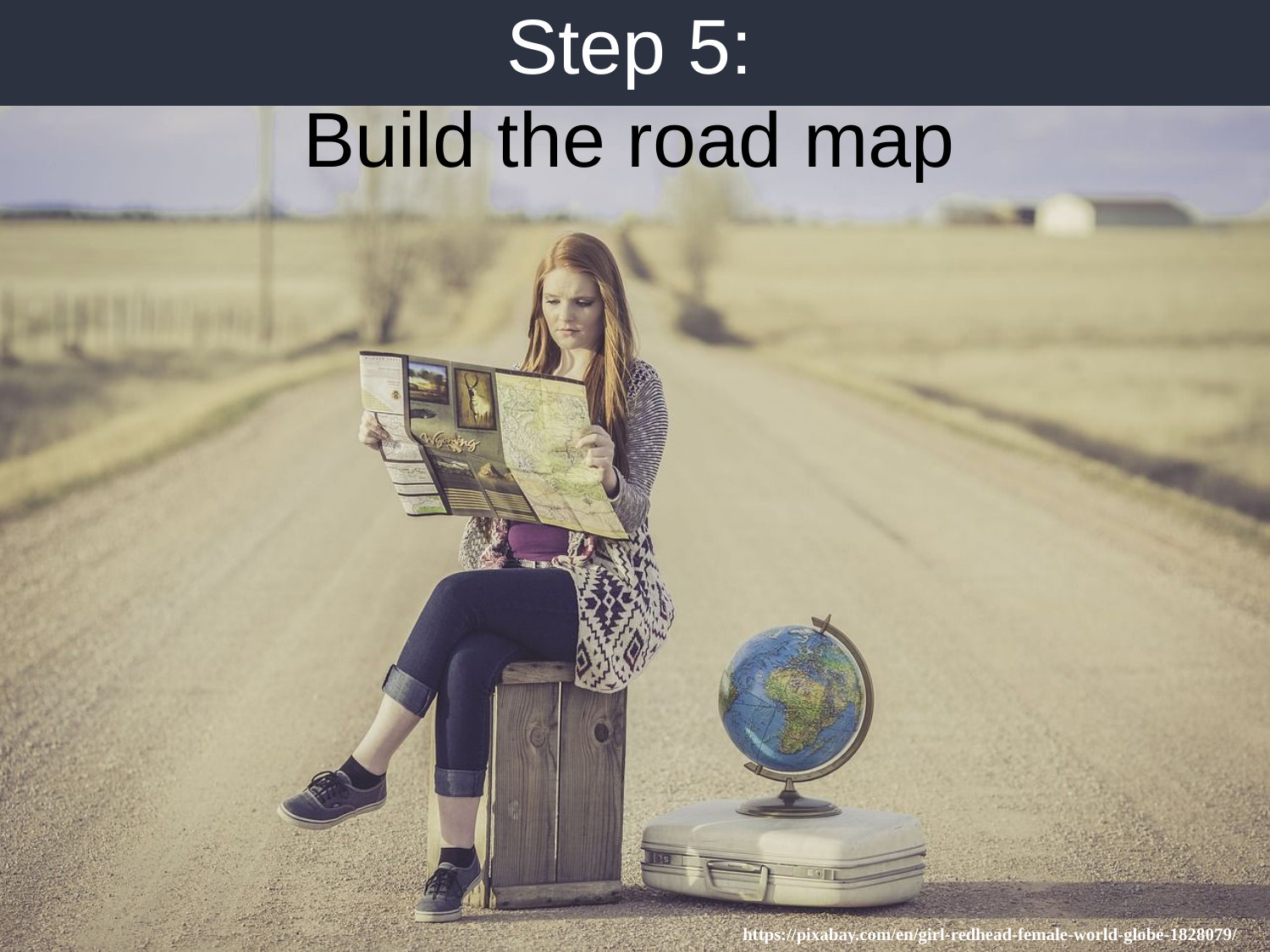

# Step 5: Build the road map
https://pixabay.com/en/girl-redhead-female-world-globe-1828079/

## Slide 28
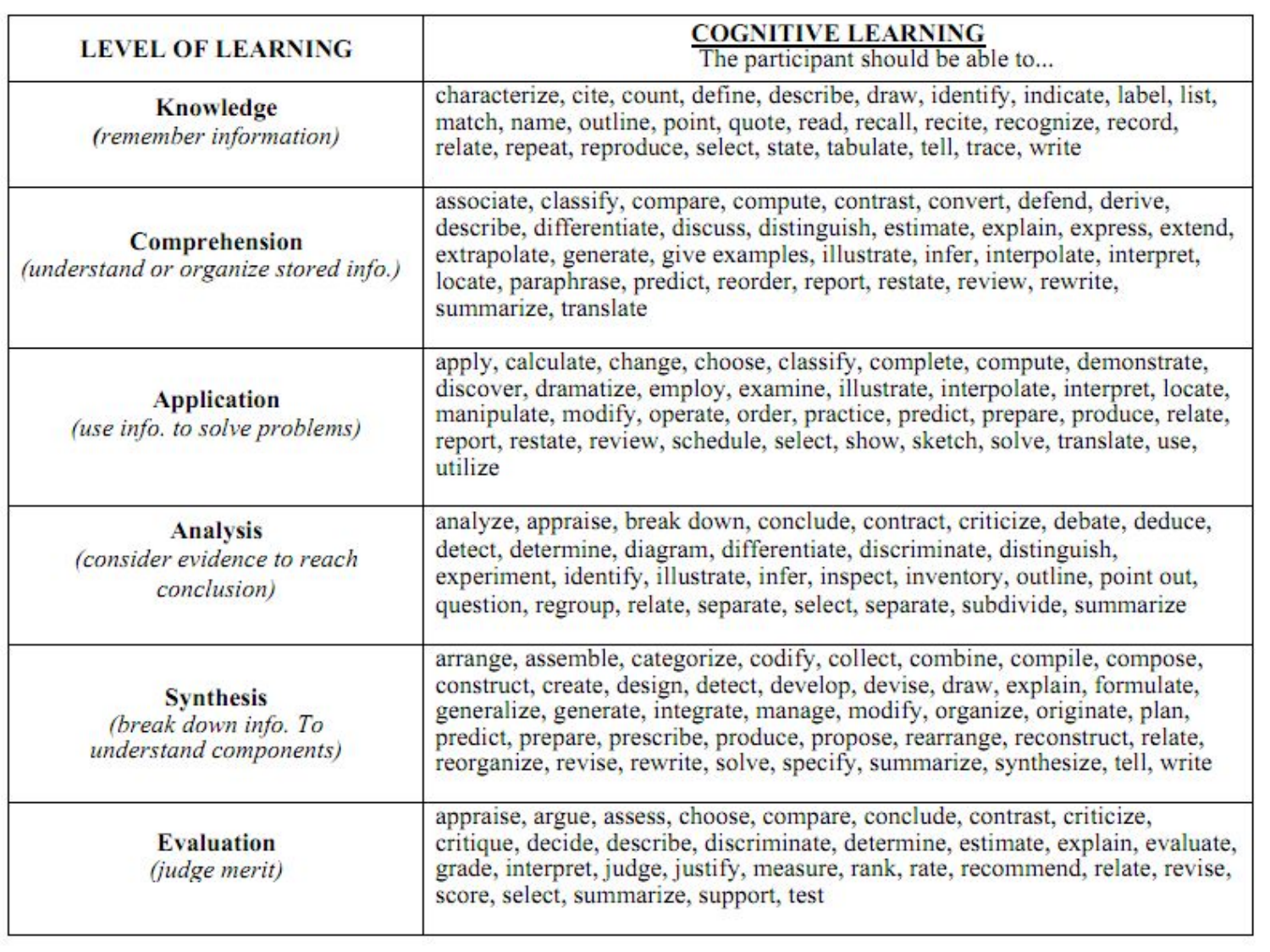

## Slide 29
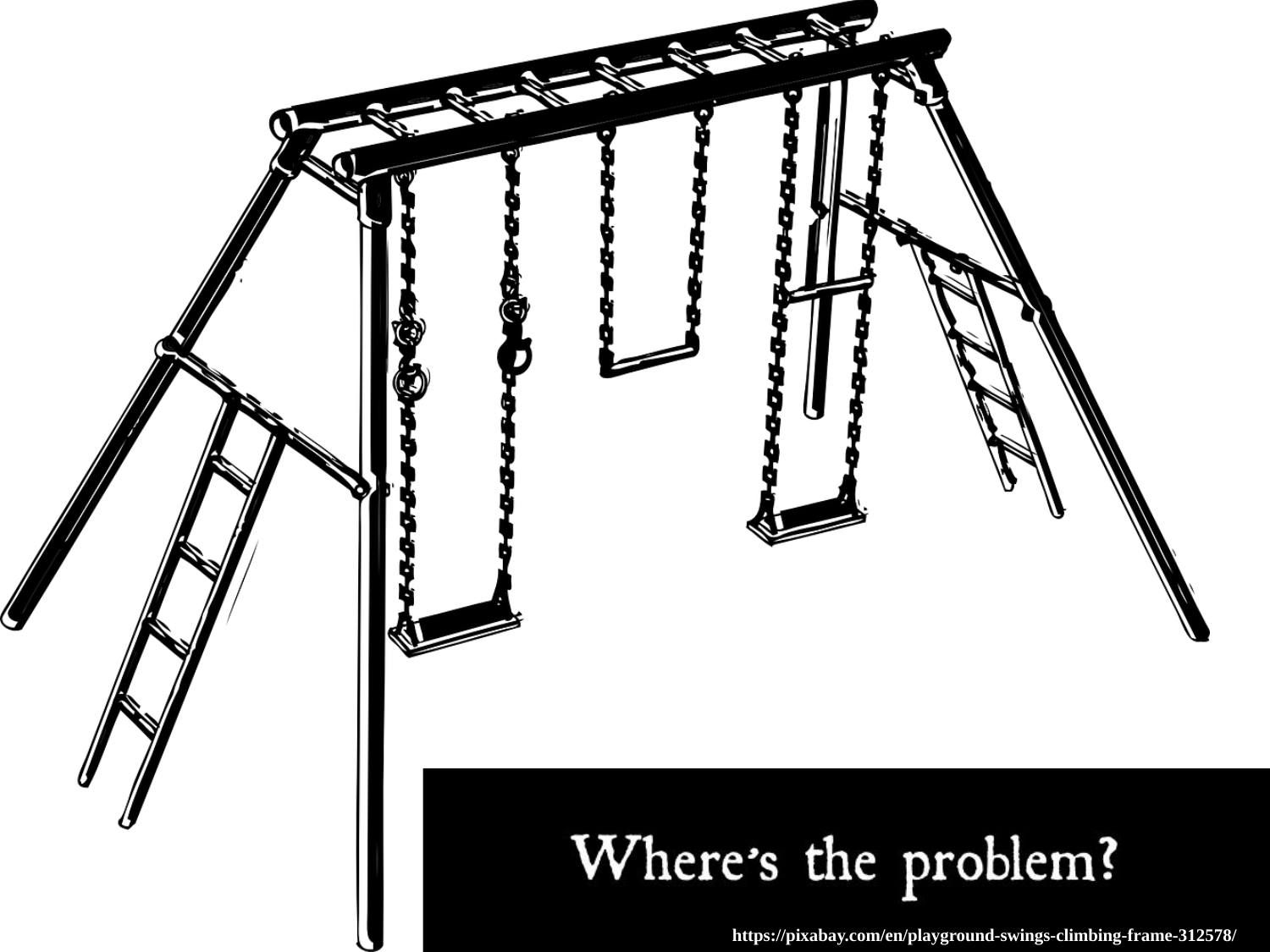

https://pixabay.com/en/playground-swings-climbing-frame-312578/

## Slide 30
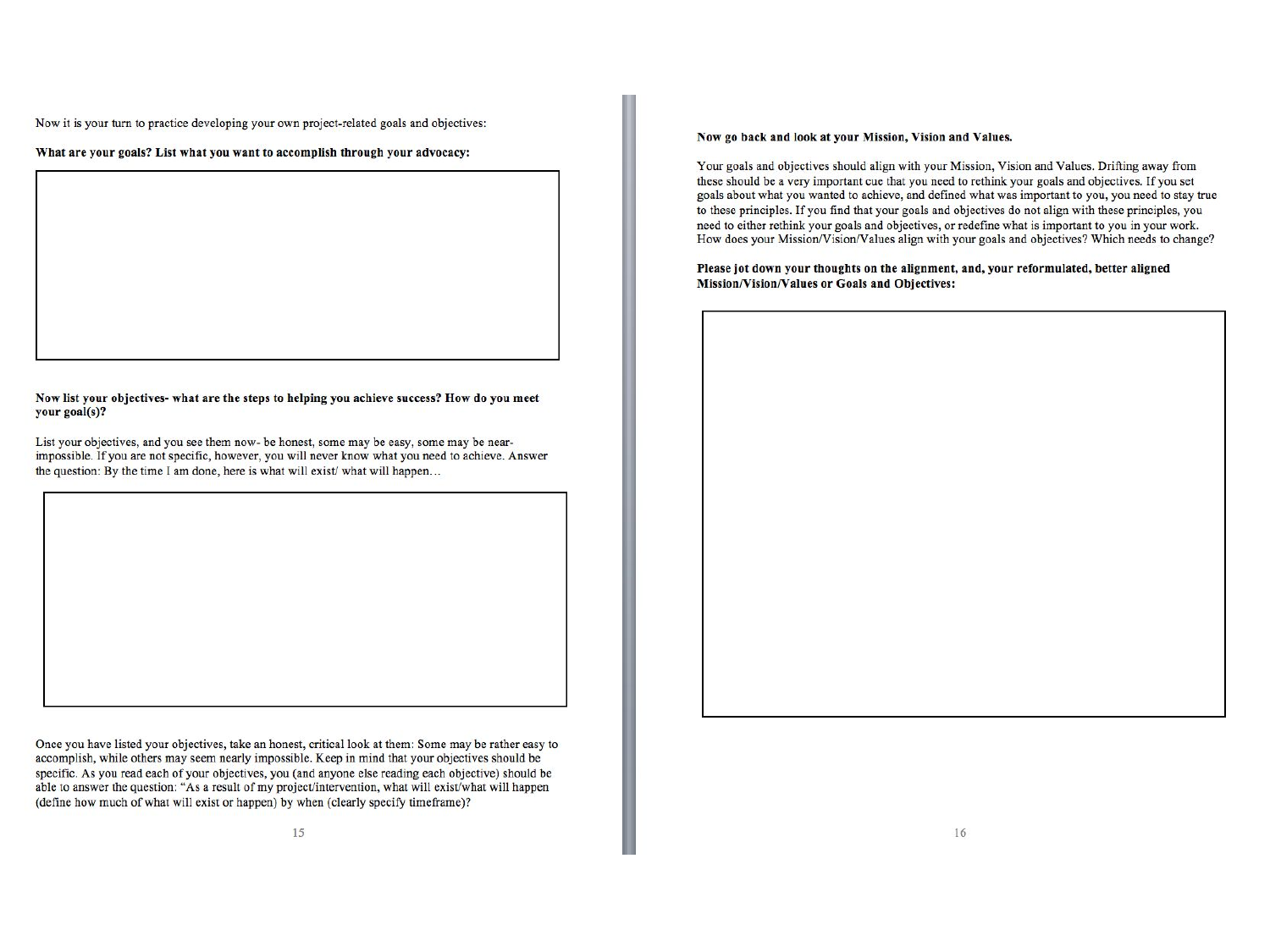

## Slide 31
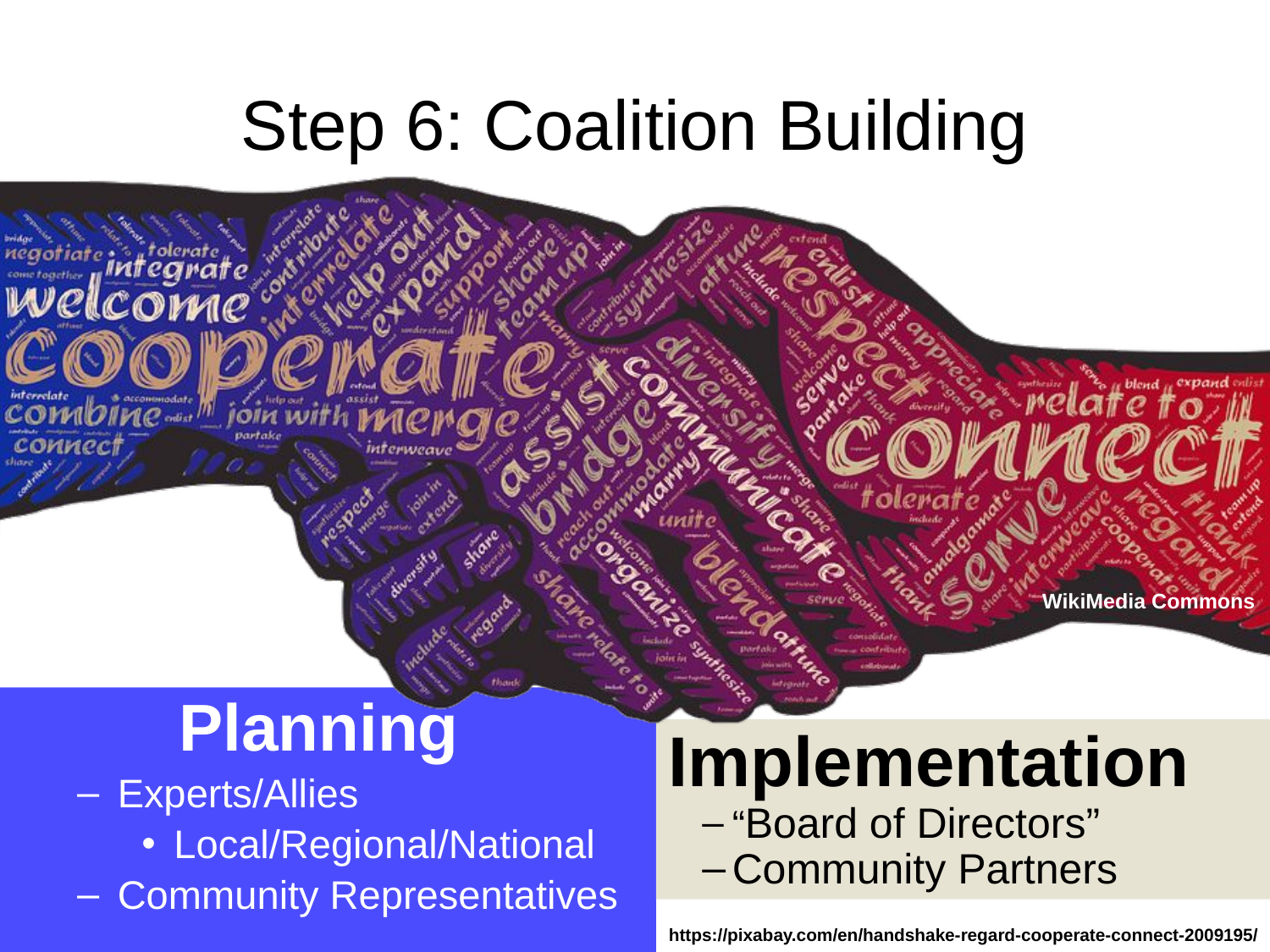

# Step 6: Coalition Building
WikiMedia Commons
Planning
Experts/Allies
Local/Regional/National
Community Representatives
Implementation
“Board of Directors”
Community Partners
https://pixabay.com/en/handshake-regard-cooperate-connect-2009195/

## Slide 32
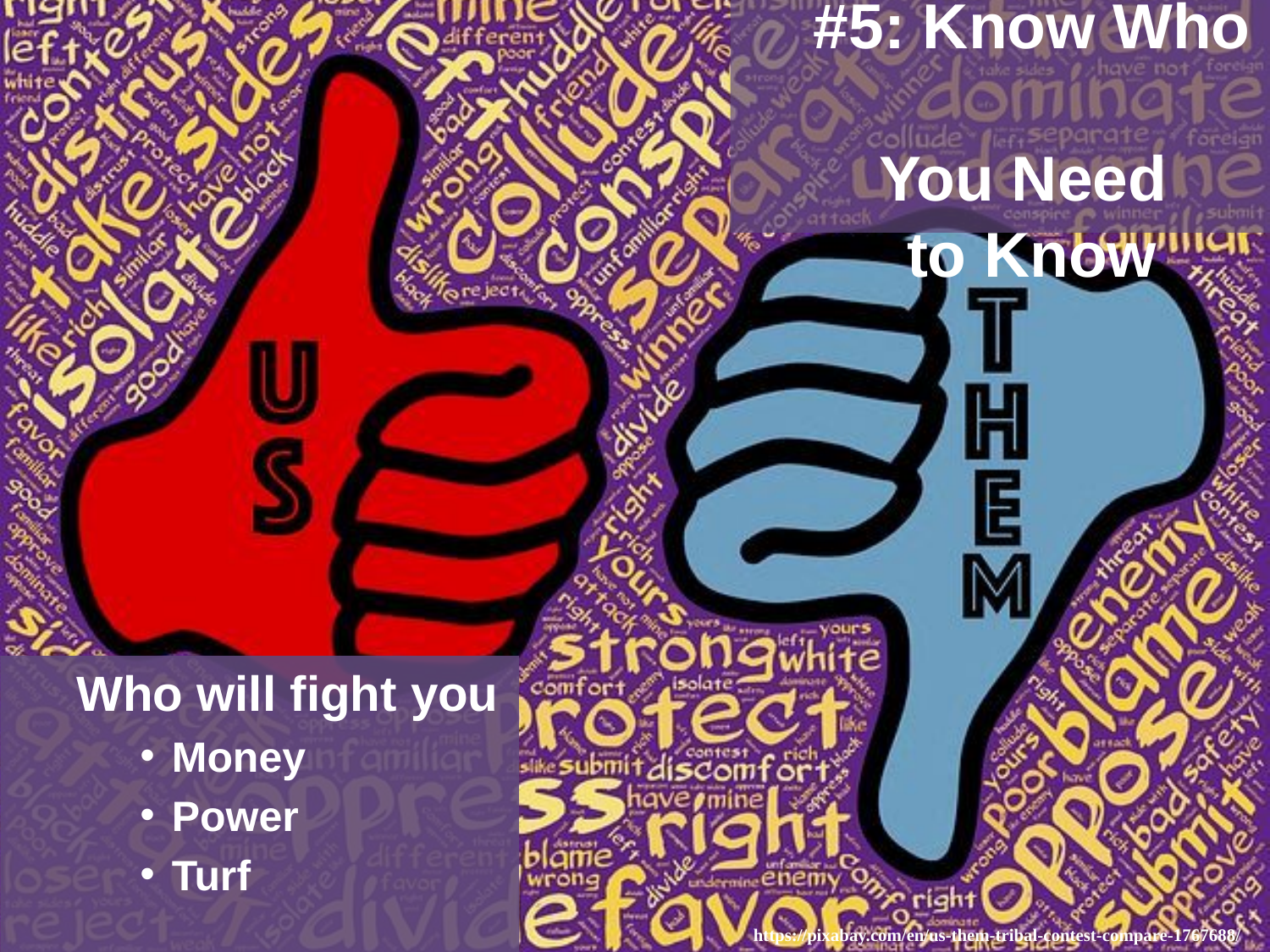

#5: Know Who
You Need
to Know
Who will fight you
Money
Power
Turf
https://pixabay.com/en/us-them-tribal-contest-compare-1767688/

## Slide 33
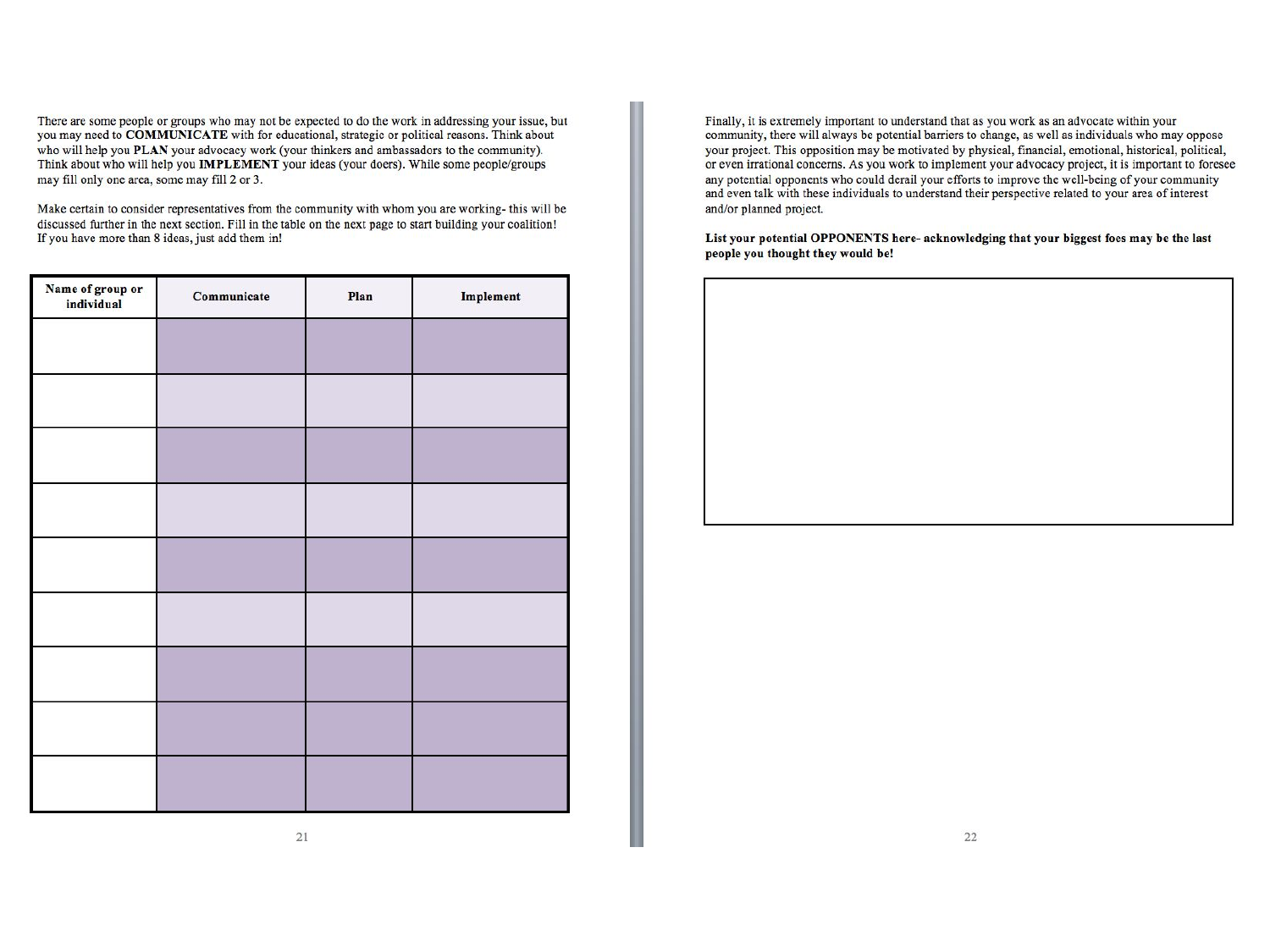

#

## Slide 34
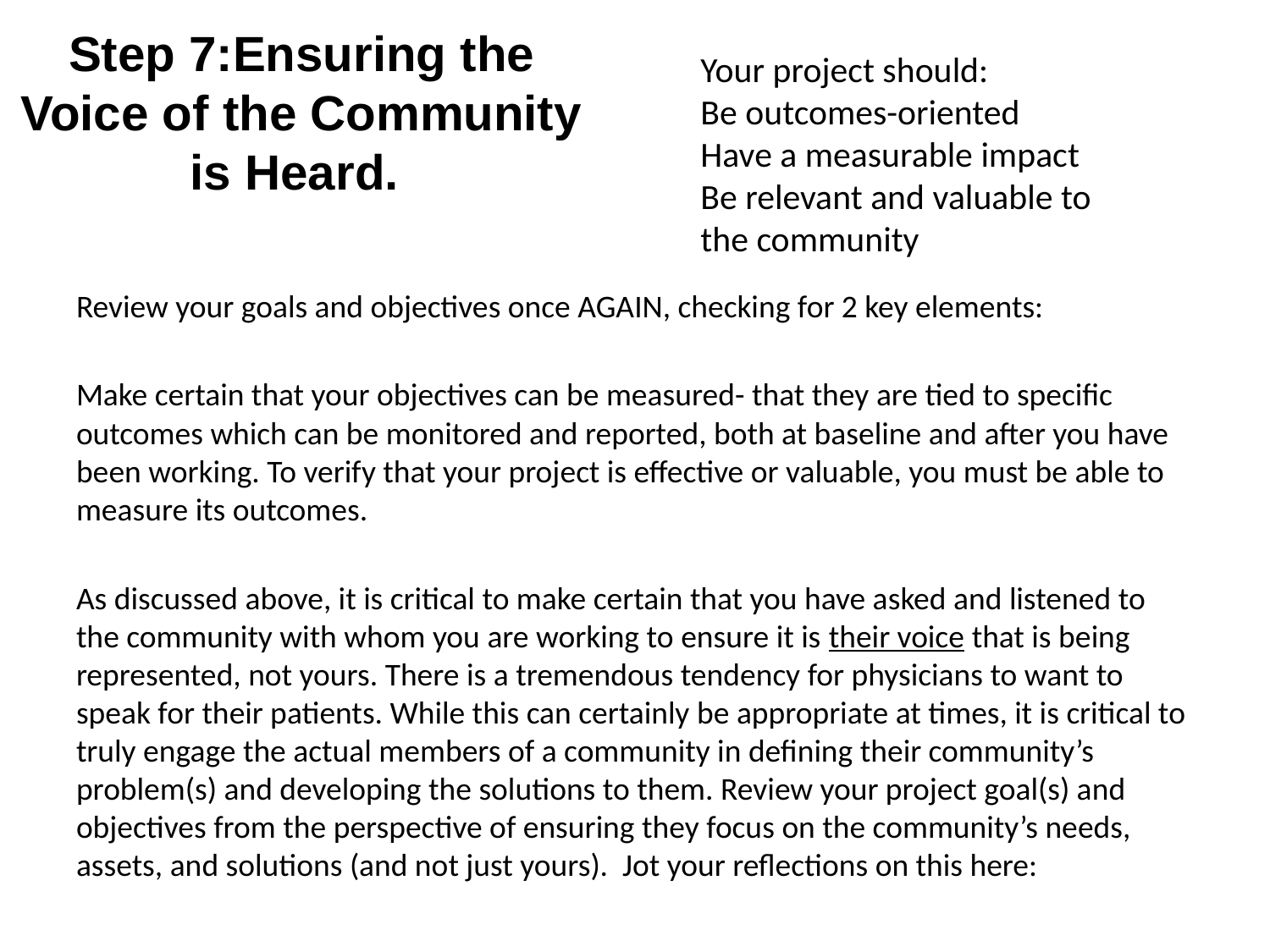

Step 7:Ensuring the Voice of the Community is Heard.
# Your project should: Be outcomes-oriented Have a measurable impact Be relevant and valuable to the community
Review your goals and objectives once AGAIN, checking for 2 key elements:
Make certain that your objectives can be measured- that they are tied to specific outcomes which can be monitored and reported, both at baseline and after you have been working. To verify that your project is effective or valuable, you must be able to measure its outcomes.
As discussed above, it is critical to make certain that you have asked and listened to the community with whom you are working to ensure it is their voice that is being represented, not yours. There is a tremendous tendency for physicians to want to speak for their patients. While this can certainly be appropriate at times, it is critical to truly engage the actual members of a community in defining their community’s problem(s) and developing the solutions to them. Review your project goal(s) and objectives from the perspective of ensuring they focus on the community’s needs, assets, and solutions (and not just yours). Jot your reflections on this here:

## Slide 35
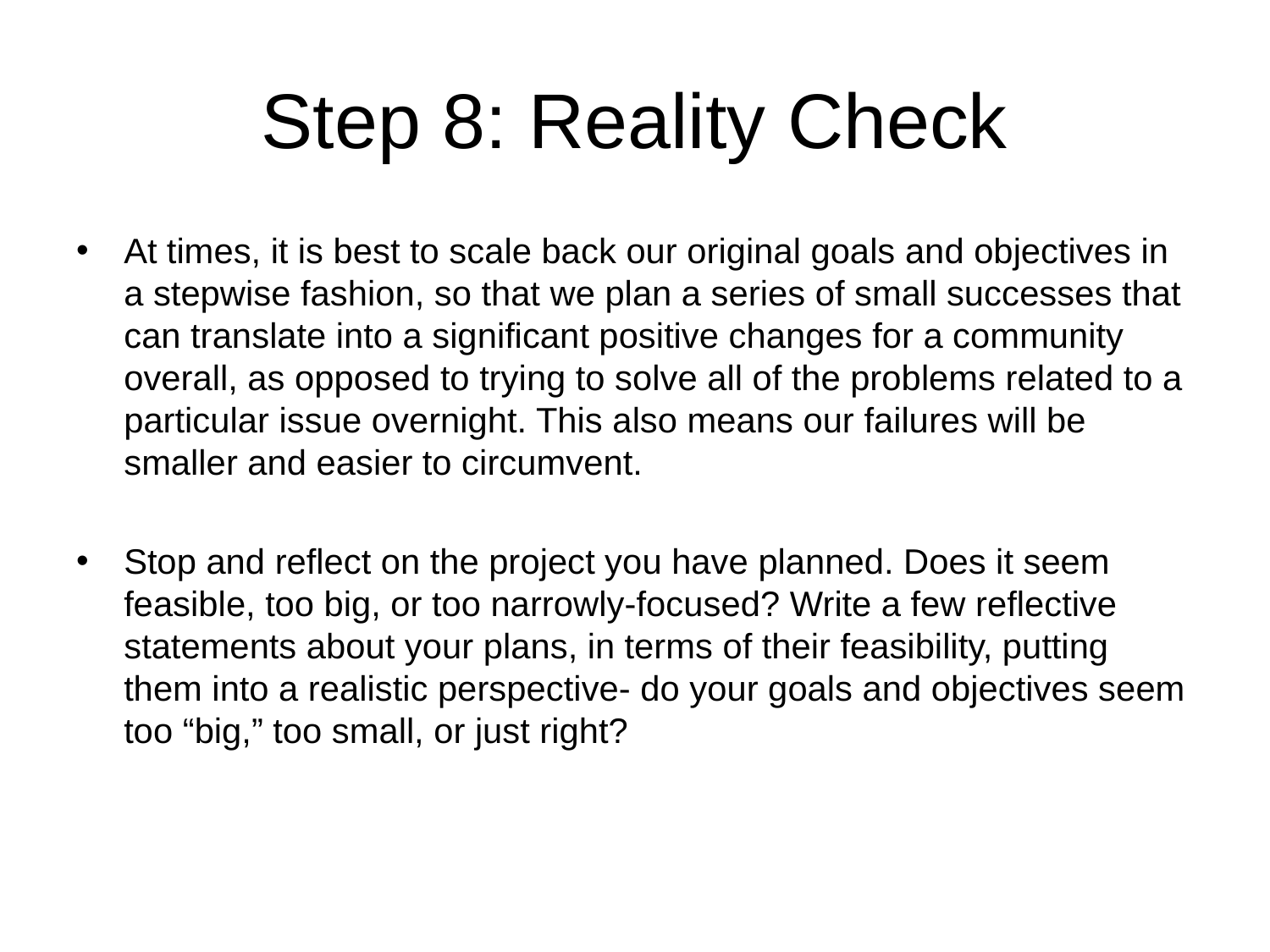

# Step 8: Reality Check
At times, it is best to scale back our original goals and objectives in a stepwise fashion, so that we plan a series of small successes that can translate into a significant positive changes for a community overall, as opposed to trying to solve all of the problems related to a particular issue overnight. This also means our failures will be smaller and easier to circumvent.
Stop and reflect on the project you have planned. Does it seem feasible, too big, or too narrowly-focused? Write a few reflective statements about your plans, in terms of their feasibility, putting them into a realistic perspective- do your goals and objectives seem too “big,” too small, or just right?

## Slide 36
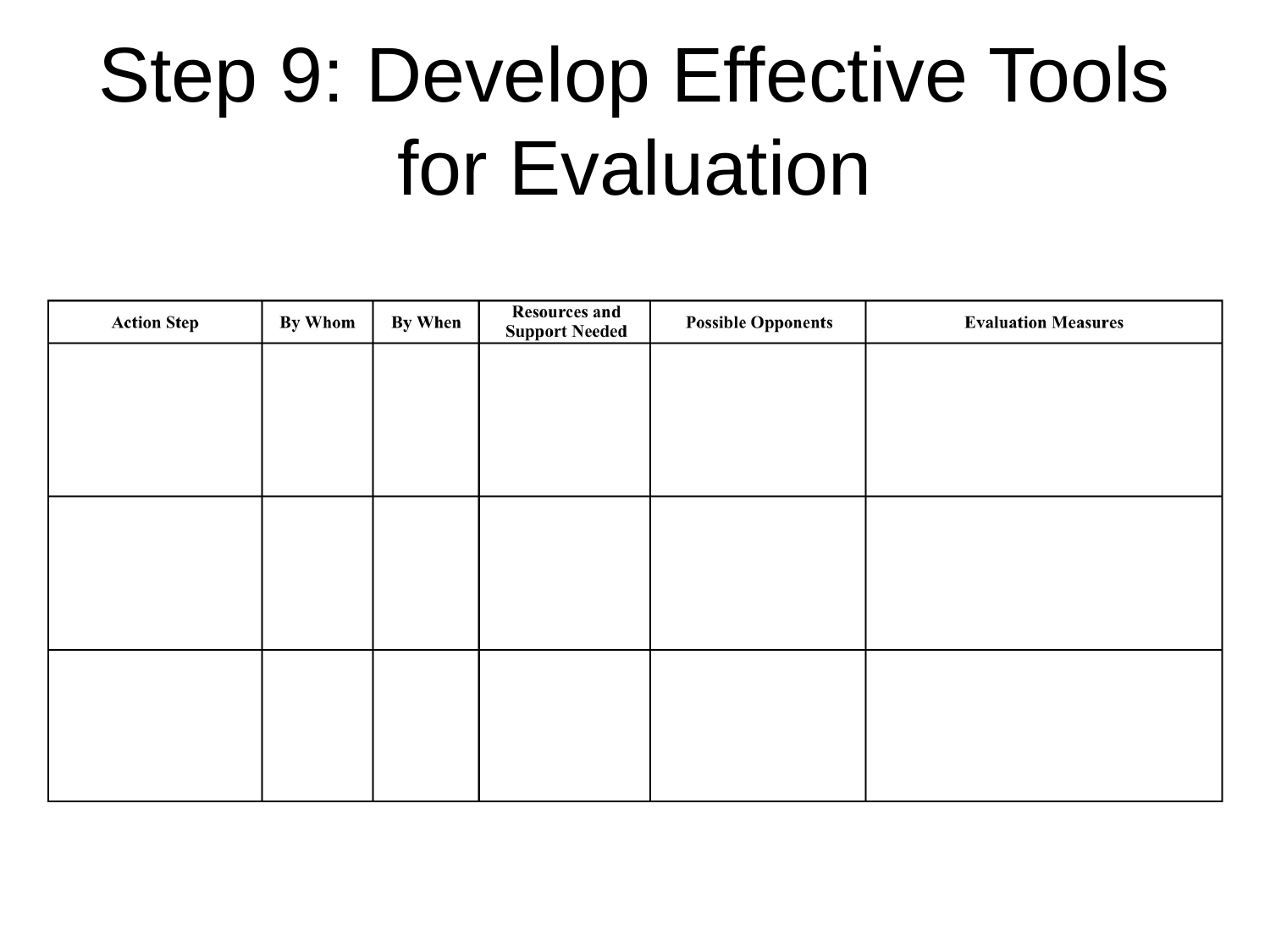

# Step 9: Develop Effective Tools for Evaluation

## Slide 37
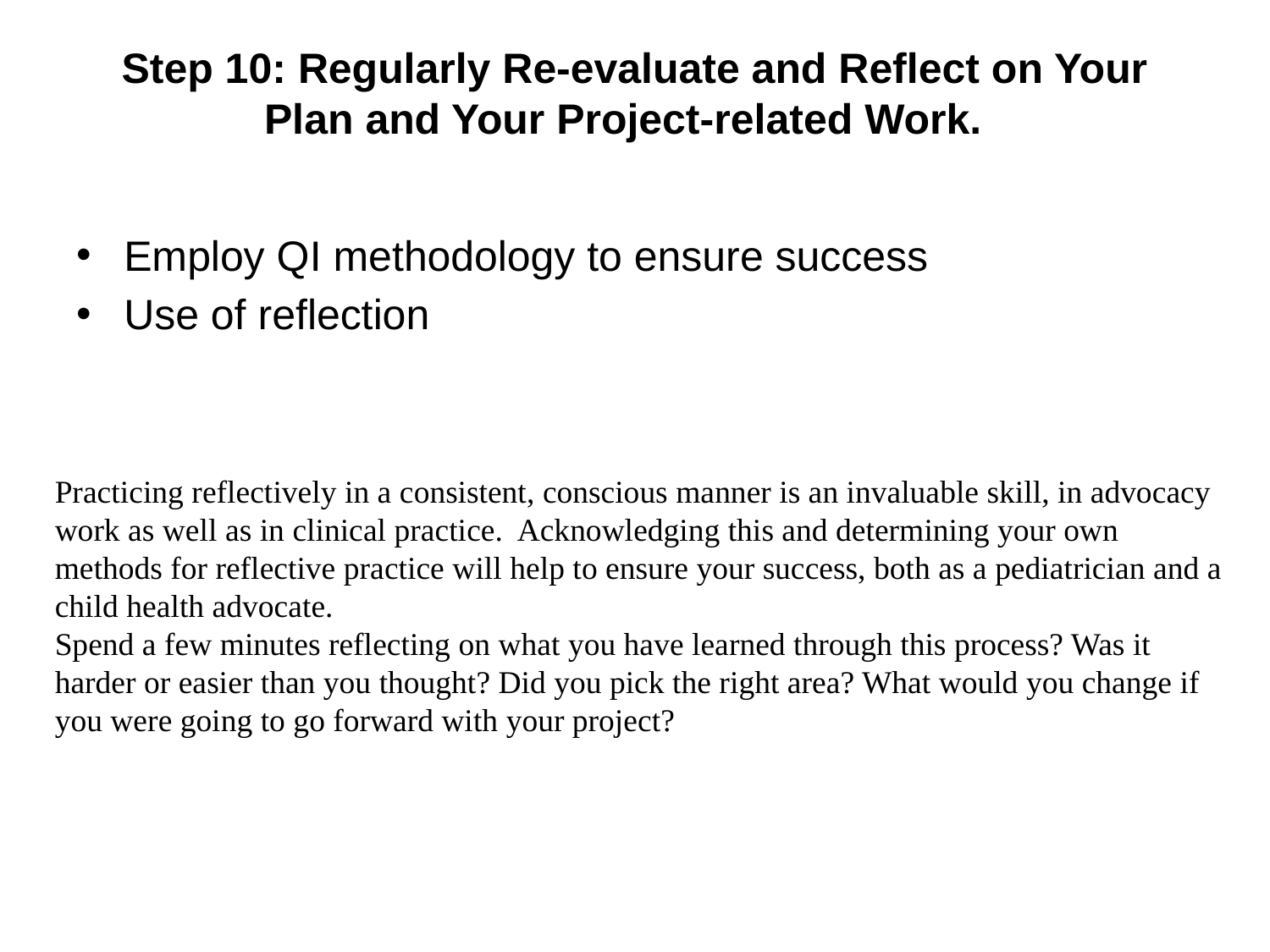

# Step 10: Regularly Re-evaluate and Reflect on Your Plan and Your Project-related Work.
Employ QI methodology to ensure success
Use of reflection
Practicing reflectively in a consistent, conscious manner is an invaluable skill, in advocacy work as well as in clinical practice. Acknowledging this and determining your own methods for reflective practice will help to ensure your success, both as a pediatrician and a child health advocate.
Spend a few minutes reflecting on what you have learned through this process? Was it harder or easier than you thought? Did you pick the right area? What would you change if you were going to go forward with your project?

## Slide 38
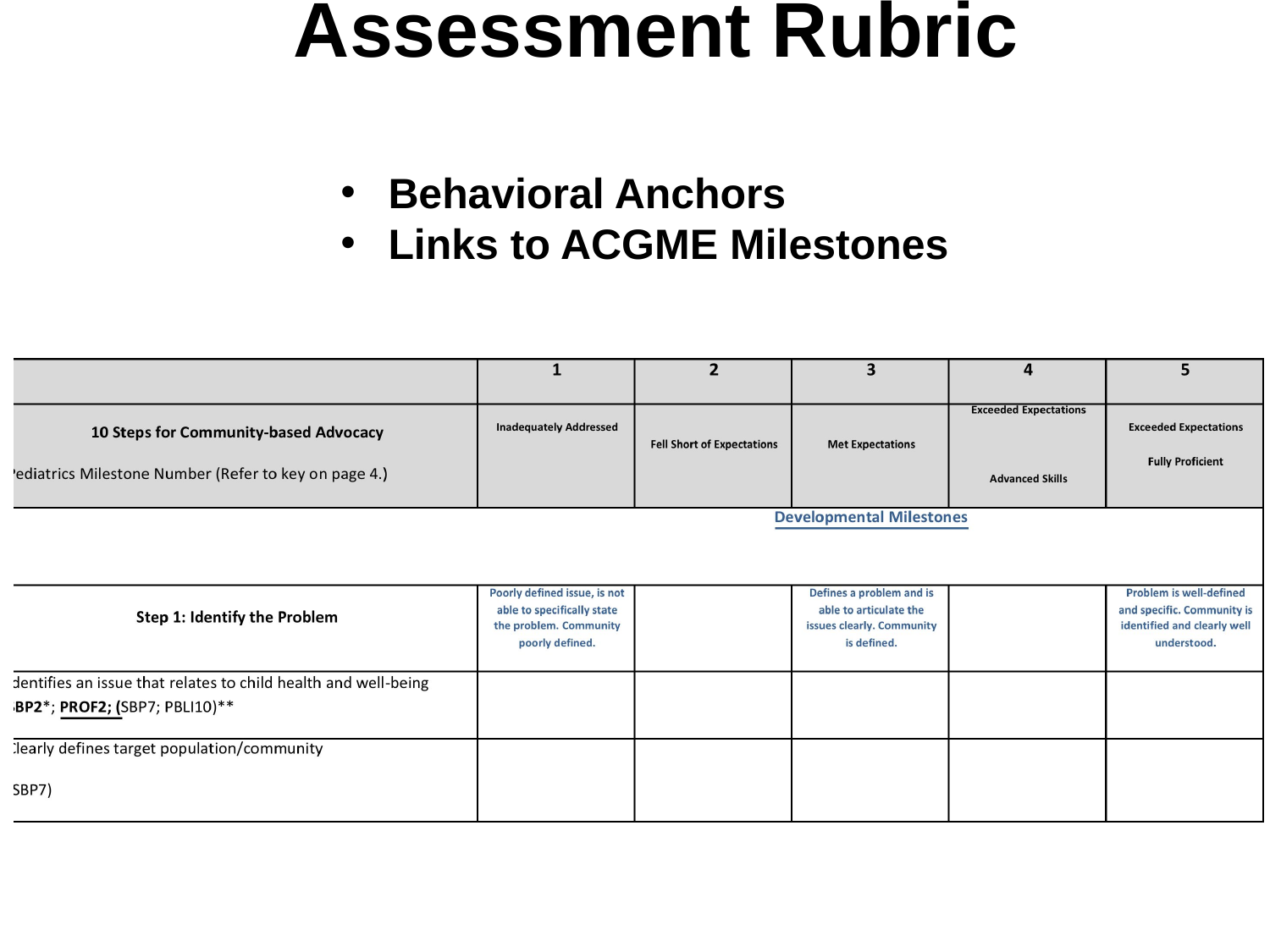

Assessment Rubric
Behavioral Anchors
Links to ACGME Milestones

## Slide 39
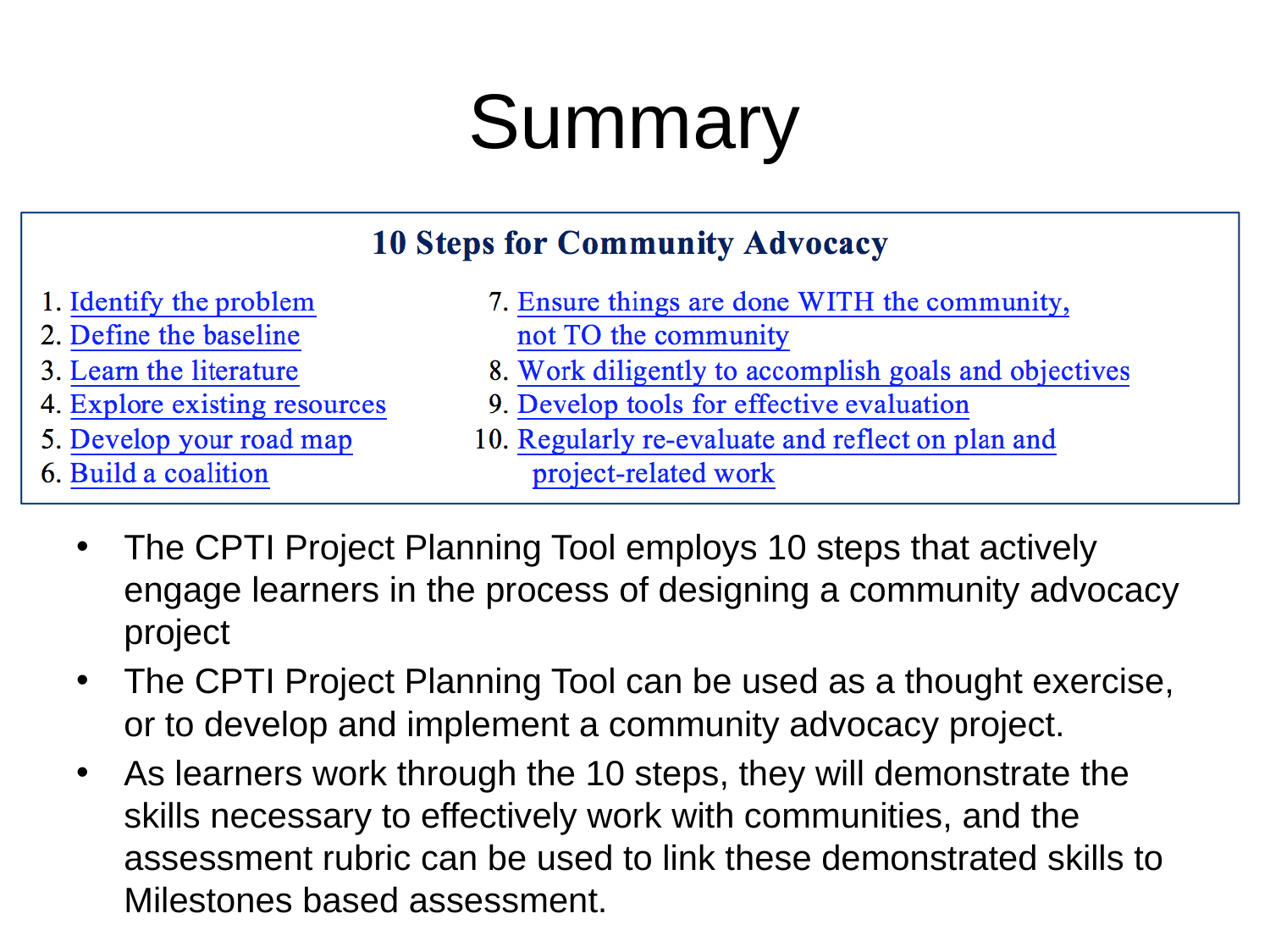

# Summary
The CPTI Project Planning Tool employs 10 steps that actively engage learners in the process of designing a community advocacy project
The CPTI Project Planning Tool can be used as a thought exercise, or to develop and implement a community advocacy project.
As learners work through the 10 steps, they will demonstrate the skills necessary to effectively work with communities, and the assessment rubric can be used to link these demonstrated skills to Milestones based assessment.
